# IL-1β/IL-6 inflammatory crosstalk drives DNA methylation and histone remodeling associated with cytokine induced senescence in luminal breast cancer

**DOI:** 10.1007/s00011-026-02391-7

**Published:** 2026-10-17

**Authors:** Satyajit Dey Pereira, Kanaka Sai Ram Padam, Padival Shruptha, Aditi Atreya, Manjunath B. Joshi, Kapaettu Satyamoorthy

**Affiliations:** 1 Department of Cellular and Molecular Biology, Manipal School of Life Sciences, Manipal Academy of Higher Education, Manipal, 576104 India; 2 SDM Centre for Cellular and Molecular Sciences, SDM College of Medical Sciences and Hospital, Shri Dharmasthala Manjunatheshwara (SDM) University, Dharwad, Karnataka 580009 India; 3 Department of Ageing Research, Manipal School of Life Sciences, Manipal Academy of Higher Education, Manipal, 576104 India; 4 Herman B Wells Center for Pediatric Research, Department of Pediatrics, Indiana University School of Medicine, Indianapolis, IN 46202 USA

**Keywords:** IL-1β, IL-6, Inflammation, Senescence, Methylation, Histone remodeling, Luminal breast cancer

## Abstract

**Background:**

Chronic inflammation exerts paradoxical effects in breast cancer, inducing growth arrest while sustaining tumor progression. However, how autocrine and paracrine inflammatory cytokine induced senescence integrates with tumor-promoting behaviour remains unclear. Here, we identify a reciprocal IL-1β/IL-6 circuit that links inflammatory cues, epigenetic remodeling and cytokine induced senescence in luminal breast cancer cells.

**Methods:**

Following treatment with IL-1β and IL-6, MCF-7 cells were examined using cell-cycle, apoptosis, reactive oxygen species flow cytometry assays, and senescence assays; RNA sequencing; global DNA methylation quantification; methylation-specific PCR; ChIP-qPCR for histone marks; cytokine ELISA; and western blotting for DNMTs and STAT3/AKT/NF-κB signaling proteins. The kinetics of IL-1β and IL-6 signaling were examined using receptor antagonism and pharmacological inhibition.

**Results:**

IL-1β induced senescence characterized by G_0_/G_1_ arrest, elevated SA-β-gal activity, ROS accumulation and absence of apoptosis. Transcriptomic profiling revealed cell-cycle regulator repression, enrichment of senescence-associated signatures and a pro-inflammatory secretory phenotype. Mechanistically, IL-1β induced increased DNMT1 and DNMT3A expression, elevated global DNA methylation, induced promoter-specific DNA methylation (*IL-15*, *HMGB2*) and histone mark remodeling (*IL-6*,* IL1RN*,* BCL2*,*BAX*) at inflammatory, senescence, and apoptotic loci. IL-1 receptor antagonism attenuated these effects. IL-6 induced downstream of IL-1β, in turn activated STAT3, increased DNMT1 and DNMT3B expression, amplified IL-1β expression and tumor-promoting phenotypes, while sustaining the IL-1β induced senescence state. IL-6R blockade disrupted this axis and reduced tumor-promoting effects.

**Conclusion:**

An IL-1β/IL-6 inflammatory loop drives epigenetic remodeling associated with cytokine induced senescence while promoting tumor-supportive signaling. IL-1β initiates the senescence program, whereas IL-6 reinforces and sustains this state through STAT3/DNMT signaling, highlighting inflammatory cytokine signaling pathways as potential therapeutic targets for breast cancer therapy.

**Supplementary Information:**

The online version contains supplementary material available at https://doi.org/10.1007/s00011-026-02391-7.

## Introduction

Chronic inflammation drives breast cancer pathogenesis, promoting tumor progression, therapeutic resistance, immunosuppression and metastasis [1, 2]. Among the numerous cytokines contributing to chronic inflammation and tumorigenesis, Interleukin-1β (IL-1β) and Interleukin-6 (IL-6) are recognized as central mediators of tumor-promoting inflammation. Serum levels of both IL-1β [3, 4] and IL-6 [5, 6] are elevated in breast cancer patients compared to normal tissue and are associated with poor prognosis [7, 8].

IL-1β is produced primarily by monocytes and macrophages [9]. It signals through a receptor complex comprising IL-1R1 (IL-1β receptor) and IL1RAP (IL-1β receptor accessory protein) which initiates a signaling cascade activating NF-κB, thereby driving transcription of numerous genes and downstream PI3K/AKT and STAT3 signaling pathways [10]. IL-1β signaling contributes to tumor progression by promoting metastasis, angiogenesis, production of inflammatory cytokines, cancer stemness, chemoresistance and immunosuppression [11–13]. On the other hand, IL-6 is produced primarily by immune cells, cancer-associated fibroblasts, endothelial cells and tumor cells [14]. It signals through either membrane bound or soluble IL-6R (Interleukin-6 receptor) in conjunction with the GP130 (Glycoprotein 130) co-receptor [15] that triggers phosphorylation, dimerization and activation of STAT3 which regulates transcriptional programs promoting immune evasion, apoptosis resistance, angiogenesis, tumor stemness and therapy resistance in breast cancer [16, 17].

IL-1β and IL-6 frequently function within interconnected inflammatory signaling networks in breast cancer. IL-1β acts as a strong upstream inducer of IL-6 and together they activate NF-κB and STAT3 signaling to promote invasion and metastasis in luminal and triple negative breast cancer models [18, 19]. Although direct IL-6 mediated induction of IL-1β has not been extensively characterized in breast cancer, the downstream transcriptional factors such as NF-κB and STAT3 are known to function in a cooperative manner to enable tumor promotion through anti-apoptotic, pro-proliferative and immunomodulatory transcriptional gene programs [20].

Cellular senescence is a stable growth-arrest state which can be induced by diverse stressors including oncogenic, replicative, inflammatory, epigenetic influences, mitochondrial dysfunction, chemotherapy and radiation therapy [21]. Chronic inflammatory cytokine exposure can result in cytokine induced senescence, frequently mediated through reactive oxygen species (ROS) generation, DNA damage responses and activation of p53-p21 signaling pathways [22]. While senescence is classically tumor suppressive, senescent cancer cells frequently acquire a senescence-associated secretory phenotype (SASP) characterized by secretion of inflammatory cytokines including IL-1β, IL-6, IL-8, CCL2, CCL5 and TNF-α that can reinforce senescence signaling and simultaneously promote tumor progression, immune evasion and chemoresistance [23, 24]. In addition to their paracrine effects on neighbouring tumour, stromal and immune cells within the tumour microenvironment, key SASP cytokines including IL-1β and IL-6, can also function through autocrine (cell-autonomous) signalling to reinforce and maintain the senescent phenotype [25]. Together, these paracrine and autocrine SASP signalling networks contribute to the persistence of senescence and chronic inflammation, which depending on biological context may influence tumour suppression or tumour progression [26].

Epigenetic reprogramming is a key mediator of senescence through promoter DNA hypermethylation, histone modifications and chromatin remodeling [66, 67]. IL-1β has been reported to induce senescence and initiate pro-inflammatory SASP via NF-κB in several cancers [27, 28]. In parallel, IL-6 contributes to the maintenance of a stable senescent state while simultaneously promoting tumor progression in breast cancers [29]. Emerging evidence suggests that both IL-1β and IL-6 can modulate the tumor epigenome. IL-1β has been reported to induce DNA promoter methylation and histone remodeling of E-cadherin, promoting Epithelial-mesenchymal transition (EMT) in non-small cell lung cancers [30] and alter the methylation status of IL-6, IL-8 and IL-10 cytokine promoters in colorectal cancers [31]. Whereas, IL-6 has been reported to influence DNMT1 stability in breast cancers through PI3K/AKT signaling resulting in altered VEGFR2 expression and disordered angiogenesis [32].

These observations suggest that chronic IL–1β/IL-6 inflammatory signaling may reprogram the epigenetic landscape of breast cancer cells driving cytokine induced senescence while simultaneously promoting tumor progression. However, the mechanistic basis of this inflammation driven epigenetic remodeling remains poorly understood particularly in luminal breast cancer subtypes. To address this, we examined how chronic IL-1β and IL-6 signaling in luminal breast cancer cells establishes cytokine induced senescence, accompanied by coordinated SASP activation and epigenetic remodeling. Specifically, IL-1β initiates senescence associated epigenetic remodeling, while IL-6 reinforces DNMT dependent tumor supportive phenotypes and contributes to the maintenance of the IL-1β induced senescence phenotype.

## Materials and methods

### Data retrieval and analysis

The pathobiological expression of *IL1B* and *IL6* across immune, stromal and malignant cell populations within the breast tumor microenvironment (TME) was profiled by accessing public single cell RNA sequencing (scRNA-seq) datasets through TISCH2 database [33]. RNA expression levels (nTPM) of *IL1B*, *IL1R1* and *IL1RAP* across breast epithelial non tumorigenic and breast cancer cell lines of different molecular subtypes were obtained from the Human Protein Atlas (HPA) cell line transcriptomics dataset [34]. Further, the expression profiles of the *IL-1R1* and *IL1RAP* receptors across normal breast tissue (*n* = 108) and breast cancer molecular subtypes (Luminal *n* = 454, HER2-enriched *n* = 31, triple negative *n* = 113) were retrieved from The Cancer Genome Atlas (TCGA) [35] using UCSC Xena Browser [36]. Recurrence-free survival (RFS) analysis based on *IL1R1* and *IL1RAP* expression was assessed using KM Plotter (https://kmplot.com/analysis) [37] to access publicly available breast cancer cohorts. Patients were stratified into high and low expression groups based on median cut-off. Spearman rank correlation (*ρ*) between *IL-1β* and *IL-6* mRNA expression was computed for the TCGA-BRCA cohort and for Luminal A subtype tumors.

### Cell culture and cytokine treatment conditions

MCF-7 (CVCL_0031), MDA-MB-231 (CVCL_0062) and MCF-10 A (CVCL_0598) cell lines were procured from ATCC and cultured according to supplier’s protocol. All cell lines were maintained at 37 °C in a 5% CO_2_ incubator. Cell lines were authenticated through STR profiling. Recombinant cytokines were reconstituted and stored according to manufacturer’s instructions. MCF-10 A, MCF-7 and MDA-MB-231 cells were serum starved with 1% FBS (Gibco, USA) for 24 h and then treated with 10ng/mL IL-1β (PeproTech, USA) for 24–72 h periods, with cytokine replenishment every 24 h. IL-1β antagonistic experiments for MCF-7 cells were carried out with the following groups being maintained - untreated control, IL-1β (10 ng/mL), IL-1β (10 ng/mL) + IL-1Ra (20 ng/mL) (PeproTech, USA) and IL-1Ra (20 ng/mL) for 24 h and 72 h durations. For washout experiments, cells were treated with IL-1β (10 ng/mL) for 72 h, followed by culture in cytokine free medium for an additional 72-hours. To assess downstream signaling effects of IL-6, MCF-7 cells were treated with 25 ng/mL of IL-6 (PeproTech, USA) for 24 h.

For conditioned media experiments, culture supernatants were collected from MCF-7 cells following 72 h of treatment with the indicated cytokine conditions, centrifuged to remove cellular debris and then applied directly to fresh recipient MCF-7 cells for 24 h treatment in the presence or absence of humanized huE17 anti-IL-6R antibody (100 ng/mL; generated in-house) [38] as indicated prior to downstream functional assays.

### Reverse transcription qPCR

Total RNA was isolated from MCF-10 A, MCF-7, MDA-MB-231 cells and cytokine treated and untreated control MCF-7 cells using PureLink RNA Mini Kit (Thermo Fisher, USA) and cDNA was synthesized using the High-capacity cDNA reverse transcription kit (Applied Biosystems, USA). qPCR was performed using PowerUp SYBR Green Master Mix (Applied Biosystems, USA) in Quant Studio 6 Pro (Applied Biosystems, USA) to assess *IL1R1*,* IL1RAP*,* IL6R* and *GP130 (IL6ST)* in MCF-10 A, MCF-7 and MDA-MB-231 and *HMGB2* and *CDKN1A* expression in control and cytokine treated MCF-7 cells. The relative gene expression levels were quantified with β-actin as endogenous control using 2^−ΔΔCt^ method [39]. Primer sequences used for all qPCR assays are provided in Supplementary Table S1.

### Cell cycle analysis

MCF-7 cells were serum starved for 24 h and subsequently treated with either – IL-1β (10 ng/mL), IL-1β (10 ng/mL) + IL-1Ra (20 ng/mL) or IL-1Ra (20 ng/mL) for 24 and 72 h. Untreated control cells were maintained in parallel. For conditioned media (CM) experiments, naïve recipient MCF-7 cells were treated for 24 h with CM derived from the above treatment groups. An additional group received IL-1β CM in the presence of the humanized anti-IL-6R antibody huE17. The cultured cells were harvested, fixed with ethanol overnight, treated with RNase A and stained with propidium iodide (50 µg/mL). Distribution of cell cycle phases was assessed using CyFlow Space flow cytometer (Sysmex, Germany).

### Cell proliferation assay

Cell counting kit-8 (CCK-8) assay (Dojindo, Japan) was used to assess proliferative effects of cytokine induction on MCF-7 breast cancer cells. Briefly, 1 × 10^4^ MCF-7 cells were seeded per well, serum starved for 24 h and then treated with cytokines or conditioned media as described above. Subsequently, following the treatments, WST-8 reagent was added per well and incubated for 4 hours at 37 °C. Absorbance was measured at 450 nm using Varioskan flash microplate reader (Thermofisher, USA).

### EdU incorporation assay

DNA synthesis in MCF-7 cells was assessed using Click-iT EdU microplate assay kit (Invitrogen, USA). About 1 × 10^4^ MCF-7 cells were seeded per well in 96-well plates, serum starved for 24 h and then treated with cytokines or conditioned media as described earlier. Post-treatment the cells were exposed to EdU (10 µM) in culture media for 3 h and subsequently fluorescence (expressed as relative fluorescence units, RFU) was measured at 490 nm excitation/585nm emission using Varioskan flash microplate reader (Thermofisher, USA).

### Annexin V-Elab fluor 488/PI apoptosis assay

Annexin V-Elab Fluor 488/PI kit (Elabscience, USA) was used to estimate early and late phase apoptotic cell populations. Briefly control, IL-1β (10 ng/mL), IL-1β (10 ng/mL) + IL-1Ra (20 ng/mL) or IL-1Ra (20 ng/mL) treated MCF-7 cells (24 and 72 h) were resuspended in binding buffer and treated with Annexin V-Elab Fluor 488 and Propidium iodide (Sigma-Aldrich) at room temperature for 15 min. Cells were analyzed using FL1/FL3 channels on a flow cytometer (CyFlow Space, Sysmex, Germany).

### RNA sequencing and differential gene expression analysis

Total RNA was isolated from untreated control, IL-1β (24- and 48- hours), and IL-6 treated MCF-7 cells (24-hours) and untreated control and IL-1β (72 h) MDA-MB-231 cells using the PureLink RNA Mini Kit (Thermofisher, USA) and subjected to concentration and quality verification. Following this paired-end library preparation (150 bp x 2, 25 million reads) was carried out using NEBNext Ultra II RNA Library Prep Kit (NEB, USA). Ribosomal RNA (rRNA) depletion was performed using biotinylated target specific oligonucleotides followed by removal with magnetic beads (RiboMinus™ Eukaryote Kit, Thermofisher, India). The depleted RNA was subsequently purified, and first strand cDNA synthesis carried out using random hexamer primers. Second-strand cDNA was then generated by incorporating uracil-containing nucleotides during second-strand synthesis, followed by uracil-specific excision reagent (USER) digestion to preserve transcript orientation. Sequencing libraries were indexed using limited-cycle PCR amplification, purified using AMPure magnetic bead-based cleanup (Beckman Coulter, USA).

mRNA sequencing was carried out using the Illumina HiSeq4000/X platform. Raw reads were quality assessed using MultiQC, and low-quality reads (Phred score < 30) and adapter sequences removed using Trimmomatic, aligned to GRCh37/hg19 using HISAT2 and read counts mapped to the genome were obtained using Feature Counts. Differential gene expression analysis was performed using EdgeR package v3.22.2 [40] with a threshold of log_2_FC (≤ -1 and ≥ + 1) and FDR < 0.05. Genes were classified into gene families based on their biological functions and were visualized using ComplexHeatmap R package [41]. Differentially expressed genes were subject to Kyoto Encyclopedia of Genes and Genomes (KEGG) pathway [42] and gene set enrichment analysis using the Cluster Profiler R package [43] with statistical significance determined by an adjusted *p*-value (< 0.05).

### Western blotting

Total protein was extracted from control only for baseline receptor profiling or control and cytokine treated MCF-10 A, MCF-7 and MDA-MB-231 cells using RIPA lysis buffer system (Santa Cruz Biotechnology, USA). Total protein was estimated using Pierce BCA Protein Assay kit (Thermofisher, USA) and 20 µg of protein was resolved by SDS-PAGE and transferred onto methanol-activated PVDF membranes (Bio-Rad, USA). Post blocking, the membranes were probed overnight at 4℃ with primary antibodies and then incubated with appropriate secondary antibodies. The primary and secondary antibodies used are listed in Supplementary Table S2. The blots were visualized using Clarity Western ECL Substrate (Bio-Rad, USA) on iBright CL1500 Imaging system (Thermofisher, USA ).

### Global methylation analysis by HPLC

Genomic DNA was isolated from untreated control and cytokine treated MCF-10 A, MCF-7 and MDA-MB-231 cells using DNeasy Blood and Tissue DNA extraction kit (Qiagen, Germany). The isolated genomic DNA samples were prepared as described previously with sequential enzymatic digestion using DNase 1 and P1 nuclease, along with denaturation by heating at 95℃ and alkaline phosphatase resulting in breakdown of double-stranded genomic DNA to nucleosides [44]. Reverse-phase HPLC (Waters Alliance 2695, MA, USA) using a C18 column (Phenomenex, USA) was used to quantify global 5mC levels utilizing the program as described previously [45]. Detection was carried out using Waters 2487 dual Wavelength absorbance detector. Purified deoxynucleoside standards were run in different concentrations along with the samples to generate a standard curve. The percentage of 5-methylcytosine was calculated relative to total cytosine content (% 5mC = peak area of 5 mC/[peak area of 5mC+peak area of C] x100).

### Integrative transcriptomic methylation analysis and candidate prioritization

Differentially expressed genes from cytokine-treated MCF-7 transcriptomes were integrated with the NCI-60 Human Tumor Cell Lines Methylation Profile for MCF-7 [46]. For the methylation dataset, β-values of CpG probes located − 1500 bp upstream to + 500 bp downstream of annotated transcription start sites were averaged per gene and β-value ≥ 0.65 or β ≤ 0.35 defined hypermethylated and hypomethylated genes respectively [47]. Differentially expressed genes were classified as either potential methylation candidates, if hypomethylated in the NCI-60 methylation dataset and downregulated in the transcriptomic dataset or potential demethylation candidates, if hypermethylated in the NCI-60 dataset and upregulated in the transcriptomic dataset. These candidate gene sets were subjected to functional enrichment analysis using EnrichR [48] and clusterProfiler R package [43].

### Integration of senescence database signatures with transcriptomic and methylation candidates

Differentially expressed genes from the IL-1β transcriptome were cross-referenced with the CellAge: Human Cell Senescence Gene Database (Build 2.0) [49] to identify senescence-associated transcriptional signatures. Senescence-downregulated genes were then compared with the potentially methylated candidate genes list to identify overlapping genes exhibiting both transcriptional repression and promoter methylation potential and associated with the senescence phenotype. Overlaps were visualized via Venn diagrams constructed using InteractiVenn [50].

### Histochemical and flow cytometric assessment of senescence

Cytokine induced senescence was assessed using the senescence-associated β-galactosidase (SA-β-gal) assay as previously described [51]. Briefly, MCF-7 cells were subjected to cytokine treatments as described above for 72 h. For IL-1β pathway inhibition experiments, cells were treated with IL-1β in the presence or absence of IL-1 receptor antagonist (IL1RA). For IL-6 signaling experiments, cells were treated with IL-1β, IL-6 or their combination in the presence or absence of IL-6R blockade (huE17). Following treatment, the cells were fixed in 4% paraformaldehyde (Sigma-Aldrich, USA) and then stained with X-gal (40 mg/mL) (HiMedia, India) solution in citrate buffer (pH 6.0). Blue-green stained cells were counted manually under a bright-field microscope using a 10× objective. For quantification, 10 random fields per condition were imaged, and the percentage of SA-β-gal–positive cells was calculated relative to the total number of cells. Flow cytometric assessment of senescence was performed using the CellEvent™ Senescence Green Flow Cytometry Assay Kit (Thermo Fisher Scientific, USA) according to the manufacturer’s instructions. Following the respective cytokine treatments, cells were harvested, fixed with 2% paraformaldehyde for 10 min (Sigma-Aldrich, USA), stained with the CellEvent™ Senescence Green Probe for 90 min, and analyzed by flow cytometry using a 488-nm laser and 530-nm/30 filter .

### ROS flow cytometry

Intracellular ROS levels were measured using the fluorescent probe 2′,7′-dichlorofluorescin diacetate (DCFDA) as previously described [52] to assess oxidative stress associated with cytokine induced senescence. Briefly, MCF-7 cells were treated with IL-1β (10 ng/mL) in the presence or absence of IL-1Ra (20 ng/mL) (24 and 72 h ) with untreated control cells maintained in parallel. Subsequently cells were stained with DCFDA (1 µM) (Thermofisher, USA) and analyzed in a CyFlow Space flow cytometer (Sysmex, Germany).

### Cytokine multiplex ELISA assay

A multiplex magnetic microsphere bead panel was utilized to quantify IL-1β, IL-6, IL-1Ra, IL-15, CCL2, CXCL1, CXCL8, and TNF-α cytokine levels in culture supernatants collected from the MCF-10 A, MCF-7, MDA-MB-231 cells for basal cytokine profiling, from MCF-7 cells post cytokine treatments (R&D Systems; USA) and from conditioned media or post wash media where indicated. Samples were processed according to the manufacturer’s instructions and captured and analyzed using the Luminex Magpix instrument (Luminex Corporation, USA).

### Methylation specific PCR

Methylation-specific PCR (MSP) was performed to evaluate effects of IL-1β on DNA methylation status of the *IL-15* and *HMGB2* promoter regions in MCF-7 cells following 24 h and 72 h treatment. The primers used for the MSP were designed using the MethPrimer tool [53] and are listed in the Supplementary table S3. Genomic DNA was isolated using DNeasy Blood and Tissue DNA extraction kit (Qiagen, Germany) and Bisulfite conversion carried out using EZ DNA Methylation-Direct (Zymo Research, USA) kit. 50 ng of converted DNA was used as a template for MSP as previously described [54]. Fully methylated and unmethylated human DNA (Zymoresearch, USA) were used as positive and negative controls for the MSP-PCR.

### ChIP-qPCR

ChIP was performed to assess post-translational Histone H3 modifications (H3K27ac, H3K27me3 and H3K4me3) on the EPD annotated promoter, upstream 5’-UTR and exon 1 regions of the selected genes in MCF-7 cells under the influence of IL-1β (10 ng/mL) in the presence or absence of IL-1Ra (20 ng/mL) for 24 and 72 h, as previously described [55]. Briefly cells were crosslinked with 0.75% formaldehyde (Sigma-Aldrich, USA), quenched with glycine (BioRad, USA), lysed and chromatin was sheared by sonication (VCX750, Sonics, USA) to obtain fragments of 200 to 500 bp size. 5% of total sheared chromatin was reserved as input and immunoprecipitation performed overnight at 4 °C using antibodies listed in Supplementary Table S4 or normal-rabbit IgG (Merck Millipore, USA) (non-immune mock control), with Protein A/G plus agarose beads (Santa Cruz Biotechnology, USA). After washing, bound chromatin was eluted and reverse crosslinked and DNA purified using Wizard SV Gel and PCR Clean-Up System (Promega, USA). Purified immunoprecipitated DNA (~ 500 pg) was analyzed by qPCR using PowerUp SYBR Green Master Mix on a Quant Studio 6 Pro. Fold enrichment was calculated relative to mock control. Primer sequences and genomic regions analyzed are provided in Supplementary Table S5.

### Soft agar assay

MCF-7 breast cancer cells were suspended in DMEM-F12 (HiMedia, India) containing 0.4% low-melting-point agarose (Sigma-Aldrich, USA) with 10% FBS and layered over a solidified base of DMEM-F12 containing 0.8% agarose with 10% FBS. The embedded cells were treated with conditioned media (CM) collected after 72-hour treatments in the presence or absence of huE17 targeting IL-6R (100 ng/mL) for 21 days at 37 °C, 5% CO₂. Colonies were visualized under a bright-field inverted microscope at 10× magnification and quantified using ImageJ software.

### Statistical analysis

Statistical analyses were conducted using GraphPad Prism or R software. Data were expressed as mean ± standard deviation (SD) from at least two or more independent biological replicates. Appropriate parametric tests were applied as indicated in the figure legends. A p-value < 0.05 was considered statistically significant.

## Results

### Tumor-associated myeloid cells secrete IL-1β and IL-1 receptor complex expression correlates with poor prognosis in breast cancer patients

**Fig. 1:**
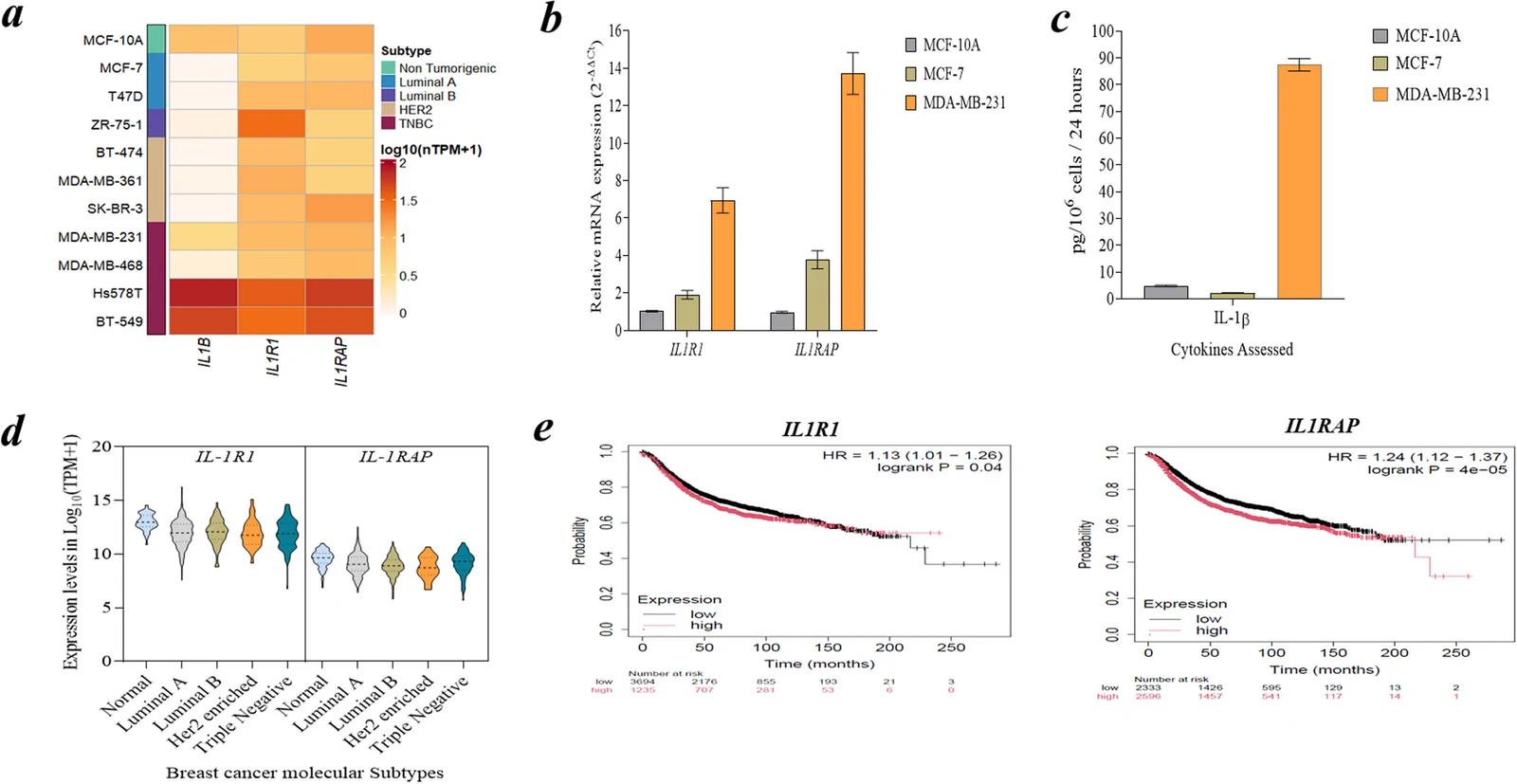
**a** Expression profiling of *IL1B*, *IL1R1* and *IL1RAP* in non-tumorigenic, luminal A, luminal B, HER2-enriched and Triple-negative breast cell lines. **b** qPCR validation of *IL-1R1* and *IL1RAP* expression in MCF-10 A, MCF-7 and MDA-MB-231 non-tumorigenic breast epithelial and cancer cell lines. **c** Quantification of IL-1β levels (pg/10^6^ cells/ 24 h) in MCF-10 A, MCF-7 and MDA-MB-231 non-tumorigenic breast epithelial and cancer cell lines by ELISA. **d** Expression profile of *IL1R1* and *IL1RAP* across molecular subtypes of TCGA breast cancer dataset. **e** Kaplan-Meier analysis of relapse free survival of breast cancer patients stratified by high and low *IL1R1* and *IL1RAP* expression

Single-cell transcriptomic analysis demonstrated that *IL1B* expression is predominantly restricted to myeloid lineage cells including monocytes, macrophages and dendritic cells within the breast TME (Supplementary Fig.S1), consistent with prior reports [9]. Analysis of the Human Protein Atlas cell line transcriptomic dataset revealed *IL1B* expression was minimal in luminal cell lines and elevated in triple negative breast cancer cell lines while the receptors *IL1R1* and *IL1RAP* were stably expressed across cell lines and highlights that breast cancer cell lines retain the capacity to respond to IL-1β (Fig. 1a). qPCR analysis validated expression of *IL-1R1* and *IL-1RAP* in non-tumorigenic breast cell line (MCF-10 A) as well as luminal (MCF-7) and triple negative (MDA-MB-231) breast cancer cell line models (Fig. 1b). Consistent with the HPA transcriptomic data, basal IL-1β secretion was significantly elevated in MDA-MB-231 cells compared with MCF-10 A and MCF-7 cells (Fig. 1c). Analysis of the TCGA breast cancer cohort demonstrated that *IL1R1* and *IL1RAP* were expressed across normal breast tissue and breast cancer molecular subtypes (Fig. 1d). Furthermore, the Kaplan-Meier survival analysis demonstrated that high *IL1R1* and *IL1RAP* (Fig. 1e) expression was significantly correlated with reduced relapse-free survival, suggesting that enhanced IL-1 signaling may contribute to poorer clinical outcomes. The negligible IL-1β secretion observed in MCF-7 cells suggests that IL-1β signalling in luminal breast cancer is predominantly mediated through a paracrine mode of action of myeloid derived IL-1β in luminal breast tumors, supporting use of MCF-7 as a model to study stromal cytokine-driven effects in-vitro.

We also evaluated the IL-6 signalling axis because of the well-established crosstalk between IL-1β and IL-6. scRNA-seq analysis demonstrated *IL6* expression was enriched in endothelial cells and cancer associated fibroblasts (Supplementary Fig. S2). qPCR analysis confirmed expression of *IL6R* and *IL6ST***(**GP130) in MCF-10 A, MCF-7 and MDA-MB-231 cells (Supplementary Fig.S3a), while basal IL-6 secretion was elevated in MDA-MB-231 cells compared with MCF-10 A and MCF-7 cells (Supplementary Fig. S3b) supporting the presence of an intact IL-6 signalling axis in the breast cancer models utilized. Protein expression of IL6R, GP130, IL1R1 and IL1RAP in MCF-10 A, MCF-7 and MDA-MB-231 cells was further validated by western blot analysis (Supplementary Fig. S4).

### IL-1β induces a sustained non-apoptotic G0/G1 arrest and cytokine induced senescence in luminal breast cancer cells

**Fig. 2:**
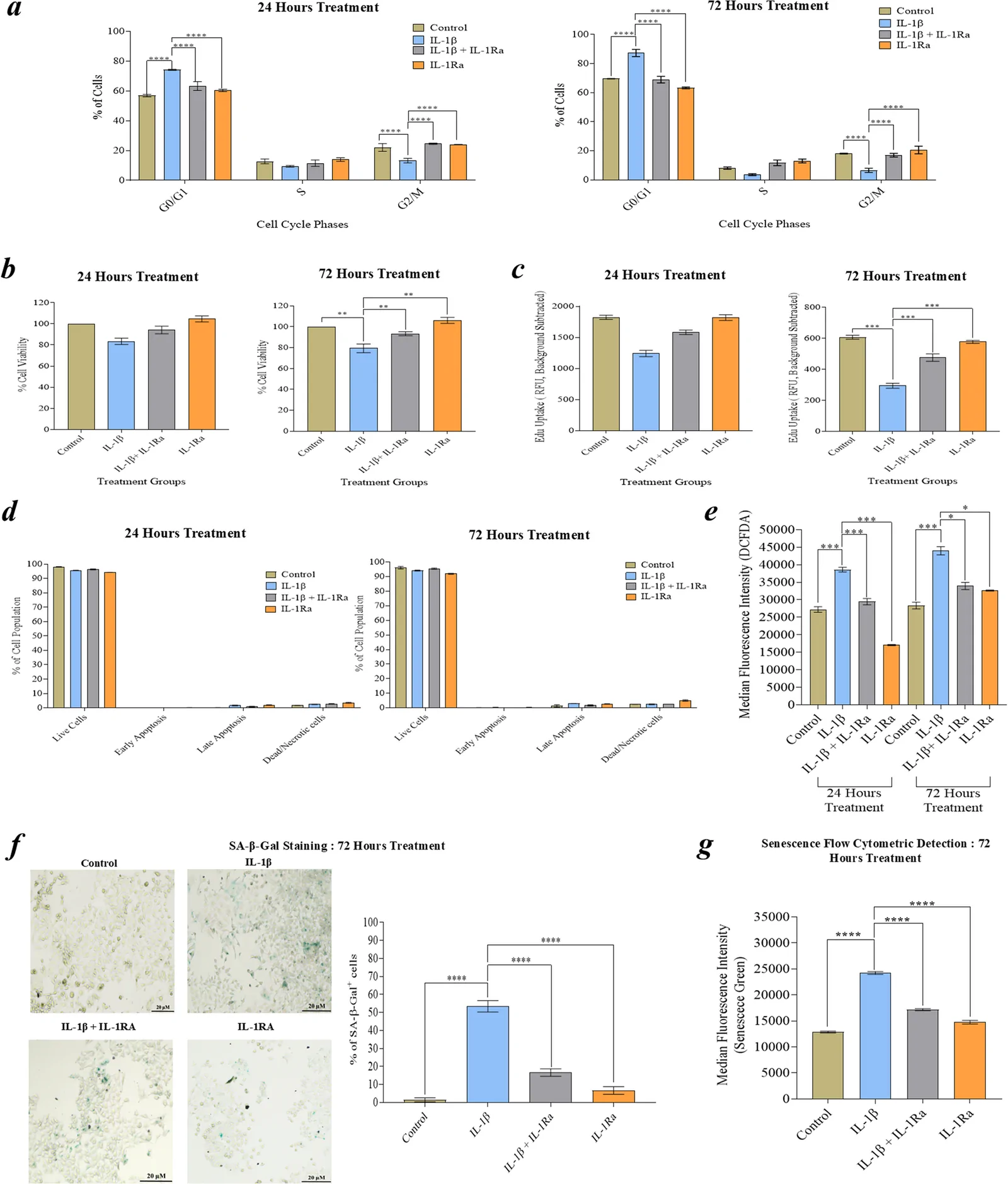
**a** Quantification of percentage of cells in G_0/_G_1_, S, and G2/M cell cycle phases under control, IL-1β, IL-1β + IL-1Ra, and IL-1Ra treatment conditions for 24 h and 72 h treatments in MCF-7 cells through flow cytometry. **b** CCK-8 assay results for control, IL-1β, IL-1β + IL-1Ra and IL-1Ra treatment conditions for 24 h and 72 h treatments in MCF-7 cells. **c** EdU incorporation assay in MCF-7 cells to measure DNA synthesis for control, IL-1β, IL-1β + IL-1Ra and IL-1Ra treatment conditions for 24 h and 72 h in MCF-7 cells. For statistical significance assessment Two-way ANOVA with Tukey’s multiple comparisons test was used for cell cycle analysis and ordinary one-way ANOVA with Tukey’s multiple comparisons test was used for CCK8 and EdU assays (*p < 0.05, **p < 0.01, ***p < 0.001, ****p < 0.0001). **d** Quantification of apoptotic stages post 24 h and 72 h treatments in MCF-7 cells for control, IL-1β, IL-1β + IL-1Ra and IL-1Ra treatment conditions. **e** Quantification of intracellular ROS measured as DCFDA mean fluorescence intensity (MFI) post IL-1β treatment for 24 and 72 h. One-way ANOVA with Tukey’s multiple comparisons test was used for significance testing (*p < 0.05, **p < 0.01, ***p < 0.001, ****p < 0.0001). **f** Representative images of SA-β-gal staining for control, IL-1β, IL-1β + IL-1Ra, and IL-1Ra treated MCF-7 cells post treatment for 72 h and quantification of senescence-associated β-galactosidase (SA-β-gal) positivity. At least 100 cells per field were manually counted across ten random fields (10×), and the percentage of SA-β-gal–positive cells was calculated as the mean across fields. Statistical significance was assessed using ordinary one-way ANOVA with Tukey’s multiple comparisons test (*p < 0.05, **p < 0.01, ***p < 0.001, ****p < 0.0001). **g** Flow cytometric analysis of cellular senescence using the CellEvent™ Senescence Green assay. Median fluorescence intensity (X-Med) of the gated Senescence Green-positive population in control, IL-1β, IL-1β + IL-1Ra, and IL-1Ra-treated MCF-7 cells following 72 h of treatment was used for measuring senescence. One-way ANOVA with Tukey’s multiple comparisons test was used for significance testing (*p < 0.05, **p < 0.01, ***p < 0.001, ****p < 0.0001)

Cell cycle analysis revealed that IL-1β induced growth arrest with a marked accumulation of cells in the G0/G1 phase following 24 h of treatment which persisted through 72 h, accompanied by corresponding reductions in S-phase entry (Fig. 2a). Consistent with cell cycle arrest, IL-1β reduced cell viability (Fig. 2b) and suppressed DNA synthesis as demonstrated by reduced EdU incorporation at both timepoints (Fig. 2c). IL-1Ra co-treatment partially reversed these effects, confirming that the observed growth arrest was dependent on IL-1β/IL-1R1/IL1RAP signaling. Despite sustained growth arrest, IL-1β did not promote apoptosis, as evident through no significant increase in apoptotic fractions at either timepoint (Fig. 2d). Instead, prolonged IL-1β exposure resulted in a significant increase in the proportion of SA-β-gal positive cells at 72 h and this was reversed by IL-1Ra treatment (Fig. 2f). Flow cytometric analysis further confirmed that IL-1β increased Senescence Green fluorescence in MCF-7 cells, while co-treatment with IL-1Ra attenuated this increase, consistent with the histochemical SA-β-gal findings (Fig. 2g). Furthermore, IL-1β significantly increased intracellular reactive oxygen species (ROS) levels at both 24 and 72 h, consistent with oxidative stress accompanying senescence induction (Fig. 2e). Representative cell cycle histograms, apoptosis quadrant plots, ROS and Senescence Green fluorescence histograms and scatter plots are provided in Supplementary Figs. S5-S8. Collectively, these findings demonstrate that IL-1β induces sustained G0/G1 arrest and promotes cytokine-induced senescence in luminal breast cancer cells.

### IL-1β induces a senescence-associated transcriptional program characterized by DNA damage signalling, apoptosis resistance and SASP induction

**Fig. 3:**
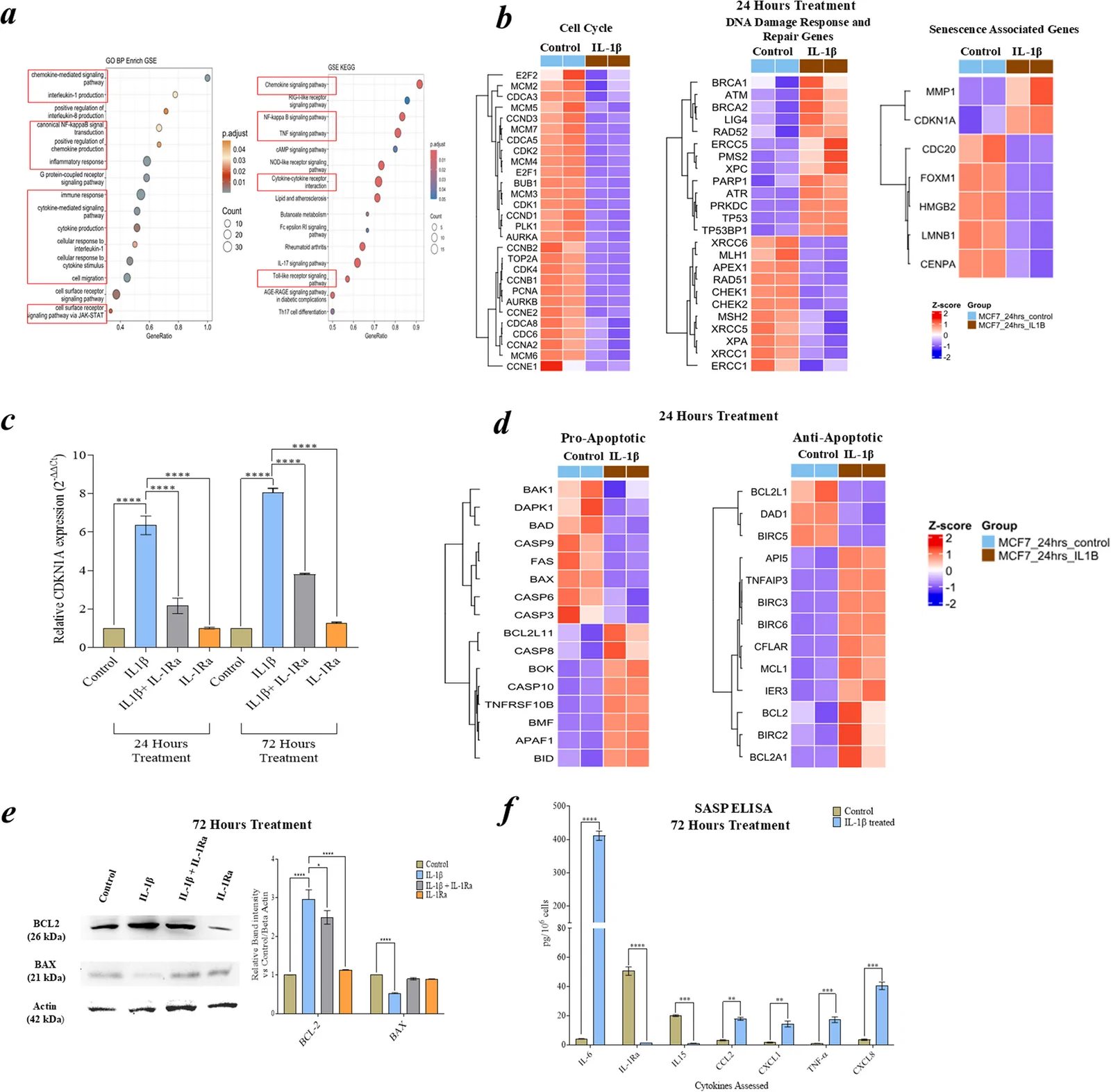
**a** Functional enrichment analysis of IL-1β induced transcriptional alterations in MCF-7 post 24-hour treatment using gene ontology and pathway prioritization tools gene ontology : biological processes and KEGG pathways. Adjusted *p*-value of < 0.05 (Benjamini-Hochberg correction) was considered statistically significant. **b** Heatmaps showing IL-1β induced transcriptomic modulation of cell cycle regulatory, DNA damage response and repair, and senescence associated genes in MCF-7 cells at 24 h IL-1β treatment. **c** qPCR quantification of *CDKN1A* (p21) mRNA expression after treatment with IL-1β in the presence/absence of IL-1Ra for 24 h and 72 h. One-way ANOVA with Dunnett’s multiple comparisons test was used to assess statistical significance (*p < 0.05, **p < 0.01, ***p < 0.001, ****p < 0.0001). **d** Heatmaps showing IL-1β induced transcriptomic modulation of pro-apoptotic and anti-apoptotic genes following IL-1β treatments in MCF-7 breast cancer cells for 24 h. **e** Western blotting and densitometric analysis for BCL2 and BAX in MCF-7 cells treated with IL-1β (10ng/ml) for 72 h period in presence or absence of IL-1Ra, the receptor antagonist of IL-1β (20ng/ml). Two-tailed unpaired t Test with Welch’s correction was applied to determine statistical significance (*p < 0.05, **p < 0.01, ***p < 0.001, ****p < 0.0001). **f** Luminex multiplex ELISA quantification analysis of secreted cytokines (IL-15, IL-1Ra, IL-6, CXCL8, TNF-α, CXCL1, and CCL2) in MCF-7 cells after 72 h of IL-1β treatment compared to untreated controls. One-way ANOVA with Dunnett’s multiple comparisons test was used to assess statistical significance (*p < 0.05, **p < 0.01, ***p < 0.001, ****p < 0.0001)

Functional enrichment analysis of RNA-seq data from MCF-7 cells treated with IL-1β for 24-hours demonstrated significant enrichment of biological processes associated with inflammatory cytokine and chemokine signaling through the NF-κB and STAT3 pathways (Fig. 3a). Representative Gene Ontology : molecular function, DisGeNET disease enrichment and MSigDB Hallmark analyses are provided in Supplementary Figs. S9-S10.

Transcriptomic profiling further revealed downregulation of cell cycle progression associated genes together with activation of DNA damage regulators, downregulation of DNA repair genes and senescence associated gene signatures following 24 h IL-1β treatment (Fig. 3b). Specifically, IL-1β treatment increased the expression of DNA damage signalling mediators including *ATM*, *ATR*, *TP53* and *TP53BP1*, while reducing the expression of DNA repair genes including *RAD51*, *XRCC1*, *APEX1*, *CHEK1* and *CHEK2*, indicating persistent activation of DNA damage signalling accompanied by impaired DNA repair capacity. Senescence-associated transcriptional changes included upregulation of *CDKN1A* (p21) and *MMP1*, together with reduced expression of proliferation-associated genes (*FOXM1*, *CDC20* and *CENPA*) and chromatin structural regulators *LMNB1* and *HMGB2*, consistent with acquisition of a cytokine-induced senescence phenotype. qPCR analysis confirmed IL-1β driven upregulation of *CDKN1A* (p21) expression at 24 and 72 h which was partially reversed by IL-1Ra co-treatment (Fig. 3c). Notably, concurrent upregulation *TP53* and *CDKN1A* (p21) is consistent with the activation of the canonical p53-p21 DNA damage response pathway, reported to be a key mediator of cytokine induced senescence [56, 57].

Transcriptomic analysis further demonstrated extensive suppression of apoptotic pathways. Core pro-apoptotic mediators of the intrinsic (mitochondrial) pathway (*BAX*, *BAK1*, *BAD*, *DAPK1* and *CASP9*), the extrinsic death-receptor pathway (*FAS*), and downstream executioner caspases (*CASP3* and *CASP6*) were downregulated while anti-apoptotic genes including *MCL1*,* BCL2*,* BCL2A1*,*CFLAR*,* BIRC2*,* BIRC3* and *BIRC6* were upregulated post IL-1β induction (Fig. 3d). These transcriptomic alterations were corroborated at the protein level, where IL-1β significantly increased BCL2 expression while reducing BAX protein levels following 72 h of treatment, effects that were partially reversed by IL-1Ra co-treatment (Fig. 3e). IL-1β induced increase in the BCL2 and reduction in BAX protein expression were also observed at the 24 h treatment timepoint and persisted post 72 h of cytokine washout indicating sustained modulation of apoptotic signalling (Supplementary Fig. S11). ELISA was also carried out to confirm successful cytokine washout, with only low residual levels of IL-1β detected in the culture medium one hour following media replacement (Supplementary Fig. S12).

Integration of the IL-1β transcriptome with the CellAge senescence database further demonstrated enrichment of established senescence-associated genes, including repression of several senescence inhibitors together with induction of senescence-promoting genes (Supplementary Figs. S13 and S14). Consistent with acquisition of a senescence-associated secretory phenotype (SASP), multiplex Luminex analysis demonstrated significantly increased secretion of IL-6, TNF-α, CXCL8, CXCL1 and CCL2, accompanied by reduced IL-15 and IL-1Ra levels following IL-1β treatment for 72 h (Fig. 3f). These observations were further supported by transcriptomic profiling of cytokine and chemokine expression at 24 and 48 h (Supplementary Figs. S15 and S16). Collectively, these findings demonstrate that IL-1β induces a sustained senescence-associated transcriptional program characterized by persistent DNA damage signalling, impaired DNA repair, apoptosis resistance and acquisition of a pro-inflammatory secretory phenotype.

### IL-1β promotes DNA methylation through DNMT induction and methylation-dependent repression of senescence-associated genes

**Fig. 4:**
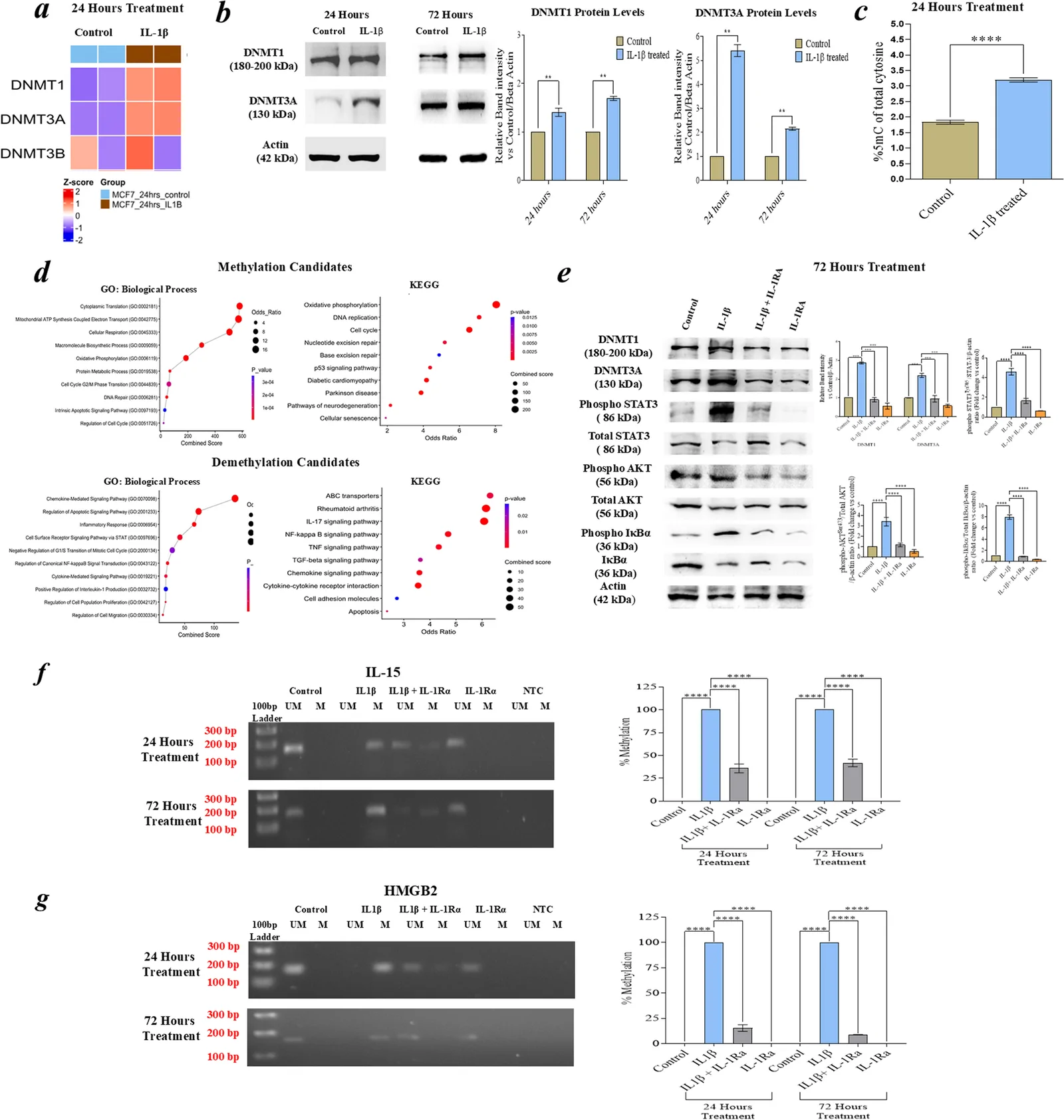
**a** Z-score normalized expression profiles of DNA methyltransferases (DNMTs) from the MCF-7 transcriptome dataset following IL-1β treatment for 24 h. **b** Assessment through western blotting and densitometric analysis of DNMT1 and DNMT3A protein levels post 24 h and 72 h of IL-1β (10 ng/ml) treatment of MCF-7 cells. Statistical significance was determined by two-tailed unpaired t Test with Welch’s correction (*p < 0.05, **p < 0.01, ***p < 0.001, ****p < 0.0001). **c** Quantification of global methylation by HPLC; the effect of IL-1β (10ng/ml) on global 5mc levels was assessed by comparing % 5-mC of total cytosine between controls and cytokine treated samples for MCF-7 cell lines for 24 h. One-way ANOVA was used to compare cytokine treated group against untreated control to assess statistical significance (*p < 0.05, **p < 0.01, ***p < 0.001, ****p < 0.0001). **d** Functional enrichment analysis of potential methylation candidate genes and demethylation candidate genes identified by integrating IL-1β induced transcriptomic changes with the NCI methylation profile for MCF-7 cells through gene ontology : biological processes and KEGG pathways. **e** Western blot and densitometric analysis for DNMT1, DNMT3A, pSTAT3 (Tyr705), total STAT3, pAKT (Ser473), total AKT, pIκBα (Ser32) and total IκBα in MCF-7 cells treated with IL-1β (10ng/ml) in presence or absence of IL-1Ra, the receptor antagonist of IL-1β (20ng/ml) for 72 h. Two-tailed unpaired t Test with Welch’s correction was applied to determine statistical significance (*p < 0.05, **p < 0.01, ***p < 0.001, ****p < 0.0001 ). **f** Representative methylation-specific PCR (MSP) gel images and Methylation % quantification (quantified from densitometric analysis of methylated and unmethylated MSP amplicons) for *IL-15* promoter in MCF7 cells treated for 24–72 h with control, IL-1β (10ng/ml), IL-1β + IL-1Ra (20ng/ml) and IL-1Ra alone. **g** Representative methylation-specific PCR (MSP) gel images and Methylation % quantification (quantified from densitometric analysis of methylated and unmethylated MSP amplicons) for *HMGB2* promoter in MCF7 cells treated for 24–72 h with control, IL-1β (10ng/ml), IL-1β + IL-1Ra (20ng/ml) and IL-1Ra alone. One-way ANOVA with Dunnett’s multiple comparisons test was used to assess statistical significance for MSP (*p < 0.05, **p < 0.01, ***p < 0.001, ****p < 0.0001)

In addition to identifying the establishment of a senescence associated transcriptional program, transcriptomic analysis also revealed upregulation of epigenetic regulators *DNMT1* and *DNMT3A* post IL-1β treatment for 24 h (Fig. 4a). Western blot analyses confirmed these findings at the protein level. We observed a statistically significant increase in DNMT1 (~ 1.5-fold) and DNMT3A (~ 5-fold) levels respectively at 24 h and 1.5-fold and 2-fold increase in DNMT1 and DNMT3A respectively at 72 h timepoint (Fig. 4b). Consistent with increased DNMT expression, HPLC-based global methylation analysis showed a statistically significant increase in global %5-mC levels following IL-1β exposure for 24-hours (Fig. 4c).

Further, integration of the IL-1β transcriptomic data with the NCI-60 baseline methylation profile for MCF-7 identified potential candidate genes that may be subject to promoter methylation associated repression or demethylation associated transcriptional activation (Fig. 4d). The putative methylation candidates were enriched for pathways related to cellular respiration, cell cycle regulation, senescence and apoptosis, whereas putative demethylation candidates were enriched for cytokine/chemokine production, inflammatory signaling, NF-κB and JAK/STAT3 signaling and epithelial-mesenchymal transition. Additional MSigDB Cancer Hallmark and DisGeNET analysis carried out for the methylation and demethylation candidates is provided in Supplementary Fig. S17. Notably we observed overlap between several of the putative methylation candidate genes and downregulated senescence-associated genes from the Cell Age senescence inhibitors list (Supplementary Fig. S18). This includes *HMGB2*, a key marker genes for senescence, thus suggesting that epigenetic silencing may contribute to maintenance of IL-1β induced senescence.

Since persistent inflammatory signalling can regulate DNMT expression, we next examined the activation status of major downstream signalling pathways. IL-1β significantly increased phosphorylation of STAT3, AKT and IκBα, together with elevated DNMT1 and DNMT3A protein expression following 72 h of treatment, whereas IL-1Ra co-treatment partially reversed these effects (Fig. 4e), demonstrating that IL-1β receptor signalling promotes activation of STAT3, PI3K/AKT and NF-κB pathways concurrent with induction of the DNA methylation machinery. The same patterns were also observed at the 24 h treatment time point as well as post cytokine washout (Supplementary Figs. S19 and S20).

To validate methylation associated candidate genes experimentally, Methylation specific PCR was performed for *IL15* and *HMGB2.* Assay specificity was confirmed through amplification using methylated and unmethylated human genomic DNA controls (Supplementary Fig. S21). IL-1β treatment increased promoter methylation of *IL15* and *HMGB2* at both 24 and 72 h, while IL-1Ra co-treatment partially reversed this effect (Fig. 4f and g). Correspondingly, *HMGB2* mRNA expression was reduced following IL-1β exposure and restored post IL-1Ra treatment (Supplementary Fig. S22). At the protein level, IL-1β decreased IL-15 secretion which was rescued by IL-1Ra treatment (Supplementary Fig. S23). These findings demonstrate that IL-1β promotes global and gene-specific DNA methylation accompanied by activation of inflammatory signalling pathways and DNMT induction.

### IL-1β induces altered expression of histone modifying enzymes and drives site-specific histone remodeling at inflammatory and apoptotic gene loci

Transcriptomic profiling post IL-1β treatment for 24 h further revealed altered expression of chromatin modifying enzymes including histone acetyl transferases (HATs), histone deacetylases (HDACs), histone methyltransferases (HMTs) and histone demethylases (HDMs) (Supplementary Fig.S24). At the gene level ChIP-qPCR demonstrated that IL-1β induced coordinated changes in activating and repressive histone marks across key inflammatory and apoptotic gene loci.

**Fig. 5:**
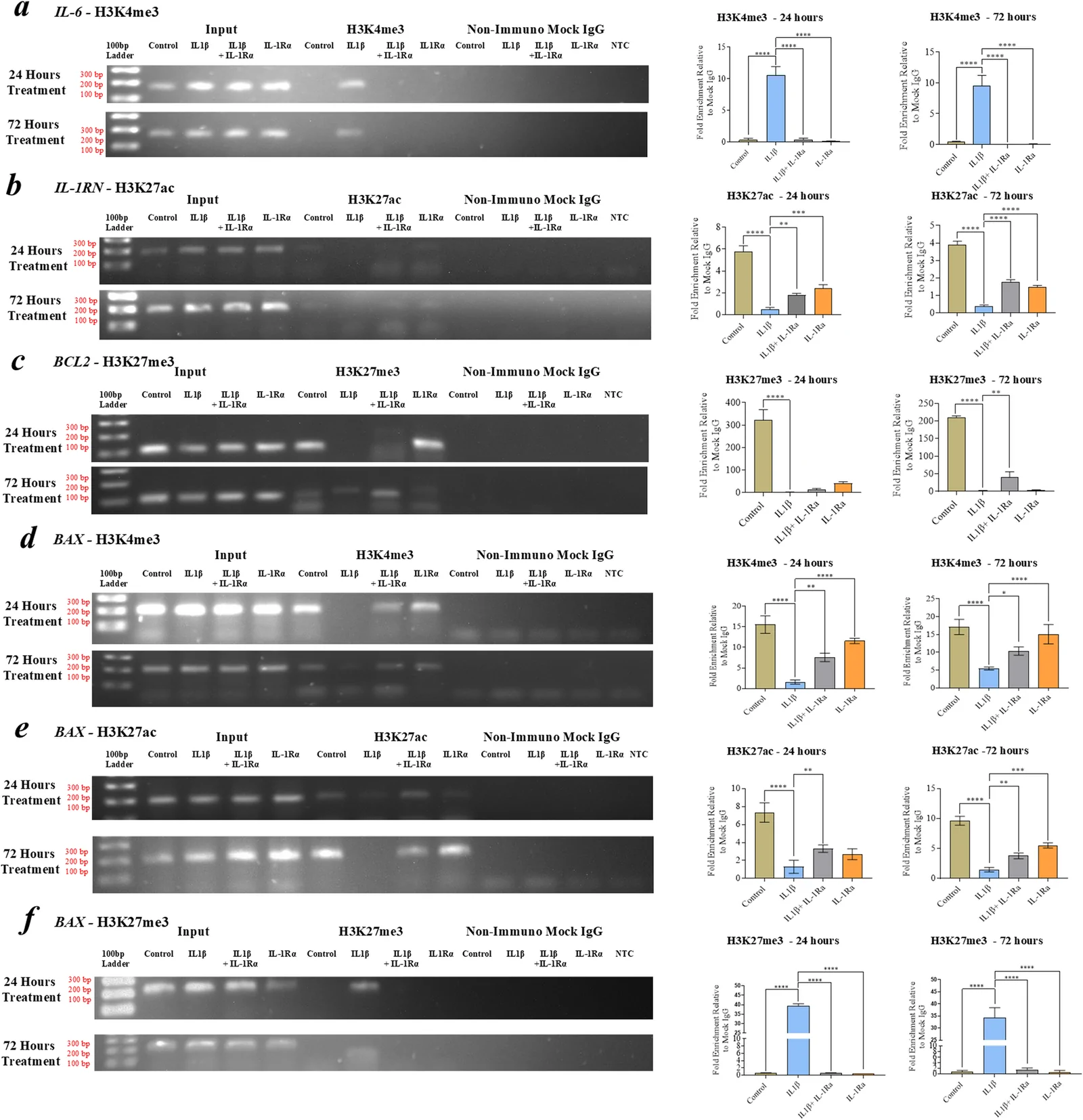
**a** Representative ChIP-qPCR gels showing H3K4me3 enrichment and accumulation at the *IL6* promoter and exon 1 after 24 and 72 h treatment with IL-1β (10ng/ml) in the presence/absence of IL-1Ra (20ng/ml) and corresponding fold-enrichment quantification. **b** Representative ChIP-qPCR gels depicting reduced H3K27ac enrichment at the *IL1RN* promoter and exon 1 in MCF7 cells after 24 and 72 h treatment with IL-1β (10ng/ml) in the presence/absence of IL-1Ra (20ng/ml) and corresponding fold-enrichment quantification. **c** Representative ChIP-qPCR gel images showing increased H3K27me3 enrichment across BCL2 in exon 1 after 24 and 72 h treatment with IL-1β (10ng/ml) in the presence/absence of IL-1Ra (20ng/ml) and corresponding fold-enrichment quantification. **d** Representative ChIP-qPCR gels showing reduced H3K4me3 enrichment upstream of the BAX promoter region after 24 and 72 h treatment with IL-1β (10ng/ml) in the presence/absence of IL-1Ra (20ng/ml) and corresponding fold-enrichment quantification. **e** Representative ChIP-qPCR gels showing reduced H3K27ac enrichment upstream of the BAX promoter region after 24 and 72 h treatment with IL-1β (10ng/ml) in the presence/absence of IL-1Ra (20ng/ml) and corresponding fold-enrichment quantification. **f** Representative ChIP-qPCR gels showing increased H3K27me3 enrichment upstream of the BAX promoter region after 24 and 72 h treatment with IL-1β (10ng/ml) in the presence/absence of IL-1Ra (20ng/ml) and corresponding fold-enrichment quantification. One-way ANOVA with Dunnett’s multiple comparisons test was used to assess statistical significance for ChIP-qPCR (*p < 0.05, **p < 0.01, ***p < 0.001, ****p < 0.0001)

At the *IL-6* locus, IL-1β treatment induced H3K4me3 enrichment at the promoter and first exon consistent with transcriptional activation (Fig. 5a), whereas IL-1β reduced H3K27ac occupancy in the promoter and first exon region of the *IL1RN* gene at 24 h and 72 h (Fig. 5b). Both changes were attenuated by IL-1Ra co-treatment and mirrored cytokine patterns observed via ELISA (Supplementary Fig.S23). At apoptosis-related loci, IL-1β decreased enrichment of repressive H3K27me3 marks at the *BCL2* first exon, at 24 and 72 h with partial reversal seen post IL-1Ra treatment (Fig. 5c). Conversely, at the pro-apoptotic *BAX* locus, IL-1β reduced activating H3K4me3 (Fig. 5d) and H3K27ac (Fig. 5e) histone mark enrichment upstream of the *BAX* promoter and induced repressive H3K27me3 histone mark occupancy at the *BAX* promoter and first exon (Fig. 5f). IL-1Ra treatment demonstrated reversal of the effects observed with respect to reduced occupancy of activating marks, but minimal effect was seen with regard to reversal of increased repressive H3K27me3 accumulation. These IL-1β induced histone mark remodeling patterns were in line with the changes observed in BCL2 and BAX protein levels (Fig. 3e and Supplementary Fig.S11). Collectively, these findings demonstrate that IL-1β drives coordinated epigenetic remodeling through locus-specific histone modifications, favoring activation of inflammatory signalling and anti-apoptotic genes while repressing pro-apoptotic and anti-inflammatory genes, thereby reinforcing the sustained cytokine-induced senescence phenotype.

### IL-6 reinforces STAT3-DNMT signaling axis downstream of IL-1β and contributes to maintenance of cytokine-induced senescence

**Fig. 6:**
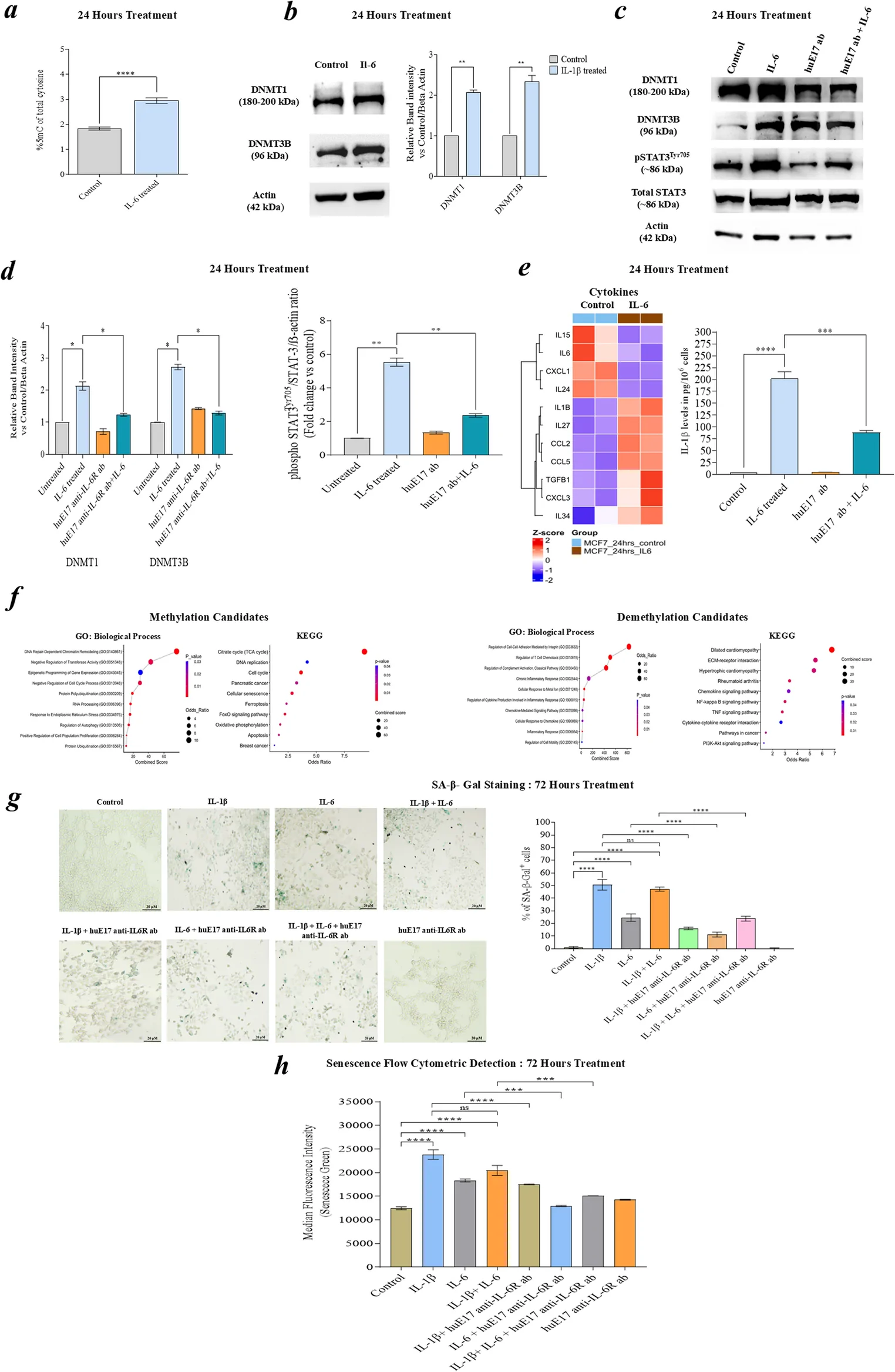
**a** Quantification of global DNA methylation by HPLC following IL-6 (25 ng/ml) treatment for 24-hours. %5-mC was calculated as a proportion of total cytosine. One-way ANOVA test was used to compare cytokine treated group against untreated control to assess statistical significance (*p < 0.05, **p < 0.01, ***p < 0.001, ****p < 0.0001). **b** Western blotting and densitometric analysis of DNMT1 and DNMT3B protein levels in MCF-7 cells post 24 h IL-6 (25 ng/ml) treatment. Statistical significance was determined by two-tailed unpaired t Test with Welch’s correction (*p < 0.05, **p < 0.01, ***p < 0.001, ****p < 0.0001). **c** Western blot and **d** Densitometric analysis of MCF-7 cells treated with IL-6 (25ng/ml) for 24 h in presence or absence of the huE17 anti-IL-6R humanized antibody (1μg/ml) and probed with antibodies for DNMT1, DNMT3B, total STAT3 and phospho-STAT3 (Tyr705). Phospho-STAT3 levels are represented as fold change of untreated control. Two-tailed unpaired t Test with Welch’s correction was applied to determine statistical significance (*p < 0.05, **p < 0.01, ***p < 0.001, ****p < 0.0001 ). **e** Expression profiling through transcriptomic analysis of cytokines and chemokine genes in IL-6 treated MCF-7 cells and ELISA validation of IL-6 induced IL-1β secretion and its inhibition by humanized anti-IL-6R (huE17) antibody. One-way ANOVA with Dunnett’s multiple comparisons test was used for statistical significance (*p < 0.05, **p < 0.01, ***p < 0.001, ****p < 0.0001). **f** Functional enrichment analysis of potential methylation and demethylation candidate genes identified by integrating IL-6 induced transcriptomic changes with the NCI methylation profile for MCF-7 cells through gene ontology : biological processes and KEGG pathways. **g** Representative SA-β-gal staining images of MCF-7 cells and quantification following 72 h treatment with IL-1β, IL-6, or their combination in the presence or absence of IL-6R blockade (huE17). At least 100 cells per field were manually counted across ten random fields (10×), and the percentage of SA-β-gal–positive cells was calculated as the mean across fields. Statistical significance was assessed using ordinary one-way ANOVA with Tukey’s multiple comparisons test (*p < 0.05, **p < 0.01, ***p < 0.001, ****p < 0.0001). **h** Flow cytometric analysis of cellular senescence using the CellEvent™ Senescence Green assay. Median fluorescence intensity (X-Med) of the gated Senescence Green-positive population in control, IL-1β, IL-6, IL-1β + IL-6, huE17, IL-1β + huE17, IL-6 + huE17, and IL-1β + IL-6 + huE17-treated MCF-7 cells following 72 h of treatment was used for measuring senescence. One-way ANOVA with Tukey’s multiple comparisons test was used for significance testing (*p < 0.05, **p < 0.01, ***p < 0.001, ****p < 0.0001)

Given that IL-1β induced marked upregulation of IL-6, the downstream effects of IL-6 induction were examined next. IL-6 induction (25 ng/mL) triggered a pronounced increase of global 5-mC levels (Fig. 6a) and a statistically significant rise in DNMT1 and DNMT3B levels at the protein (Fig. 6b) level after 24 h of treatment. Humanized huE17 anti-IL6R antibody, developed in-house [38] significantly suppressed IL-6 induced STAT3 phosphorylation and reduced DNMT1/3B expression (Fig. 6c-d). IL-6 stimulation was also associated with altered expression of multiple cytokines and chemokines in the transcriptomic analysis, including upregulation of IL-1β, which was validated at the protein level through ELISA (Fig. 6e). Spearman’s correlation between IL-1β and IL-6 expression in the TCGA-BRCA dataset (Supplementary Fig.S25) further supported this IL-1β/IL-6 inflammatory signaling loop observed in vitro through a modest but significant positive correlation in both the overall breast tumor cohort and within luminal A cases.

Functional integration of the IL-6 transcriptome with the NCI-60 baseline methylation data identified IL-6 responsive putative methylation and demethylation candidate genes (Fig. 6f). Putative methylation candidates were enriched for pathways associated with cell-cycle regulation and senescence (*CDC25A*,* PLK1*,* MCM2-4*), DNA damage response (*RAD1* and *UIMC1*), and apoptosis (*DAXX*). In contrast, putative demethylation candidates were enriched for cytokine and chemokine production (*CCL5*,* CCL21*,* CXCL13*,* IL1F10*) and NF-κB signalling (*TRAF3).* Additionally, MSigDB and DisGeNET analysis was also carried out for the methylation and demethylation candidates and is provided in Supplementary Fig. S26.

We further investigated the role of IL-6 in modulating IL-1β induced senescence, through SA-β-gal staining following treatment with IL-1β, IL-6, and co-treatment with both in the presence and absence of huE17. IL-6 treatment alone induced a modest increase in SA-β-gal positive cells compared to control. While co-treatment with IL-1β and IL-6 did not significantly enhance senescence beyond IL-1β alone, indicating a lack of synergistic effect. Notably, blockade of IL-6 signaling using huE17 significantly reduced SA-β-gal positivity in IL-1β-treated cells (Fig. 6g). Flow cytometric analysis showed a similar pattern, with IL-6 inducing a modest but lower increase in Senescence Green fluorescence compared to IL-1β, while co-treatment with IL-1β and IL-6 did not further increase fluorescence but maintained levels comparable to IL-1β alone. IL-6R blockade with huE17 reduced the fluorescence response, corroborating the pattern observed by histochemical SA-β-gal staining (Fig. 6h; Supplementary Fig. S27).These findings suggest that IL-6 is not a primary inducer of senescence but contributes to the maintenance of the IL-1β induced senescence state.

### The IL-1β/IL-6 inflammatory secretome promotes pro-tumorigenic phenotypes through IL-6 dependent signalling

**Fig. 7:**
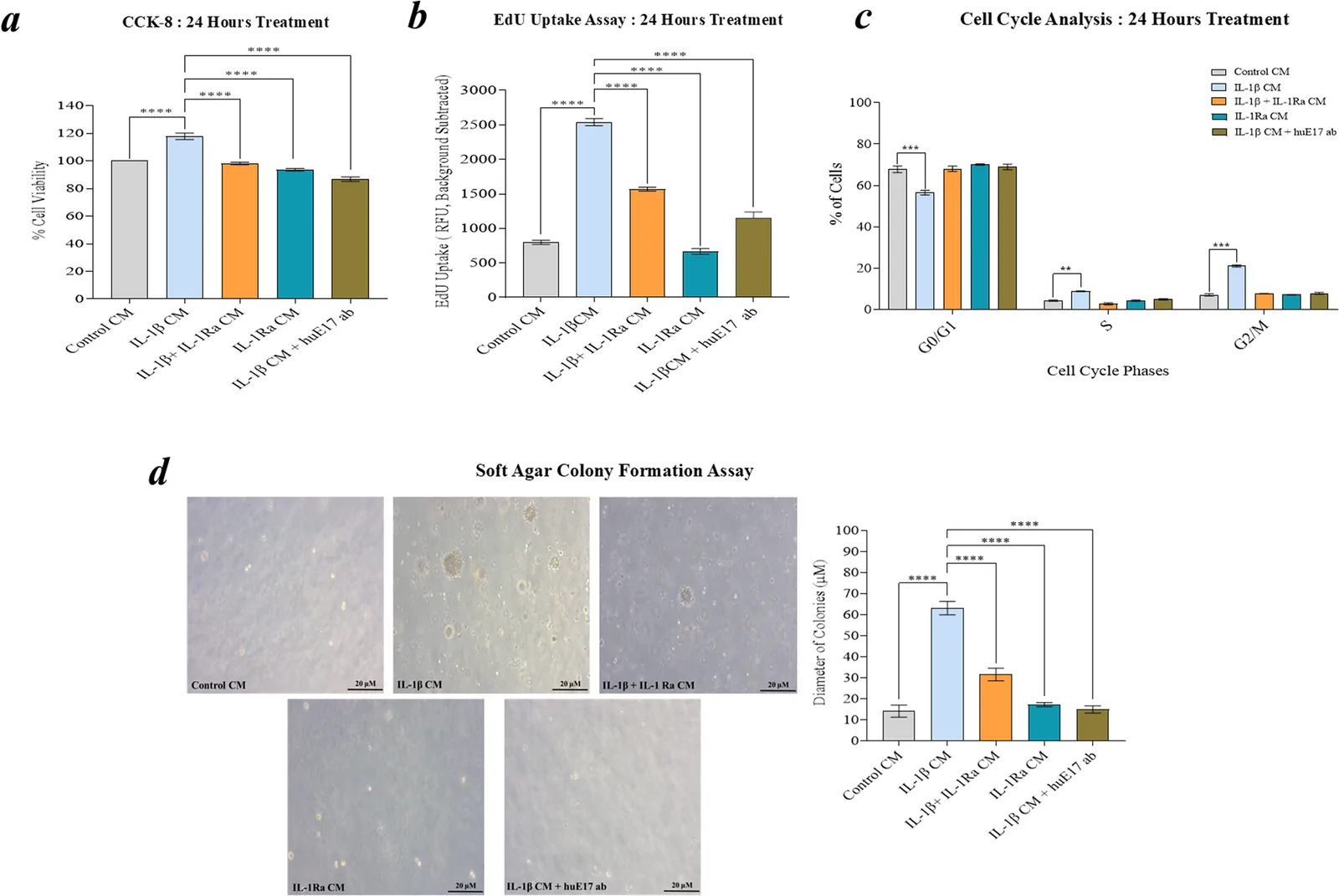
**a** CCK-8 and **b** EdU incorporation assays demonstrating increased proliferation and DNA synthesis following IL-1β conditioned media (CM) treatment, with partial reversal by IL-1Ra or IL-6R blockade. Ordinary one-way ANOVA with Tukey’s multiple comparisons test was used to assess significance (*p < 0.05, **p < 0.01, ***p < 0.001, ****p < 0.0001). **c** Quantification of percentage of cells in G_0/_G_1_, S, and G2/M cell cycle phases in naïve recipient MCF-7 cells following 24-hour treatment with conditioned media from the indicated treatment groups, including IL-1β-derived conditioned media with or without huE17 through flow cytometry. Two-way ANOVA with Tukey’s multiple comparisons test was used for cell cycle analysis to determine statistical significance. **d** Soft agar colony formation assay to assess anchorage-independent growth under IL-1β CM, attenuated by IL-1Ra or antibody treatment conditions and quantification of mean colony diameter. One-way ANOVA with Dunnett’s test was used for significance (*p < 0.05, **p < 0.01, ***p < 0.001, ****p < 0.0001)

To determine the functional consequences of the IL-1β induced secretory phenotype, conditioned medium collected from IL-1β treated MCF-7 cells was applied to naïve recipient MCF-7 cells. Conditioned media (CM) from IL-1β treated cells enhanced cell proliferation (Fig. 7a), drove DNA synthesis as evidenced through increased EdU incorporation (Fig. 7b) and significantly promoted cell cycle progression (Fig. 7c). These effects were partially attenuated by either IL-1Ra co-treatment or IL-6 receptor blockade using huE17, indicating that the proliferative effects of the conditioned medium are mediated through both IL-1β and IL-6 dependent signalling. Representative histograms for cell cycle analysis post conditioned media treatment is provided in Supplementary Fig. S28. IL-1β conditioned medium significantly increased anchorage-independent colony formation in soft agar (Fig. 7d). Neutralization of either IL-1β or IL-6 signalling markedly reduced colony growth, demonstrating that the tumour-promoting effects of the conditioned secretome depend on sustained inflammatory cytokine signalling.

Additionally, we examined whether the observed IL-1β responses with respect to epigenetic remodeling and senescence extended across breast non-tumorigenic epithelial and distinct breast cancer molecular subtype models, through comparative analyses performed in MCF10A and MDA-MB-231 cells. IL-1β treatment produced minimal changes in global DNA methylation and DNMT expression in non-tumorigenic MCF10A cells, whereas increased global DNA methylation and DNMT expression were observed in MDA-MB-231 cells. Comparative CellAge transcriptomic analysis of MDA-MB-231 cells demonstrated only limited overlap between IL-1β regulated genes and established senescence-associated gene signatures following 72 h of IL-1β treatment. These findings indicate that while IL-1β induced epigenetic alterations across multiple breast cancer models, the downstream transcriptional response differs between luminal and triple-negative breast cancer cells (Supplementary Fig. S29-S32). Collectively, our findings support a model in which myeloid-derived IL-1β initiates inflammatory epigenetic and transcriptional reprogramming in luminal breast cancer cells, establishing an IL-1β/IL-6 feed-forward signalling axis that maintains cytokine-induced senescence while simultaneously generating a tumour-promoting secretory microenvironment.

## Discussion

Chronic inflammation is a critical driver of breast cancer progression, yet the coordinated role of cytokine-mediated inflammatory signaling in shaping epigenetic signatures and tumor progression remains incompletely understood. The present study identified an IL-1β/IL-6 cytokine circuit that links inflammation, cytokine induced senescence, and epigenetic reprogramming associated with tumor progression in luminal breast cancer cells. We demonstrate that IL-1β initiates a cytokine induced senescence program characterized by a pro-inflammatory and pro-survival phenotype, with IL-6 acting downstream to reinforce STAT3/DNMT signaling, sustain IL-1β induced senescence and promote tumor supporting phenotypes.

IL-1β treatment triggered robust G0/G1 arrest without inducing apoptosis, accompanied by repression of multiple DNA repair genes and ROS accumulation. This combination of cell cycle arrest, oxidative stress, impaired DNA repair signaling, elevated SA-β-gal activity - further corroborated by flow cytometric analysis and absence of apoptosis is consistent with a cytokine induced senescence state, wherein growth arrest co-exists with sustained metabolic and secretory activity. Cytokine induced senescence is frequently mediated through activation of the p53-p21 axis [57] and this is in agreement with our findings. Notably cytokine induced senescence in cancer cells has been reported to occur through a *CDKN2A* (p16) independent senescence program [58, 59]. This is particularly relevant in luminal breast cancers, where CDKN2A is frequently functionally lost through promoter methylation or gene deletion [68,69]. Transcriptomic analyses further revealed downregulation of senescence inhibitors and upregulation of senescence promoting genes, alongside activation of canonical inflammatory pathways including NF-κB, PI3K/AKT and STAT3. Integration with the CellAge database further independently supported enrichment of established senescence-associated gene signatures. Prior studies have shown inflammatory cytokines including IL-1β and IL-6 can induce or reinforce cytokine induced senescence through oxidative stress, DNA damage signaling, and NF-κB dependent inflammatory pathways [22, 24]. Similarly, other studies have also reported IL-1β induced senescence in colorectal and lung cancers through activation of inflammatory signalling pathways and the senescence-associated secretory phenotype (SASP) [28, 60]. Our findings are consistent with these reports and further support IL-1β as an important mediator of inflammation-associated senescence in luminal breast cancer models. Beyond corroborating previous observations, our study demonstrates that IL-1β induced senescence is accompanied by coordinated epigenetic remodeling and establishment of a tumour-promoting inflammatory secretory phenotype and provides mechanistic insight into how chronic inflammatory signalling contributes to tumour-supportive phenotypes.

These findings have significant biological implications and support a model in which chronic inflammatory cytokine signalling functions not merely a transient response to cellular stress but can also induce stable phenotypic reprogramming in tumour cells through coordinated epigenetic remodeling. By coupling cytokine-induced senescence with epigenetic remodeling and establishment of a tumour-promoting secretory phenotype, IL-1β may facilitate long-term maintenance of a pro-inflammatory tumour-supportive microenvironment. This provides a potential mechanistic explanation for how persistent inflammation contributes to tumour progression beyond its immediate effects by means of altering epigenetic signatures.

Epigenetic regulation of inflammatory pathways through DNA methylation and histone acetylation/deacetylation has been well documented, including dynamic methylation control of the IL-1β promoter itself during inflammatory activation [61]. In contrast, our findings highlight the reciprocal dimension of this relationship, demonstrating that inflammatory cytokine signaling itself, particularly IL-1β, can actively reshape the epigenetic landscape in tumor cells.

A key observation in our study was that IL-1β induced rapid epigenetic reprogramming. We observed upregulation of DNMT1 and DNMT3A at transcript and protein level together with a significant increase in global 5-mC levels, indicating that IL-1β can modulate DNA methylation dynamics. In parallel, altered expression patterns of histone modifying proteins - HATs, HDACs, HMTs and HDMs suggested broader chromatin remodeling events. These global shifts were functionally validated at the gene level through MSP and ChIP-qPCR with IL-1β inducing promoter specific methylation and rearrangement of histone marks in 5’-UTR, promoter and first exon regions of inflammatory, survival and SASP-related gene loci. IL-1β mediated methylation has also been previously reported in breast cancer through TWIST1 mediated ESR1 promoter methylation [62]. Our findings expand this paradigm by demonstrating coordinated epigenetic remodeling across multiple inflammatory and survival-associated loci.

Among the genes affected, IL-15 and HMGB2 are of particular interest. IL-15 is critical for recruitment and activation of NK cells that mediate clearance of senescent tumor cells and its silencing may facilitate persistence of senescent tumour populations [70]. HMGB2 is a chromatin architectural protein [63] whose downregulation during senescence is associated with chromatin compaction and stabilization of growth arrest through formation of senescence associated heterochromatin foci (SAHF) [64]. Thus, repression of IL15 and HMGB2 by IL-1β driven promoter DNA methylation may contribute to maintenance of a cytokine induced senescence tumor-supportive state.

Histone mark remodeling further supported this model. IL-1β induced enrichment of activating H3K4me3 marks at the *IL-6* locus and loss of repressive H3K27me3 at *BCL2* locus, while simultaneously promoting accumulation of repressive H3K27me3 and loss of activating marks at the *BAX* and *IL1RN* promoters. The concurrent upregulation of BCL-2 and IL-6, along with downregulation of IL-1RN and BAX, would favor sustained NF-κB/STAT3 activation driven inflammatory survival and anti-apoptotic programs. Consistent with these transcriptional changes, IL-1β-treated cells displayed a pro-inflammatory and pro-tumorigenic SASP characterized by upregulation of TNF-α, CXCL1, CXCL8 and CCL2 alongside suppression of IL-15 and IL-1Ra. This may promote tumor progression through paracrine effects on neighboring cells. Indeed, conditioned media from IL-1β treated cells enhanced proliferation, DNA synthesis, and anchorage-independent growth in naïve MCF-7 cells, confirming that IL-1β generates a tumor-promoting secretome.

Given the strong induction of IL-6 as part of the IL-1β-driven SASP, our findings further demonstrate that IL-6 acts downstream of IL-1β, inducing DNMT1 and DNMT3B, activating STAT3 signaling and upregulating IL-1β, forming an inflammatory cytokine amplification loop. This relationship was further supported by the significant positive correlation between *IL-1β* and *IL-6* expression in the TCGA-BRCA datasets. Blockade of IL-6 signaling abrogated STAT3 phosphorylation, reduced DNMT1/3B levels and reversed the proliferative and anchorage independent growth effects of IL-1β conditioned media. Importantly, our findings indicate that IL-6 reinforces and sustains the senescence phenotype rather than initiating it, as IL-6 alone induced only a modest senescence response and did not synergistically enhance IL-1β induced senescence, whereas IL-6R blockade attenuated IL-1β-driven SA-β-gal activity, a pattern corroborated by flow cytometric analysis. These findings position IL-6 as a key mediator linking IL-1β induced senescence and tumor-promoting phenotypes.

While the present study provides important biological insights, several limitations should be acknowledged. The mechanistic investigations were primarily centred on the luminal MCF7 model, however comparative analyses performed in MCF10A and MDA-MB-231 cells demonstrated distinct subtype-specific responses to IL-1β, which supported the selection of MCF7 as the primary mechanistic model while also emphasizing the context-dependent nature of IL-1β signalling and its downstream consequences across breast cancer subtypes. These findings further highlighted the need for future investigations. Furthermore, the epigenetic analyses focused primarily on global DNA methylation together with targeted DNA methylation and histone modifications at selected loci. Future studies incorporating genome-wide methylome and chromatin accessibility profiling together with validation in additional luminal breast cancer models, patient-derived organoids and in vivo systems will be valuable for defining the broader biological and clinical significance of the IL-1β driven inflammatory-epigenetic axis.

**Fig. 8:**
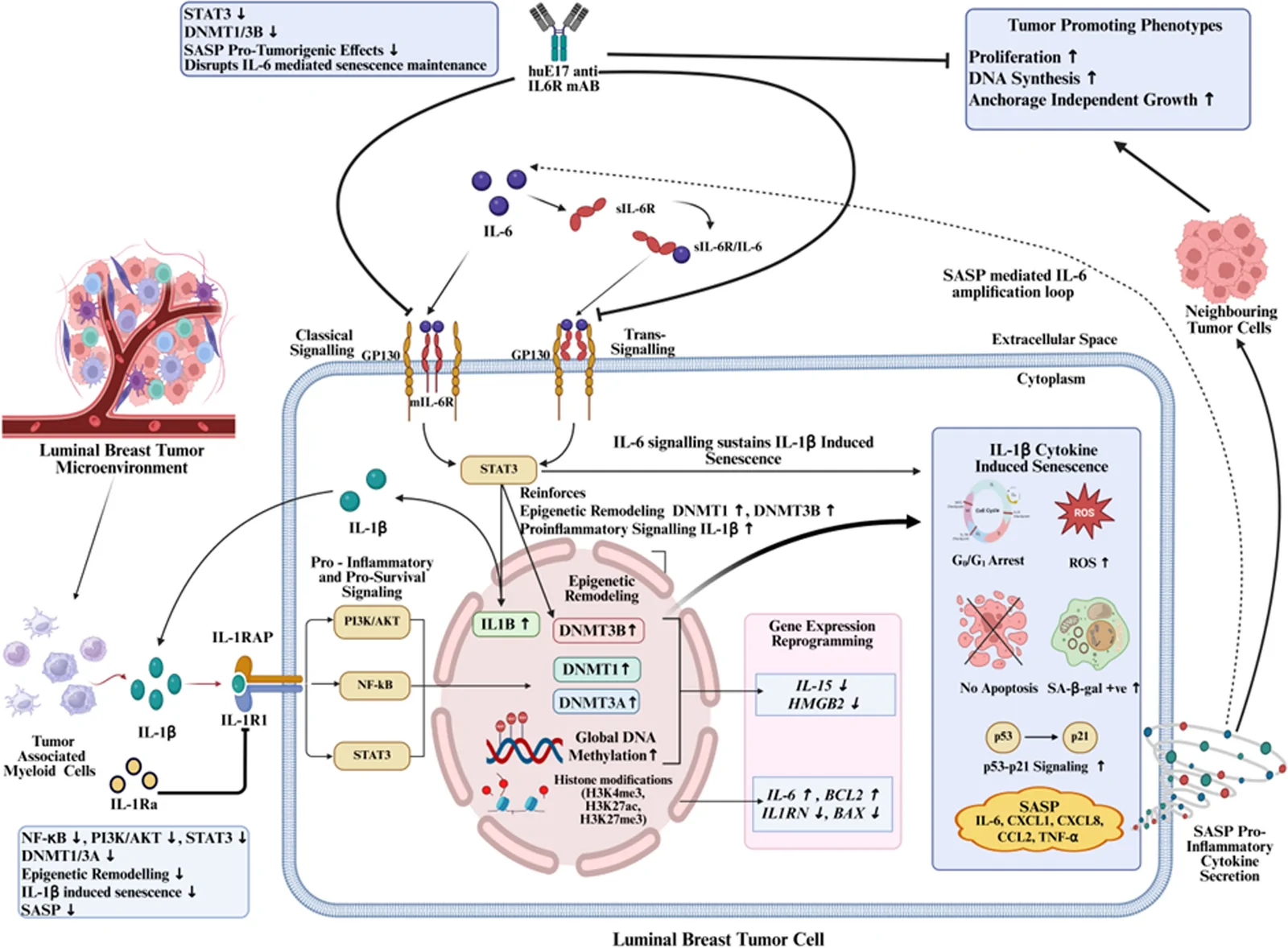
Schematic representation of the IL-1β/IL-6 inflammatory-epigenetic-senescence axis in luminal breast cancer. IL-1β derived from tumor associated myeloid cells acts through the IL-1R1/IL-1RAP complex to activate NF-κB, PI3K/AKT and STAT3 signaling cascades, inducing pro-inflammatory and pro-survival signaling. This is accompanied by epigenetic remodeling, including increased DNMT1 and DNMT3A expression, elevated global DNA methylation and altered histone modifications, resulting in transcriptional reprogramming (*IL-6* and *BCL2* upregulation; *IL1RN*, *BAX*, *IL-15* and *HMGB2* downregulation). These changes establish an IL-1β driven Cytokine Induced Senescence phenotype characterized by G0/G1 arrest, increased ROS, p53–p21 activation, SA-β-gal positivity and absence of apoptosis. Senescent cells acquire a SASP marked by secretion of IL-6, CXCL1, CXCL8, CCL2 and TNF-α. SASP-derived IL-6 signals via IL-6R/GP130 to activate STAT3, reinforcing epigenetic remodeling through increased DNMT3B expression and sustaining IL-1β Induced Senescence, thereby forming a cytokine amplification loop. SASP factors act in a paracrine manner to promote proliferation, DNA synthesis and anchorage-independent growth in neighboring tumor cells. Blockade of IL-6 signaling using anti-IL-6R antibody (huE17) disrupts this loop and attenuates downstream signaling and tumor-promoting effects

Taken together, this study identifies an inflammatory-epigenetic-senescence axis in luminal breast cancer driven by a self-reinforcing inflammatory IL-1β/IL-6 cytokine loop. IL-1β initiates cytokine induced senescence, SASP activation and broad epigenetic remodeling, while IL-6 reinforces STAT3/DNMT-linked epigenetic changes and sustains cytokine induced senescence and chronic inflammatory signaling. Functionally, the resulting secretome enhances proliferation and anchorage independent growth in naïve tumor cells. Our findings demonstrate that inflammatory cytokines do not merely activate transient transcriptional inflammatory programs but can also actively remodel the epigenetic landscape of tumor cells, linking inflammatory signaling directly to chromatin remodeling and senescence-associated phenotypes (Fig. 8).

These findings underscore the need to further dissect how cytokine driven inflammatory signaling interfaces with epigenetic remodeling within the tumor microenvironment to shape tumor progression. Importantly, increasing evidence suggests that chronic IL-1β signalling contributes to therapeutic resistance and tumour recurrence in multiple malignancies through maintenance of senescent, polyploid tumour cell populations and promotion of senescence-associated cellular reprogramming in non-small cell lung cancers and colorectal cancers [28, 60]. More recently, IL-1RAP expressing myeloid cells have emerged as an important regulator of chemoresistance and immune suppression, with antibody-mediated blockade improving chemoimmunotherapy responses in pancreatic cancers [65]. Although these observations remain to be validated in luminal breast cancer, our findings identify IL-1β as an upstream inflammatory regulator linking chronic inflammation, cytokine-induced senescence and epigenetic remodeling, providing a strong biological rationale for investigating therapeutic strategies combining IL-1β/IL-6 pathway inhibition with epigenetic modulators or senolytics. The efficacy of IL-6 pathway blockade observed in the present study further supports the feasibility of this therapeutic strategy. Such combination approaches may prevent establishment of stable tumour-supportive cellular states while simultaneously targeting persistent inflammatory signalling and senescent tumour cell populations, thereby representing a promising therapeutic strategy to improve therapeutic responses and reduce disease recurrence in case of breast cancer.

## Supplementary Information

Below is the link to the electronic supplementary material.

Supplementary Material 1.

Supplementary Material 2.

Supplementary Material 3.

## Data Availability

All data supporting the conclusions of the study are represented fully in the article and supplementary files. Additional data are available from the corresponding authors upon request.
