# Supplementary material for: IL-1β/IL-6 inflammatory crosstalk drives DNA methylation and histone remodeling associated with cytokine induced senescence in luminal breast cancer
Source: Inflamm Res. 2026 Oct 17;75(1):246. doi: 10.1007/s00011-026-02391-7 (PMC13676678; doi:10.1007/s00011-026-02391-7)
Supplement: Supplementary file 1 [file 11_2026_2391_MOESM1_ESM.pdf]

IL-1β/IL-6 Inflammatory Crosstalk Drives DNA Methylation and Histone Remodeling Associated with Cytokine Induced Senescence in Luminal Breast Cancer

Satyajit Dey Pereira[1*], Kanaka Sai Ram Padam[1,a], Padival Shruptha[2], Aditi Atreya[1], Manjunath B. Joshi[3], Kapaettu Satyamoorthy[2*]

[1]Department of Cellular and Molecular Biology, Manipal School of Life Sciences, Manipal Academy of Higher Education, Manipal-576104, India

[2]SDM Centre for Cellular and Molecular Sciences, SDM College of Medical Sciences and Hospital, Shri Dharmasthala Manjunatheshwara (SDM) University, Dharwad, India

[3]Department of Ageing Research, Manipal School of Life Sciences, Manipal Academy of Higher Education, Manipal-576104, India

[a]Present address: Herman B Wells Center for Pediatric Research, Department of Pediatrics, Indiana University School of Medicine, Indianapolis, IN 46202, USA

* Corresponding authors

Satyajit Dey Pereira
Department of Cell and Molecular Biology
Manipal School of Life Sciences
Manipal Academy of Higher Education
Manipal, India
Email : Satyajit.pereira@learner.manipal.edu
Satyajitdp12@gmail.com

Dr. Kapaettu Satyamoorthy
SDM College of Medical Sciences and Hospital,
Sri Dharmasthala Manjunatheshwara (SDM) University,
Manjushree Nagar, Sattur,
Dharwad, Karnataka, India-580009
Email: ksatyamoorthy@sdmuniversity.edu.in
ksatyamoorthy@yahoo.com

Supplementary Table 1 : qPCR Primer details

Primer Name	Primer Sequence	Tm (℃)	Amplicon Size
IL1-R1 FP	GGCTGAAAAGCATAGAGGGAAC	61.0 ℃	137 bp
IL1-R1 RP	CTGGGCTCACAATCACAGG	60.1 ℃	
IL-1RAP FP	ACACTTCTGTGGTGTGTAGTGA	60.9 ℃	91 bp
IL-1RAP RP	TGGTGTCTAGTCCCCAGTCAT	61.7 ℃	
IL-6R FP	CCCTCAGCAATGTTGTTTG	61.8 ℃	170 bp
IL-6R RP	CTCCGGGACTGCTAACTG	61.0 ℃	
GP130 FP	GAAGCAAGTGGGATCACCTA	61.5 ℃	88 bp
GP130 RP	TGTAGCCTTGAGTATGGGATG	61.7 ℃	
HMGB2 FP	CTCGCTATGACAGGGAGA	59.0 ℃	124 bp
HMGB2 RP	CGATGTTCAGAGCAAAACA	60.0 ℃	
CDKN1A FP	AGGTGGACCTGGAGACTCTC	62.4 ℃	95 bp
CDKN1A RP	CCTCTTGGAGAAGATCAGCC	62.9 ℃	
ACTB FP	GCACCACACCTTCTACAATG	60.1 ℃	131 bp
ACTB RP	GGGTGTTGAAGGTCTCAAA	60.7 ℃	

Supplementary Table 2 : Antibodies for Western blot

Protein	Host Species	Catalog number and company	Dilution
Actin	Mouse (monoclonal)	MA5-15739 (Invitrogen)	1 : 8000
IL-6R	Mouse (monoclonal)	AF1830 (R&D Biosystems)	1: 5000
GP130	Goat (Polyclonal)	AF-228-NA (R&D Biosystems)	1: 5000
IL-1R1	Rabbit (monoclonal)	A5725 (Abclonal)	1:2500
IL-1RAP	Rabbit (monoclonal)	A22286 (Abclonal)	1:2500
BCL2	Rabbit (monoclonal)	A19693 (Abclonal)	1:2500
BAX	Rabbit (monoclonal)	A19684 (Abclonal)	1:2500
DNMT1	Rabbit (monoclonal)	5032S (CST)	1: 4000
DNMT3A	Rabbit (monoclonal)	3598S (CST)	1: 4000
DNMT3B	Rabbit (monoclonal)	57868S (CST)	1: 4000
Phospho-STAT3 (Tyr705)	Rabbit (monoclonal)	9145S (CST)	1: 5000
Total STAT3	Rabbit (monoclonal)	12640S (CST)	1: 5000
Anti-AKT1 (pSer473) + AKT2 (pSer474) + AKT3 (pSer472)	Rabbit (monoclonal)	ab192623 (Abcam)	1: 5000
Anti-AKT1 + AKT2 + AKT3	Rabbit (monoclonal)	ab185633 (Abcam)	1: 5000
Phospho-IκBα (Ser32)	Rabbit (monoclonal)	AP0707 (Abclonal)	1:2500

Total IκBα	Rabbit (monoclonal)	A19714 (Abclonal)	1:2500
Rabbit Anti-Goat IgG H&L (HRP)	Rabbit (polyclonal)	Ab6741	1:10000
Goat Anti-Rabbit IgG H&L (HRP)	Goat (polyclonal)	Ab6721 (Abcam)	1:10000
Goat Anti-Mouse IgG H&L (HRP)	Goat (Polyclonal)	Ab6789 (Abcam)	1:10000

Supplementary Table 3 : MSP primer sequences (*IL15, HMGB2*)

**Primer Name**	**Primer Sequence**	**Tm (°C)**	**Amplicon Size**	**Description**
IL15_MSP_M_FP	TGGGGTAGTTAGTTATTTAGGAGTC	62 °C	162 bp	The targeted region in case encompasses 11 CpG sites, the EPD annotated *IL-15* promoter, the transcription start site(TSS) and was located within the CpG island associated with the *IL-15* promoter.
IL15_MSP_M_RP	ATCAACTAAAATTCCCCCGAA	62 °C		
IL15_MSP_UM_FP	TGGGGTAGTTAGTTATTTAGGAGTTGT	62 °C	163 bp	
IL15_MSP_UM_RP	AATCAACTAAAATTCCCCCAAA	62 °C		
HMGB2_MSP_ME_FP	CGTGGTTTAGTTCGTTAAGT	58 °C	176 bp	In case of *HMGB2* the targeted region spanned 178 bp, encompassing 14 CpG sites, the EPD annotated *HMGB2* promoter, the transcription start site(TSS) and was within the *HMGB2* promoter associated CpG island.
HMGB2_MSP_ME_RP	AAAAAATATTCTTACCTAATACGAA	58 °C		
HMGB2_MSP_UM_FP	TGGTTTAGTTTGTTAAGTTGTTG	60 °C	178 bp	
HMGB2_MSP_UM_RP	AAAAAATATTCTTACCTAATACAAA	60 °C		

Supplementary Table 4 : ChIP Antibodies

Histone Mark	Host Species	Catalog number and company
H3K4me3	Rabbit (monoclonal)	9751S (CST)
H3K27ac	Rabbit (monoclonal)	8173S (CST)
H3K27me3	Rabbit (monoclonal)	9733S (CST)

Supplementary Table 5 : ChIP-qPCR primer sequences and targeted regions (*IL-6, IL1RN, BAX, BCL2*)

Primer Name	Primer Sequence	Amplicon Size	Description
IL6_CHIP_A_FP	GTCTTGCCATGCTAAAGGAC	178 bp	Covers the 5'UTR, and part of the core promoter that are reported to have enrichment of H3K4me1 and H3k27me3 marks
IL6_CHIP_A_RP	CCTCAGACATCTCCAGTCCTAT		
IL6_CHIP_B_FP	ATTAGAGTCTCAACCCCCAAT	165 bp	Covers the remaining portion of the core promoter region and the downstream first exon region
IL6_CHIP_B_RP	GCAGTTCCAGGGCTAAGG		
IL1RN_CHIP_A_FP	AGGGGAGGGAATCAGTTACA	180 bp	Covers 5' UTR and core promoter region
IL1RN_CHIP_A_RP	GGCCCAGAGTTGTGGTTTAT		
IL1RN_CHIP_B_FP	ATAAACCACAACTCTGGGCC	192 bp	Covers remaining part of core promoter region and downstream exonic region with Histone enrichments
IL1RN_CHIP_B_RP	CCTCCTTGGGGTAGCCTT		

BAX_CHIP_A__FP	CGGTAGCTCATGCCTGTAAT	200 bp	Covers part of the H3K27ac enriched region upstream of the promoter
BAX_CHIP_A__RP	AGTGCAAAAGCTCAGAGGC		
BAX_CHIP_B__FP	GCCTCTGAGCTTTTGCAC	176 bp	Covers remaining portion of the H3K27ac enriched region and a portion of the barren region for MCF-7 but which is H3K4me3 enriched in other cell lines
BAX_CHIP_B__RP	GTGACTGTCCAATGAGCATC		
BAX_CHIP_C__FP	GTGACGGGACCAAACCTC	133 bp	Covers one half of the core EPD promoter sequence for BAX and has shown H3K4me3 enrichment in other cell lines
BAX_CHIP_C__RP	CCGGGTCACGTGAGAGC		
BAX_CHIP_D__FP	GCTCTCACGTGACCCGG	210 bp	Covers the remaining portion of the core EPD promoter, Exon 1 (both with H3K4me3 marks in other cell lines)
BAX_CHIP_D__RP	CCTCAGTGCTTGGAGATCG		
BCL2_CHIP_A_FP	GGAGAACTTCGTAGCAGTCATC	154 bp	Covers 5'UTR and EPD Promoter and part of exonic region
BCL2_CHIP_A_RP	ATAAATGAAGGCAGGACGC		
BCL2_CHIP_B_FP	GCCTTCATTTATCCAGCAGC	109 bp	Covers the exonic region
BCL2_CHIP_B_RP	GAGGGGACGATGAAGGAG		
BCL2_CHIP_C_FP	TCCCCTGTCTCTCTCCTG	98 bp	Covers the exonic region
BCL2_CHIP_C_RP	CTTCTCGGCAATTTACACG		
BCL2_CHIP_D_FP	CTTCTGCGAATACCGGACT	193 bp	Covers the exonic region
BCL2_CHIP_D_RP	ACTTCCCAATGAATCAGGAGT		
BCL2_CHIP_E_FP	ATCAGCTATAACTGGAGAGTGCT	140 bp	Spans the distal part of the first exon
BCL2_CHIP_E_RP	ACCAATTTCCTGTGCAGAG		
