# Supplementary material for: IL-1β/IL-6 inflammatory crosstalk drives DNA methylation and histone remodeling associated with cytokine induced senescence in luminal breast cancer
Source: Inflamm Res. 2026 Oct 17;75(1):246. doi: 10.1007/s00011-026-02391-7 (PMC13676678; doi:10.1007/s00011-026-02391-7)
Supplement: Supplementary file 2 [file 11_2026_2391_MOESM2_ESM.pdf]

IL-1β/IL-6 Inflammatory Crosstalk Drives DNA Methylation and Histone Remodeling Associated with Cytokine Induced Senescence in Luminal Breast Cancer

Satyajit Dey Pereira[1*], Kanaka Sai Ram Padam[1,a], Padival Shruptha[2], Aditi Atreya[1], Manjunath B. Joshi[3], Kapaettu Satyamoorthy[2*]

[1]Department of Cellular and Molecular Biology, Manipal School of Life Sciences, Manipal Academy of Higher Education, Manipal-576104, India

[2]SDM Centre for Cellular and Molecular Sciences, SDM College of Medical Sciences and Hospital, Shri Dharmasthala Manjunatheshwara (SDM) University, Dharwad, India

[3]Department of Ageing Research, Manipal School of Life Sciences, Manipal Academy of Higher Education, Manipal-576104, India

[a]Present address: Herman B Wells Center for Pediatric Research, Department of Pediatrics, Indiana University School of Medicine, Indianapolis, IN 46202, USA

* Corresponding authors

Satyajit Dey Pereira
Department of Cell and Molecular Biology
Manipal School of Life Sciences
Manipal Academy of Higher Education
Manipal, India
Email : Satyajit.pereira@learner.manipal.edu
Satyajitdp12@gmail.com

Dr. Kapaettu Satyamoorthy
SDM College of Medical Sciences and Hospital,
Sri Dharmasthala Manjunatheshwara (SDM) University,
Manjushree Nagar, Sattur,
Dharwad, Karnataka, India-580009
Email: ksatyamoorthy@sdmuniversity.edu.in
ksatyamoorthy@yahoo.com

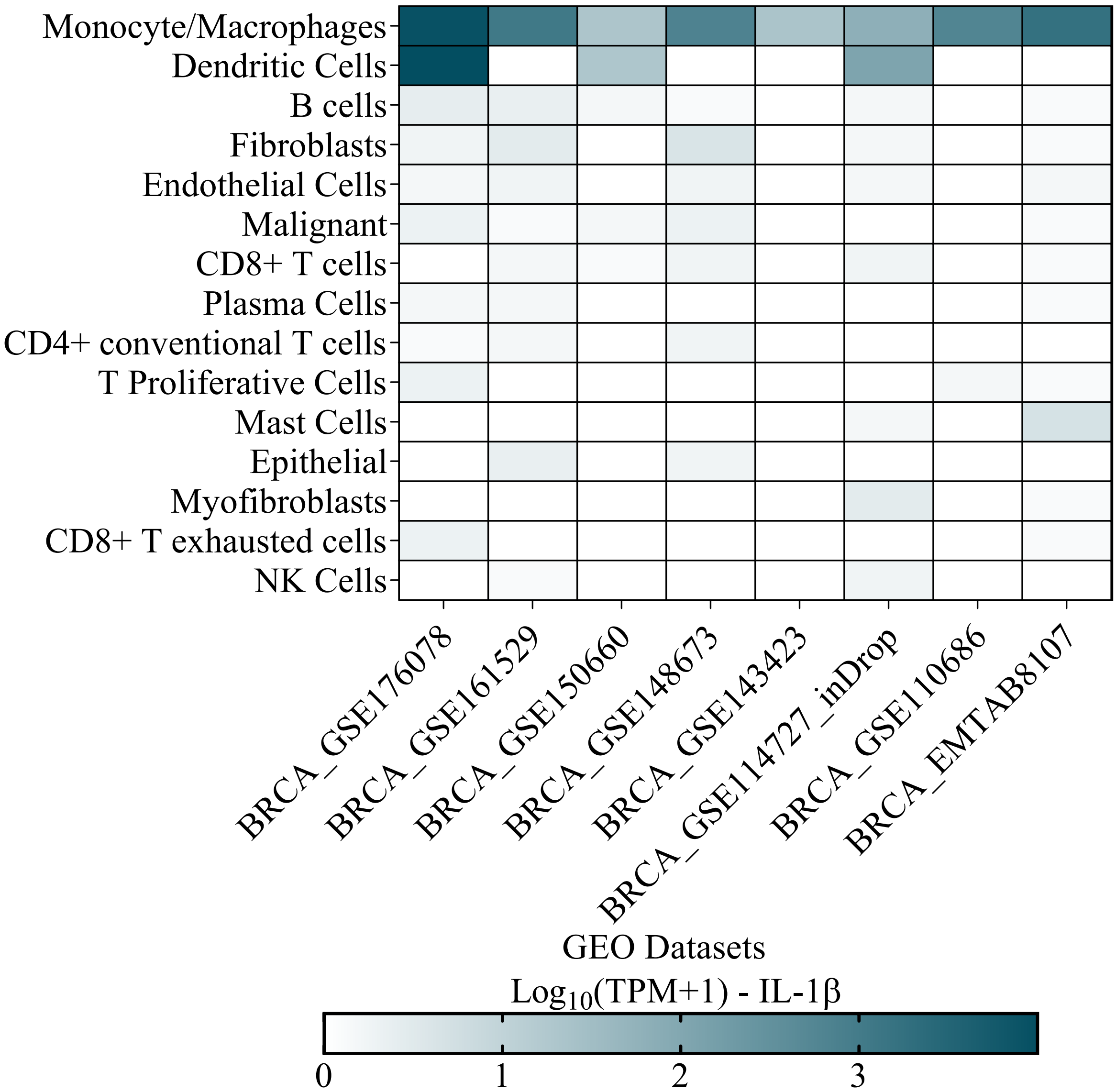

Supplementary Fig. S1 Expression profile of *IL1B* across breast tumour microenvironment constituent cells (stromal, immune and malignant cells) in publicly available breast cancer single-cell RNA sequencing datasets obtained from the TISCH2 database. *IL1B* expression is predominantly enriched within monocyte/macrophage populations across multiple independent breast cancer datasets, supporting myeloid cells as a major source of IL-1β within the breast tumour microenvironment.

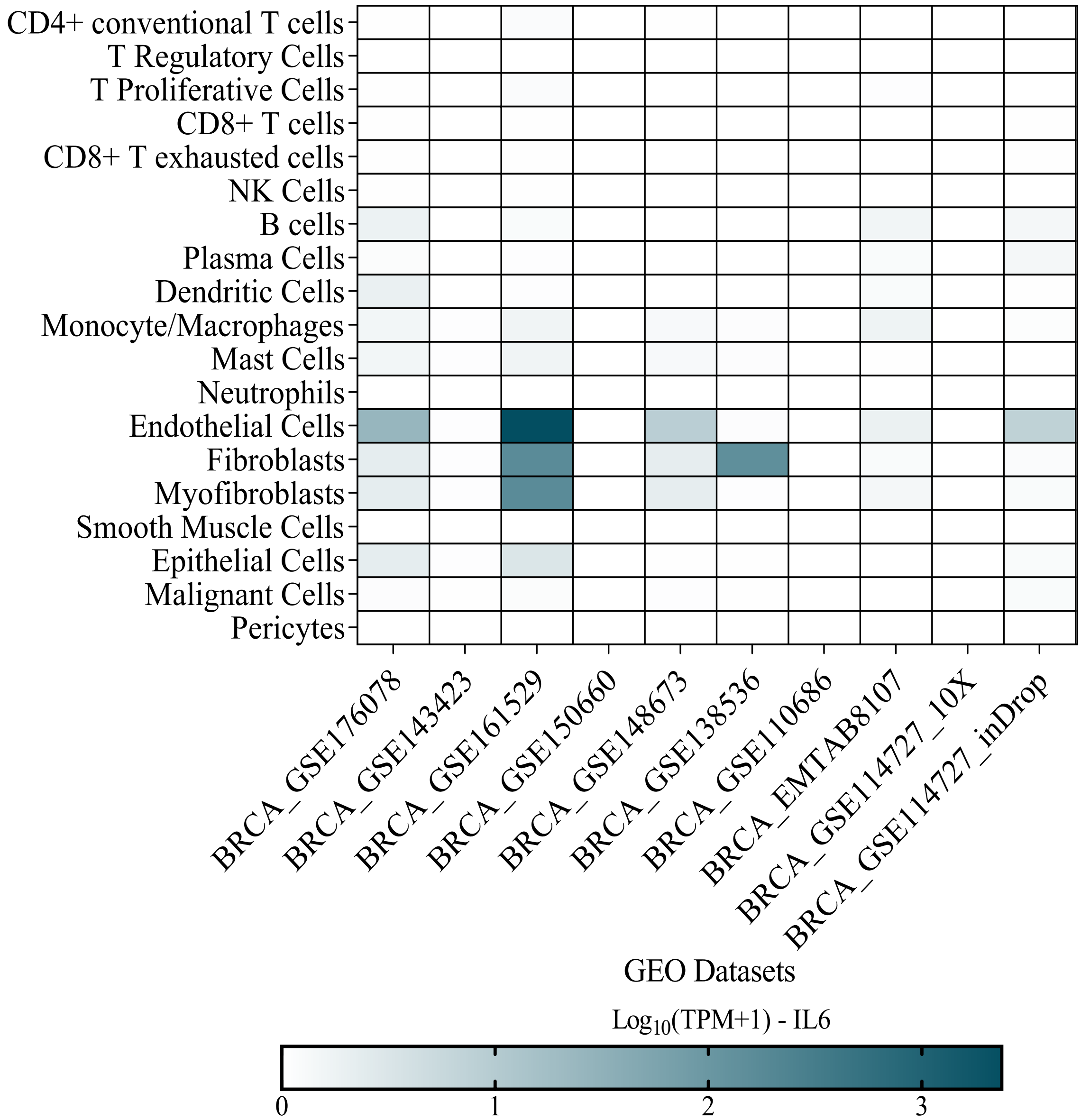

Supplementary Fig. S2 Expression profile of *IL6* across breast tumour microenvironment constituent cells (stromal, immune and malignant cells) in publicly available breast cancer single-cell RNA sequencing datasets obtained from the TISCH2 database. *IL6* expression is predominantly detected within endothelial cells and cancer associated fibroblasts populations across multiple independent breast cancer datasets within the breast tumour microenvironment.

(A)

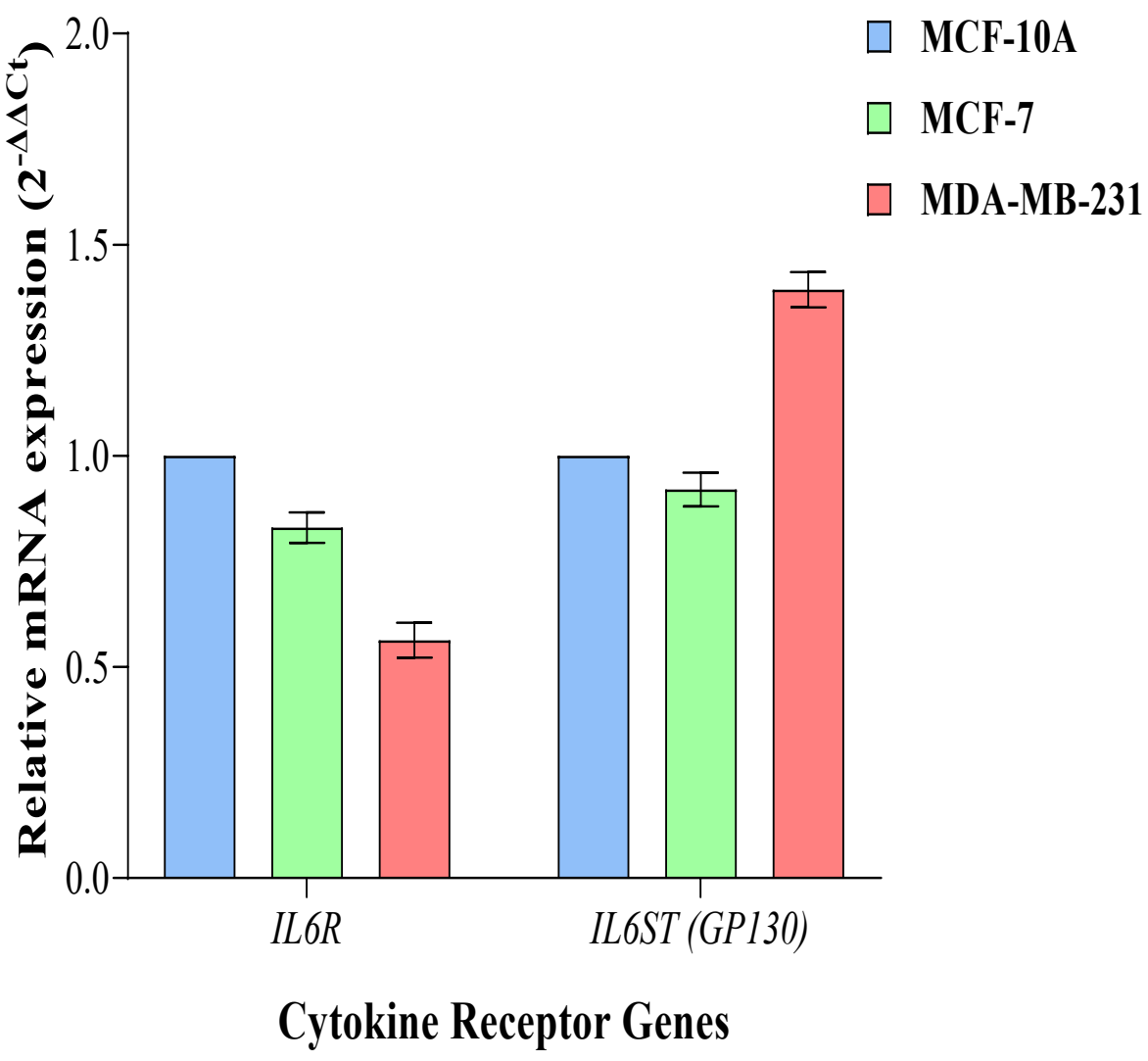

(B)

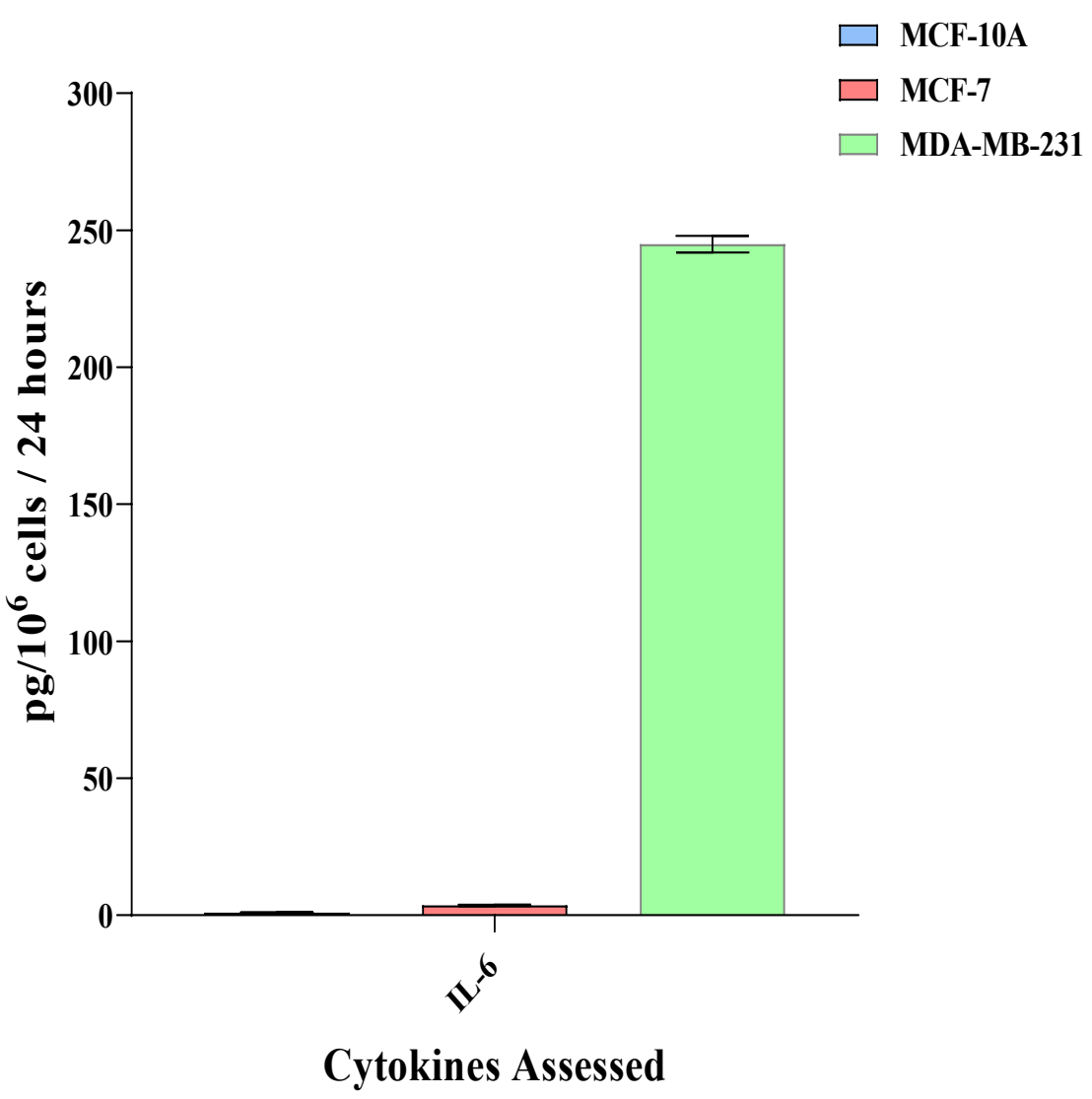

Supplementary Fig. S3 (A) qPCR validation IL-*6R* and *IL6ST (GP130)* receptor expression in breast epithelial and breast cancer cell lines. Relative mRNA expression ($2^{-\Delta\Delta Ct}$) of *IL6R* and *IL6ST* (GP130) was assessed by qPCR in MCF-10A, MCF-7 and MDA-MB-231 cell lines. Expression values were normalized to MCF-10A cells. (B) Quantification of IL-6 levels (pg/10^6 cells/ 24 hours) in MCF-10A, MCF-7 and MDA-MB-231 non-tumorigenic breast epithelial and cancer cell lines by ELISA.

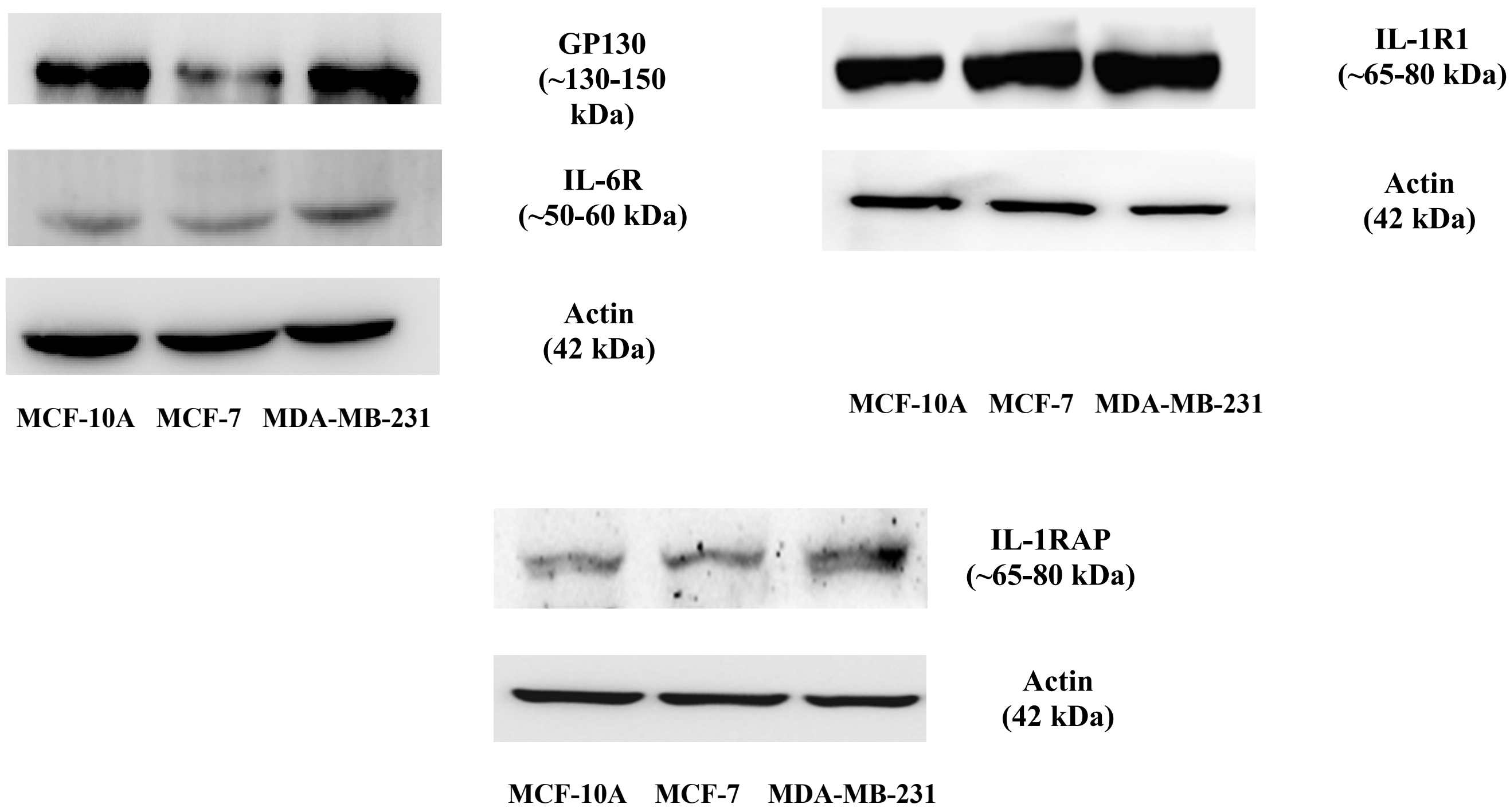

Supplementary Fig. S4 Representative western blot analysis confirming protein expression of GP130 (IL6ST), IL-6R IL1R1, IL1RAP and IL6R in the non-tumorigenic breast epithelial cell line MCF-10A, luminal breast cancer cell line MCF-7, and triple-negative breast cancer cell line MDA-MB-231. The corresponding β-actin loading control for each immunoblot is shown.

a. 24 Hours

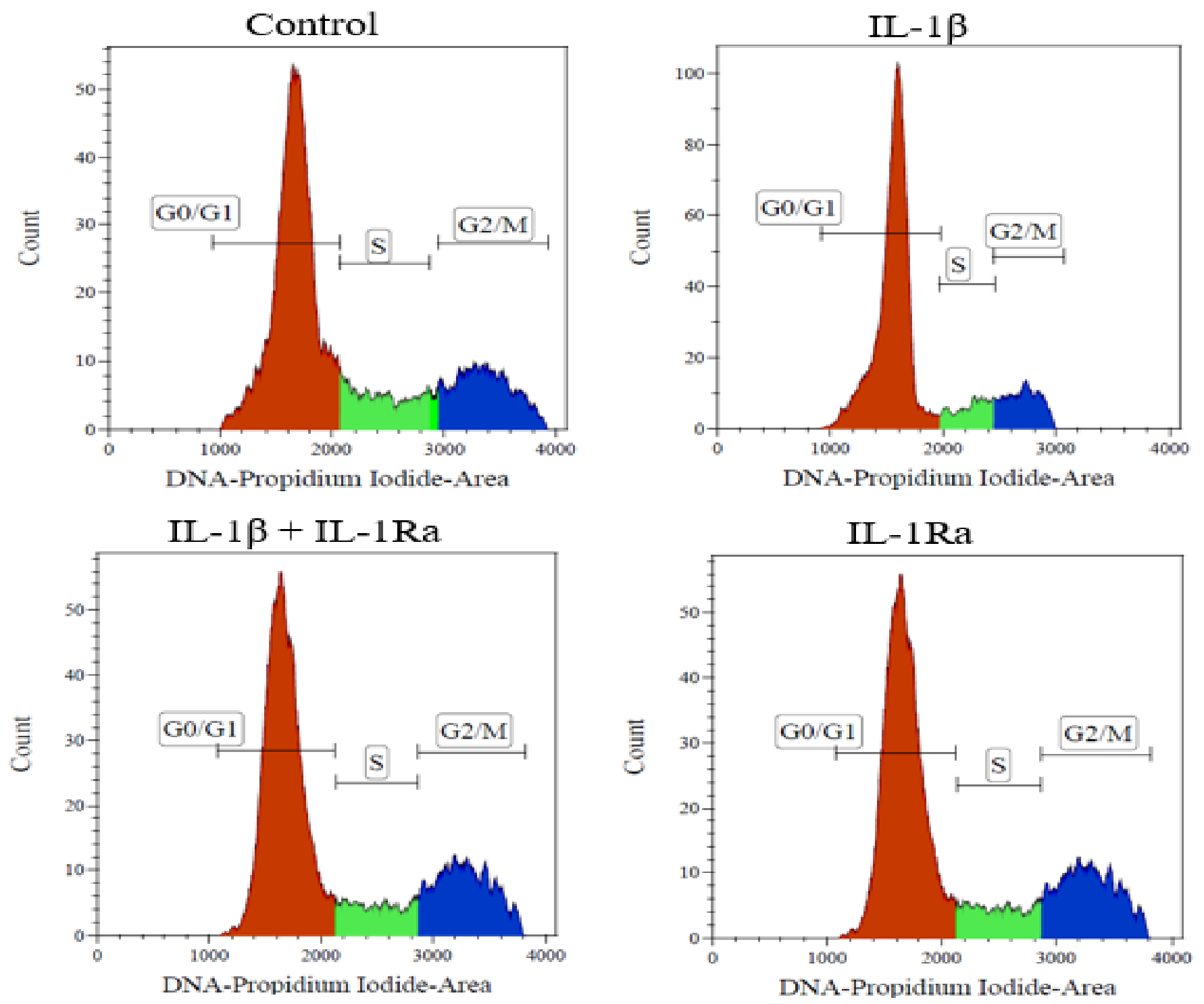

b. 72 hours

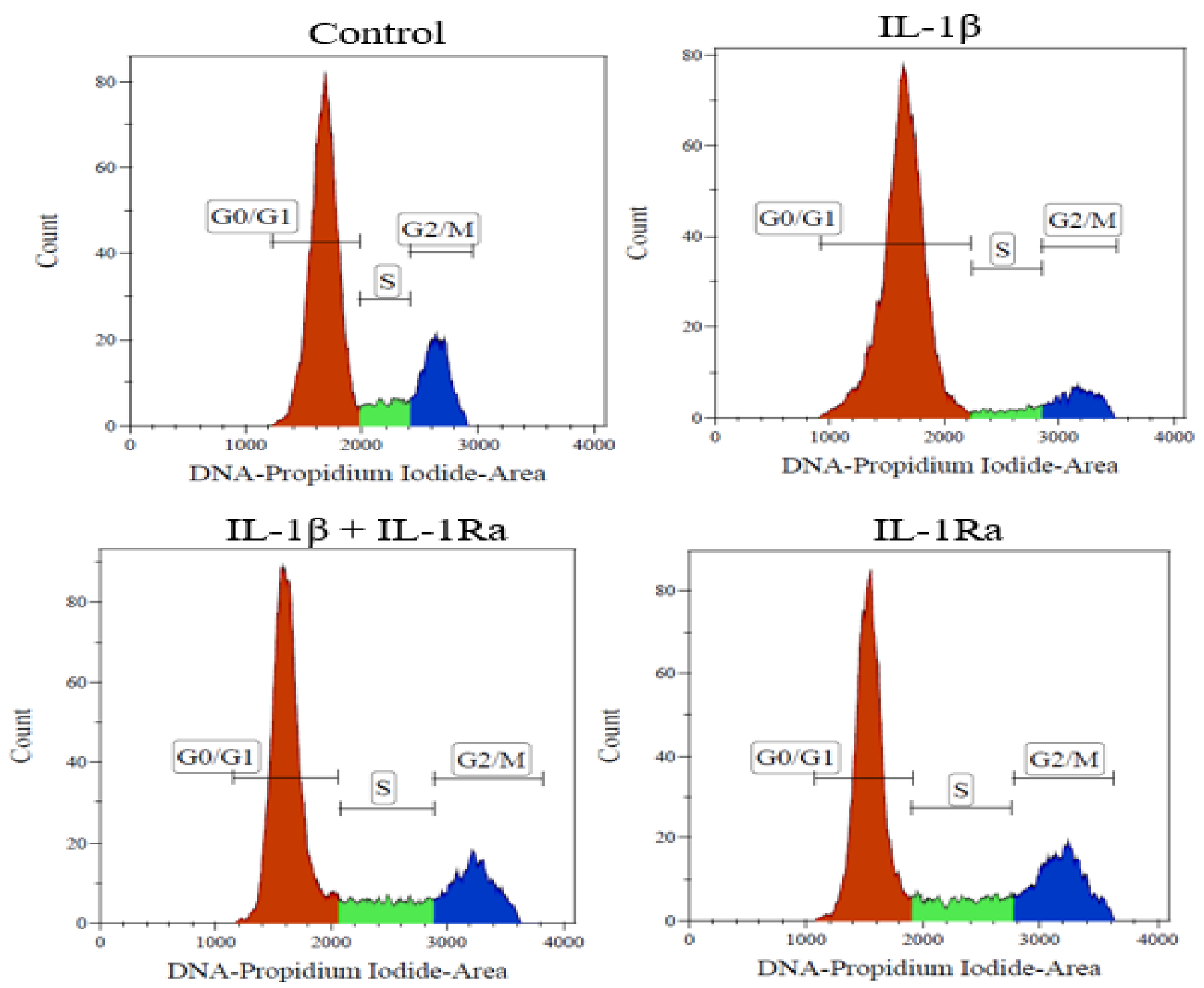

Supplementary Fig. S5 Representative histograms showing DNA content distribution across different phases of the cell cycle under control, IL-1β , IL-1β + IL-1Ra, and IL-1Ra treatments for **a** 24 hours and **b** 72 hours treatment periods in MCF-7 cells.

a. 24 Hours

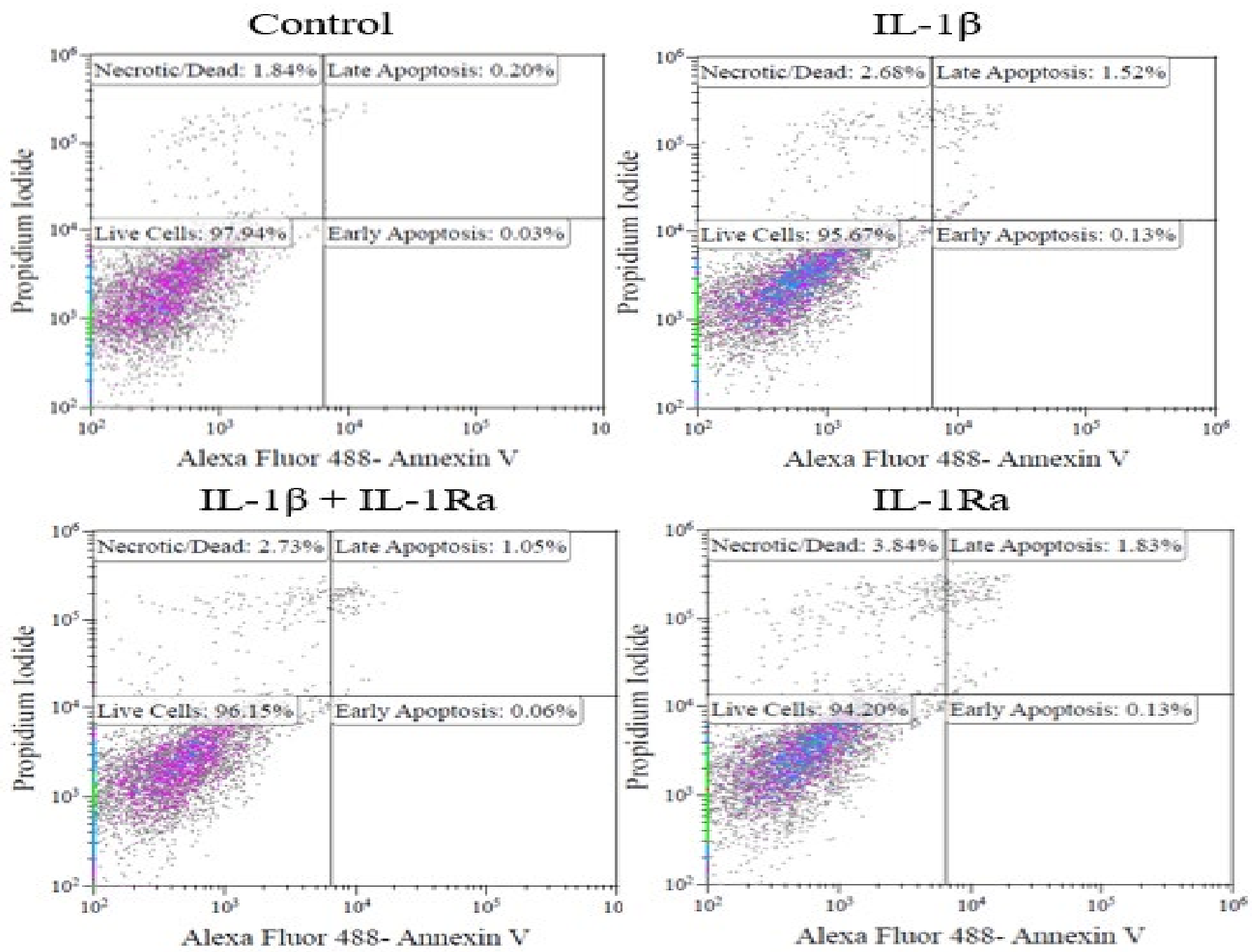

b. 72 hours

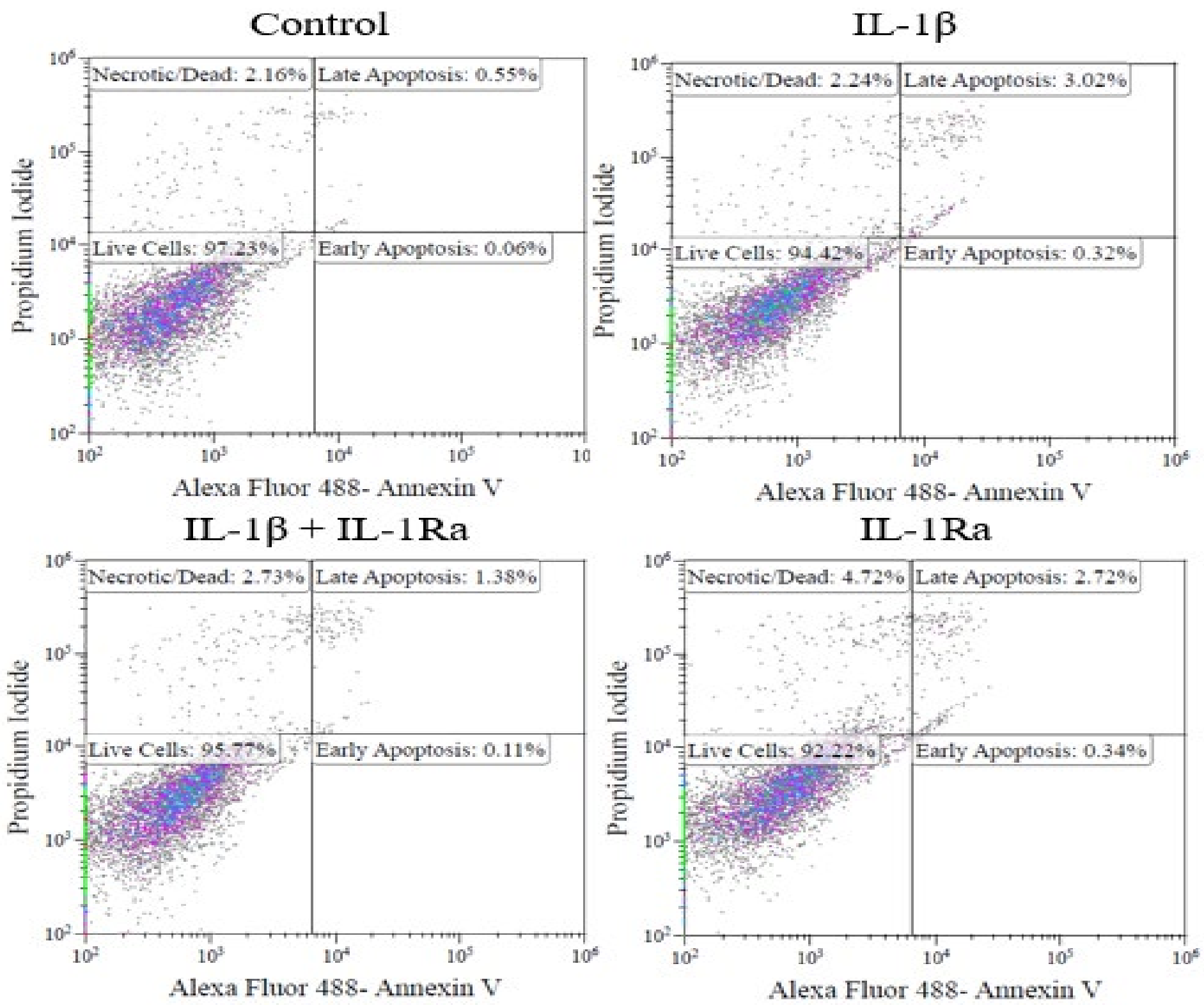

Supplementary Fig. S6 Representative Alexa Fluor 488-Annexin V/PI quadrant density plots showing apoptotic profiles of control, IL-1β treated, IL-1β +IL-1Ra co-treated, and IL-1Ra treated MCF-7 cells post **a** 24-hour and **b** 72-hour treatment periods.

a. 24 Hours

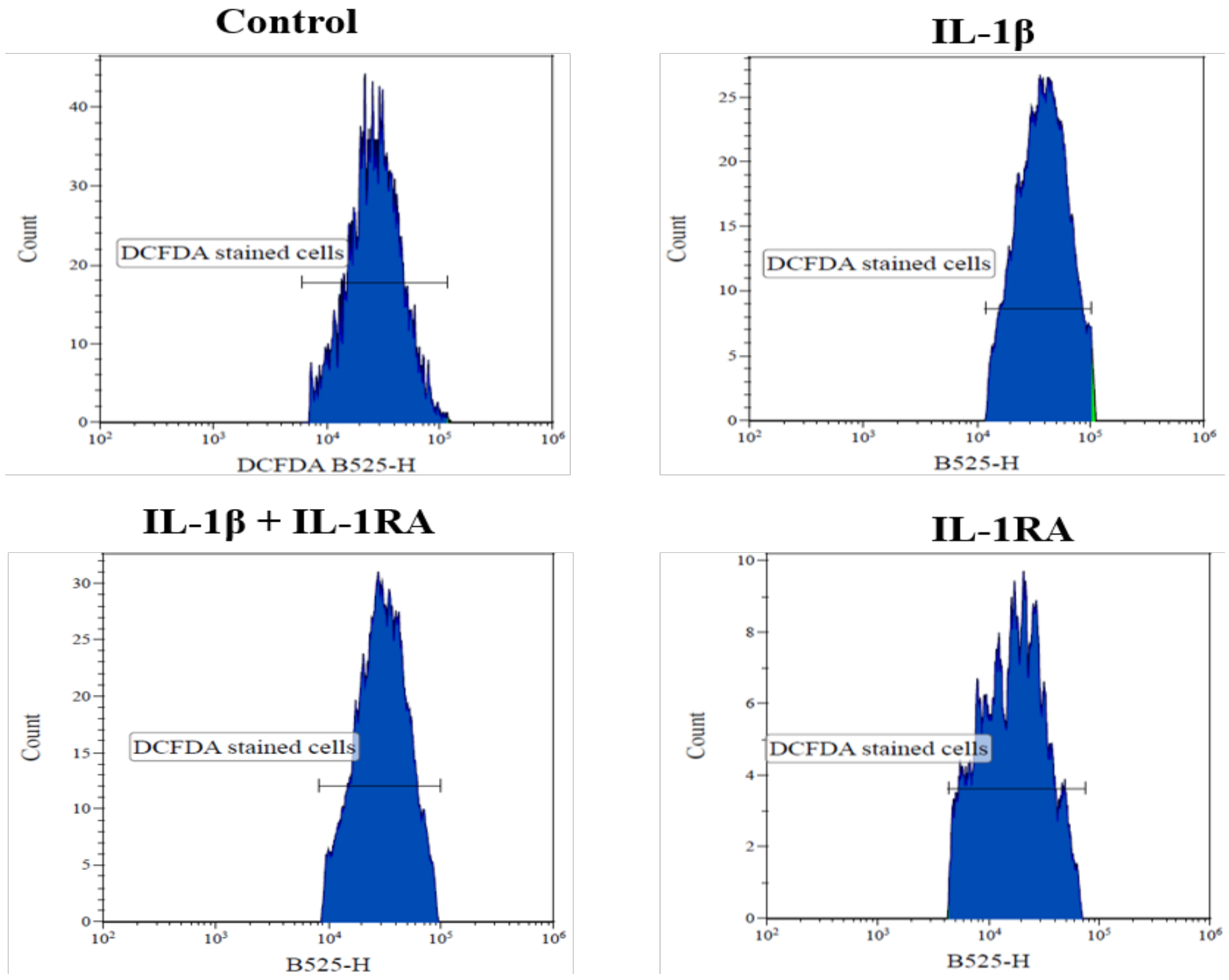

b. 72 Hours

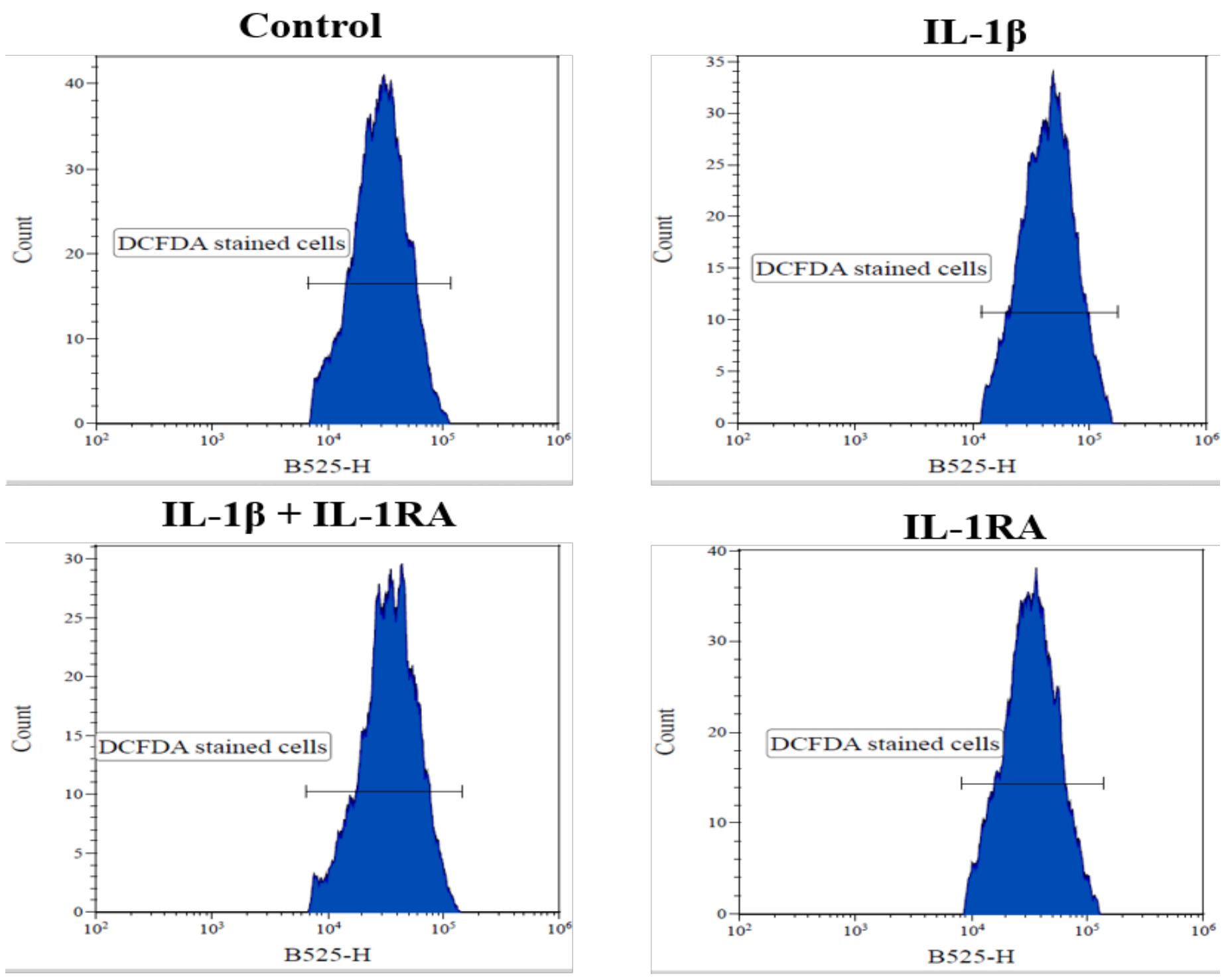

Supplementary Fig. S7 Representative histograms of DCFDA fluorescence to measure Reactive Oxygen Species for control, IL-1β, IL-1β + IL-1RA, and IL-1RA only treatment groups at **a** 24 hours and **b** 72 hours timepoints.

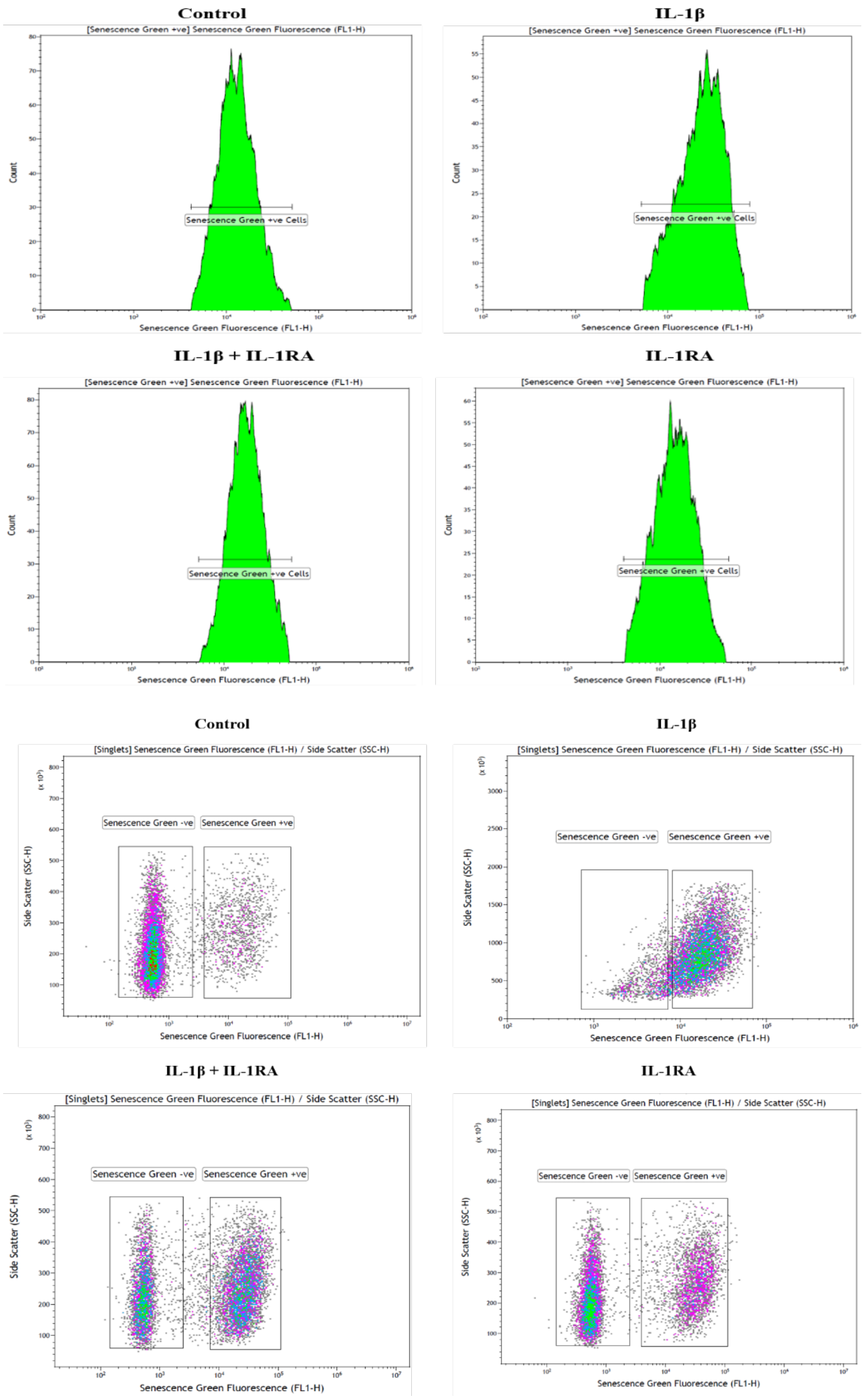

Supplementary Fig. S8 Representative histograms and scatter plots showing Senescence Green fluorescence distribution and Median Fluorescence Intensity (X-Med) following flow cytometric detection of cellular senescence via β-galactosidase hydrolysis activity in Control, IL-1β, IL-1β + IL-1RA, and IL-1RA treated MCF-7 cells after 72 hours of treatment.

a.

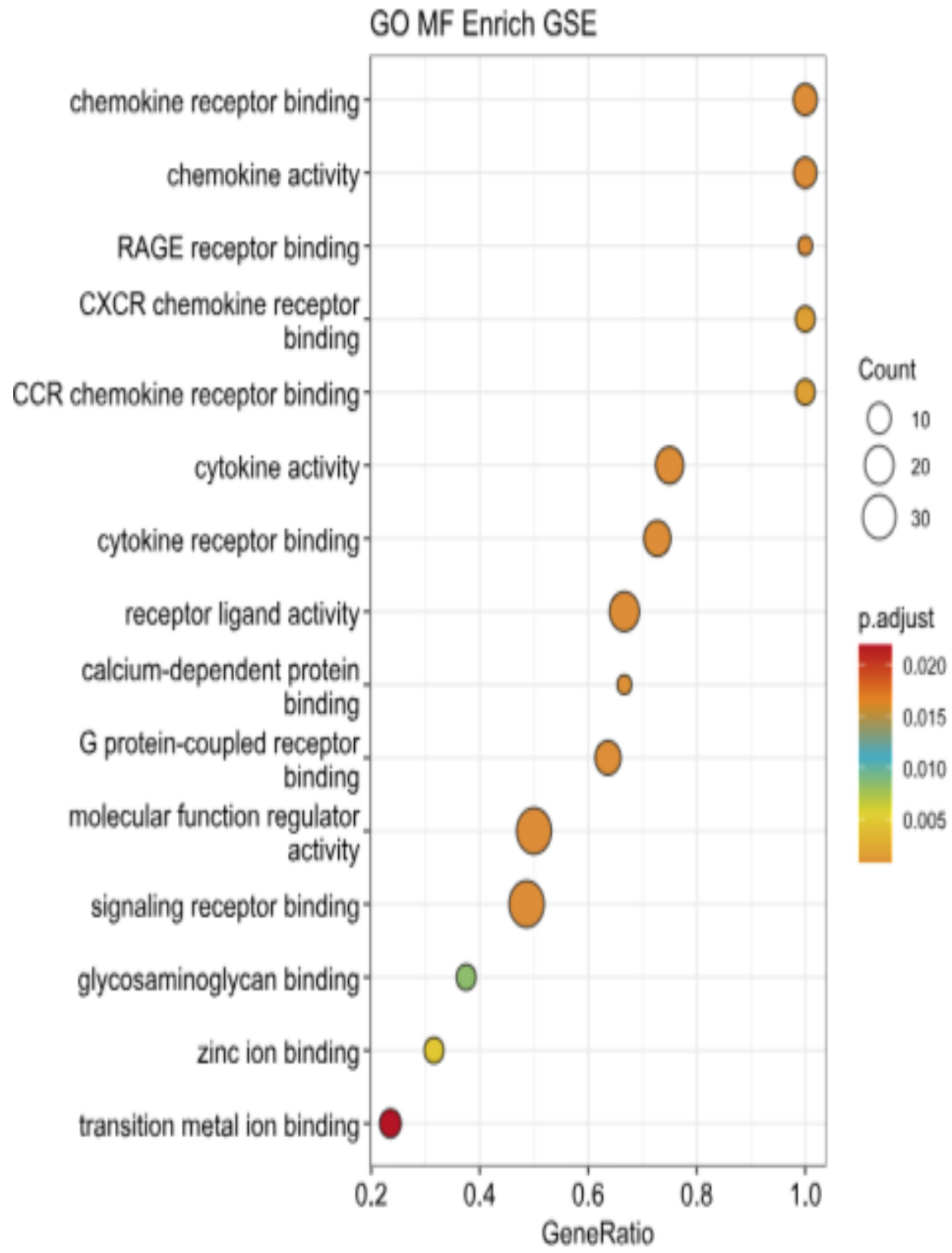

b.

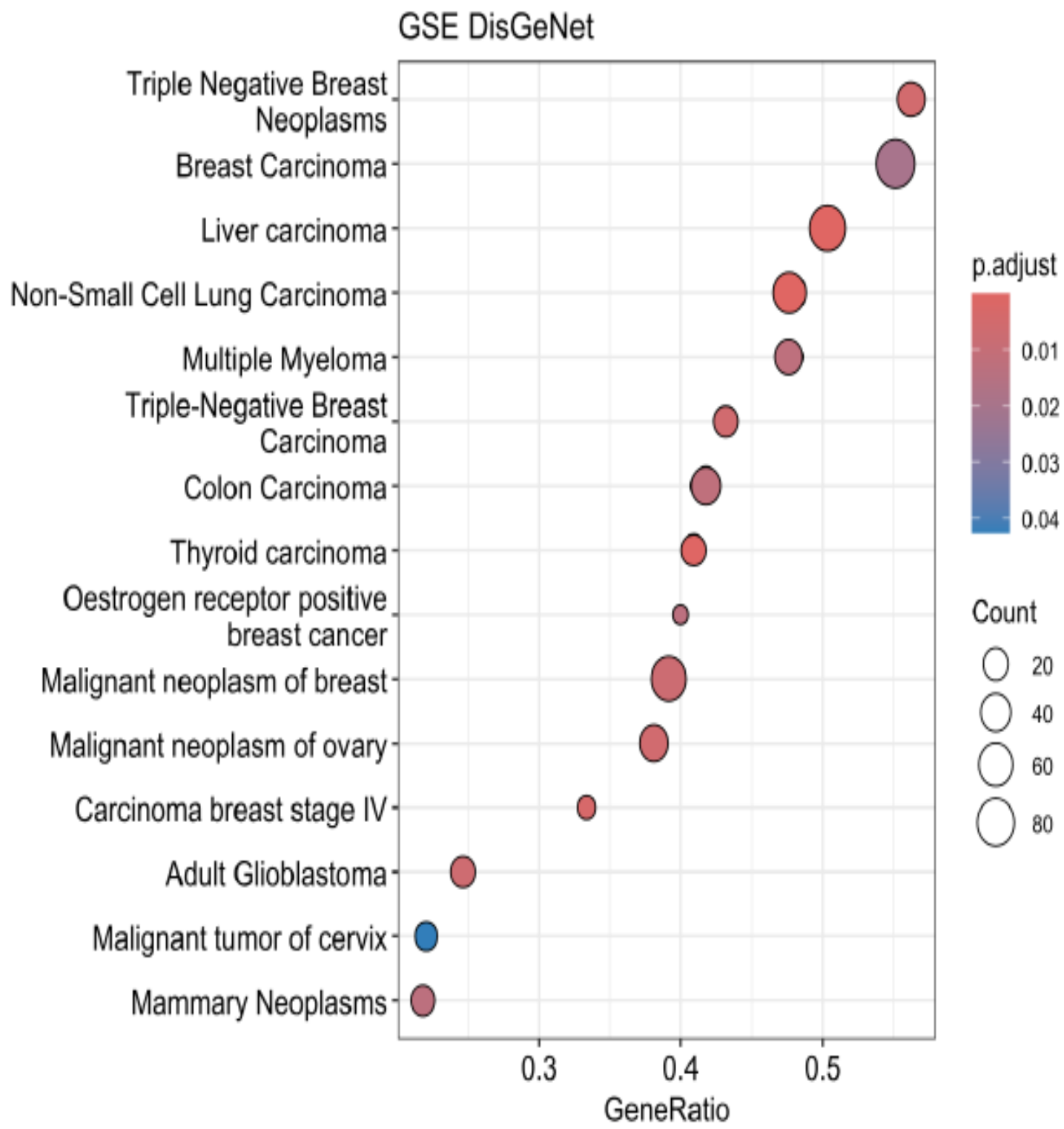

Supplementary Fig. S9 Functional enrichment analysis of IL-1β induced transcriptional alterations in MCF-7 post 24-hour treatment using gene ontology and pathway prioritization tools: **a** molecular function and **b** DisGeNET-based identification of cancer-associated genes. Adjusted p-value of < 0.05 (Benjamini-Hochberg correction) was considered statistically significant.

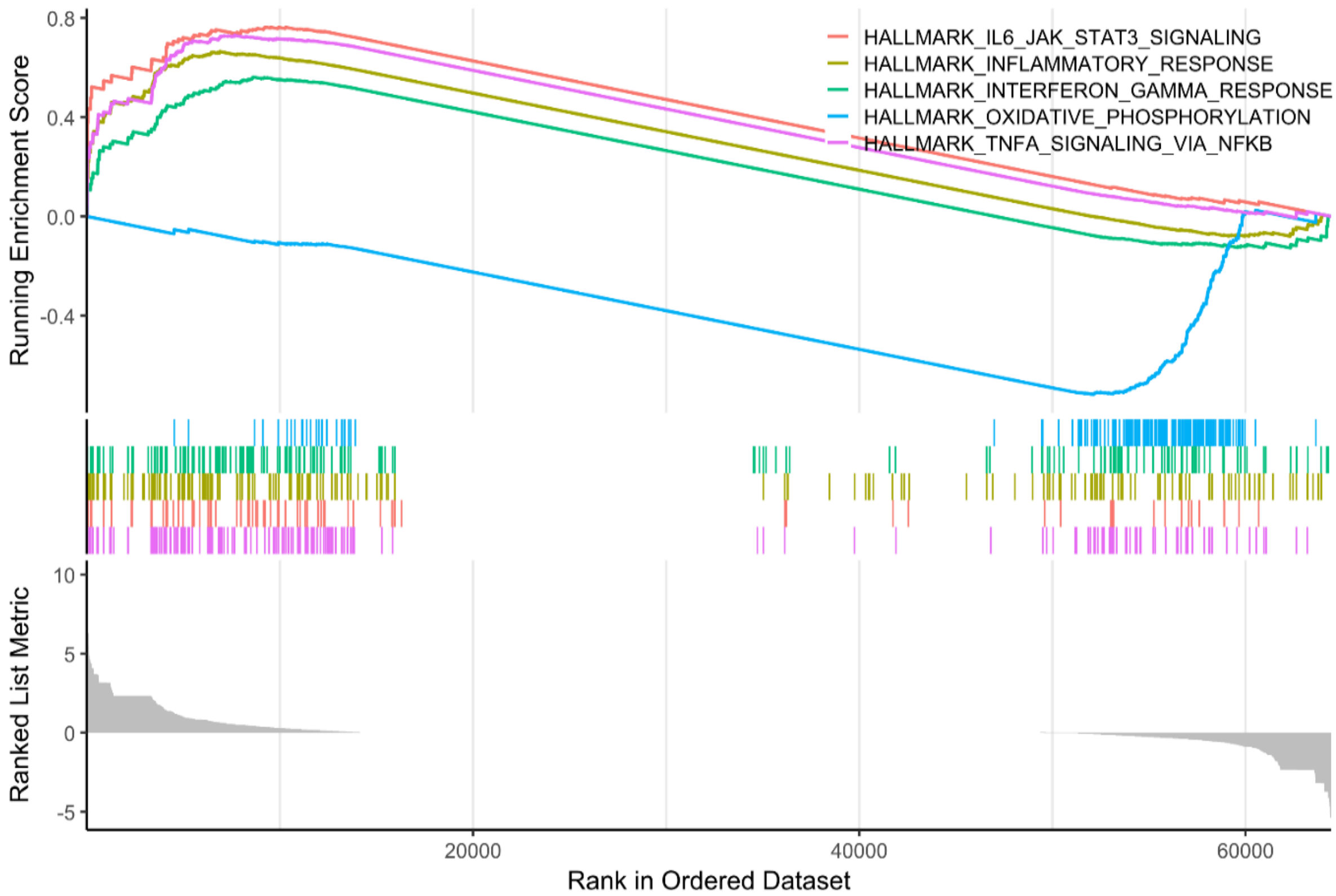

Supplementary Fig. S10 GSEA of ranked gene expression changes in IL1β -treated versus control MCF7 cells using the MSigDB Hallmark collection. Key upregulated hallmarks included Inflammatory Response, IL6/STAT3 Signaling, TNF Signaling via NF-κB, and Interferon-γ Response, this suggests IL1β driven activation of pro-inflammatory and pro-survival pathways while Conversely, Oxidative Phosphorylation was significantly downregulated, indicating a potential metabolic shift towards glycolysis. Significance was determined by normalized enrichment score (NES) and FDR q-value (threshold: FDR ≤ 0.25).

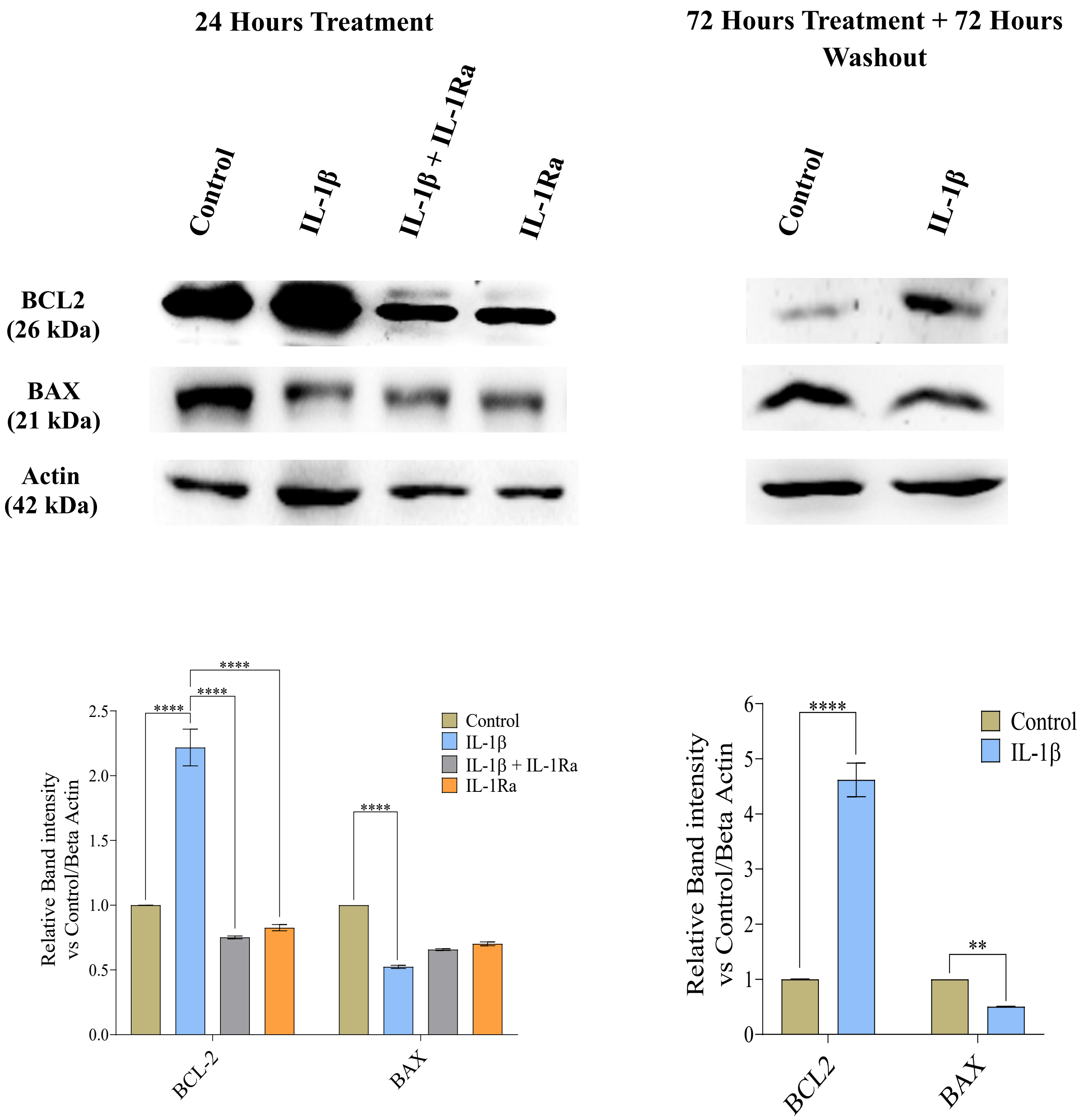

Supplementary Fig. S11 Western blotting and densitometric analysis for BCL2 and BAX in MCF-7 cells treated with IL-1β (10ng/ml) for 24 hours and 72 hours followed by a 72-hour withdrawal period in presence or absence of IL-1Ra, the receptor antagonist of IL-1β (20ng/ml). Two-tailed unpaired t Test with Welch's correction was applied to determine statistical significance (****$p \leq 0.0001$' ***$p \leq 0.001$, **$p \leq 0.01$, p *≤ 0.05).

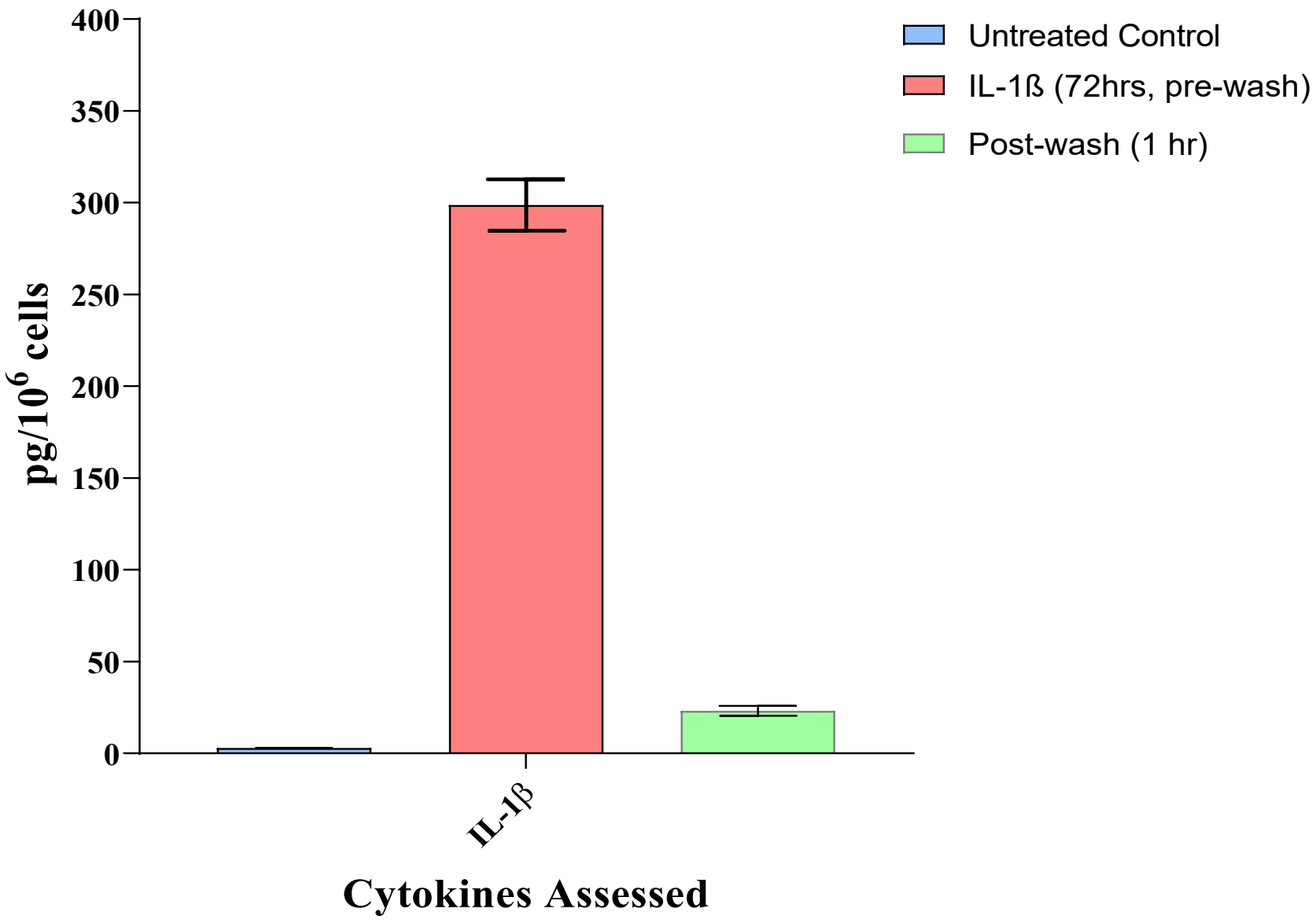

Supplementary Fig. S12 ELISA verification of IL-1β removal following medium replacement for 72 hours treatment followed by 72 hours cytokine washout experiments. Culture supernatants were collected from untreated control cells and cells treated with IL-1β (10 ng/ml) every 24 hours for a 72 hour period immediately after completion of the 72 hour period - IL-1β (72 hours, pre-wash), and 1 h after replacement of the IL-1β-containing medium with fresh cytokine-free medium (post-wash). ELISA analysis confirmed efficient removal of exogenous IL-1β following the washout procedure.

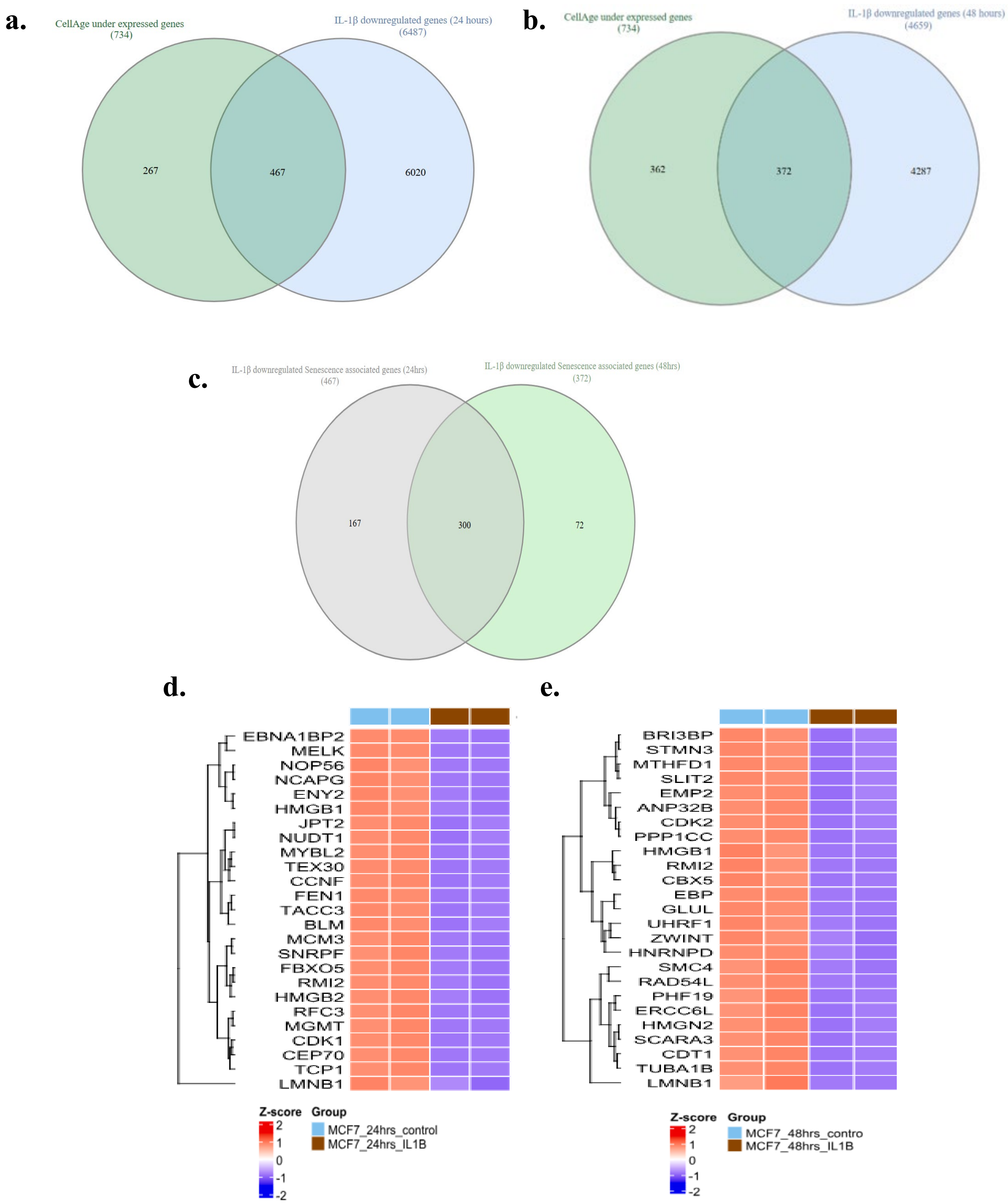

Supplementary Fig. S13 Venn diagrams showing overlap between CellAge under expressed genes and IL-1β induced downregulated genes at **a** 24 hours and **b** 48 hours. **c** Venn diagram highlighting the common subset of downregulated senescence-associated genes shared between 24- and 48-hour treatments. Heatmaps of the top 25 downregulated senescence-associated genes at **d** 24 hours and **e** 48 hours ranked by ΔZ-score (IL-1β – control). 467 and 372 out of 734 reported under expressed genes were downregulated at 24- and 48- hours respectively. 300 of these genes were in common between both treatment timepoints.

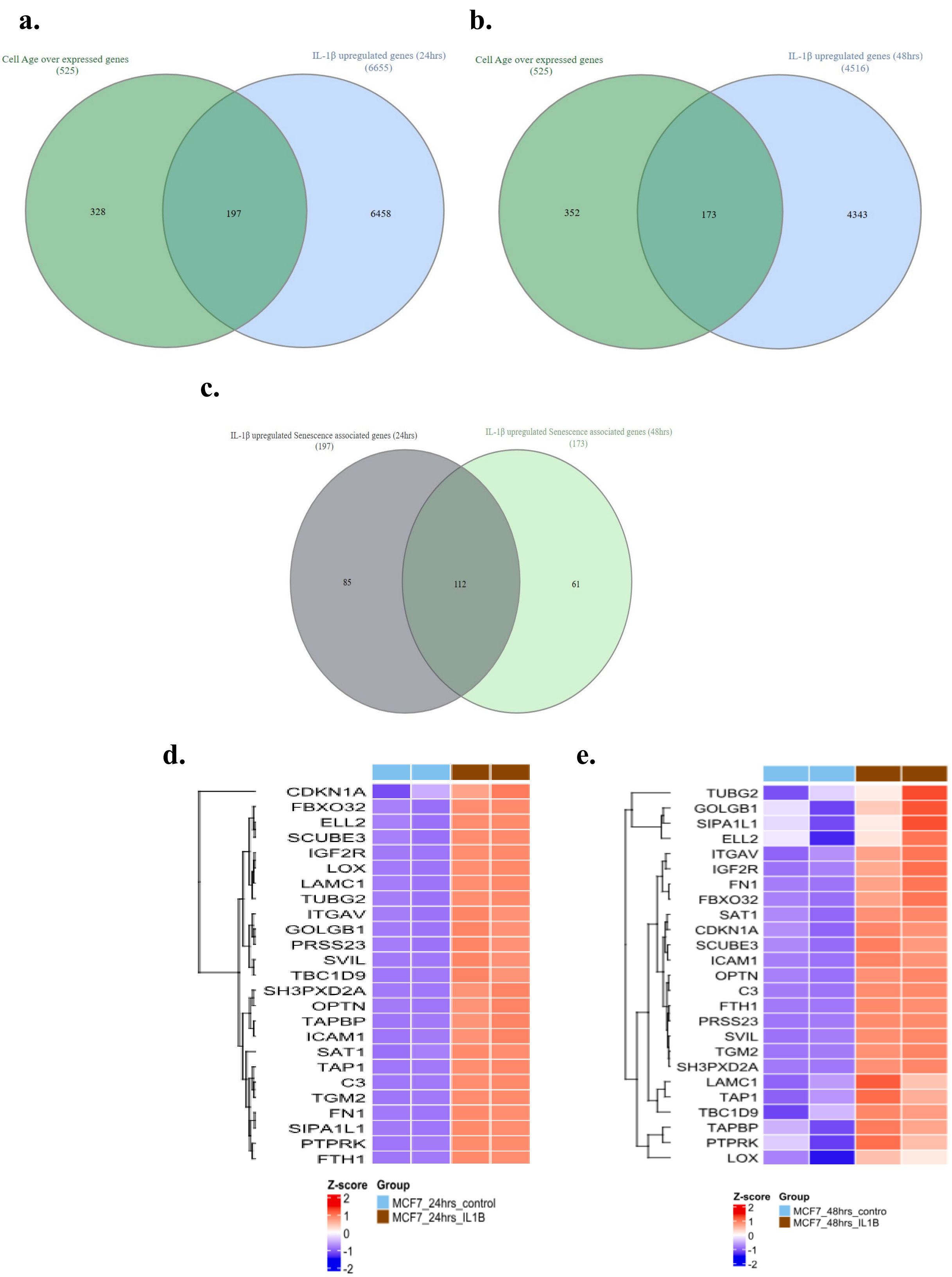

Supplementary Fig. S14 : Venn diagrams showing overlap between CellAge over expressed genes and IL-1β induced upregulated genes at **a** 24 and **b** 48 hours. **c** Venn diagram highlighting the common subset of upregulated senescence-associated genes shared between 24- and 48-hour treatments. Heatmaps of the top 25 upregulated senescence-associated genes at **d** 24 and **e** 48 hours ranked by ΔZ-score (IL-1β – control). 197 and 173 out of 525 over expressed genes were upregulated at 24- and 48- hours respectively.

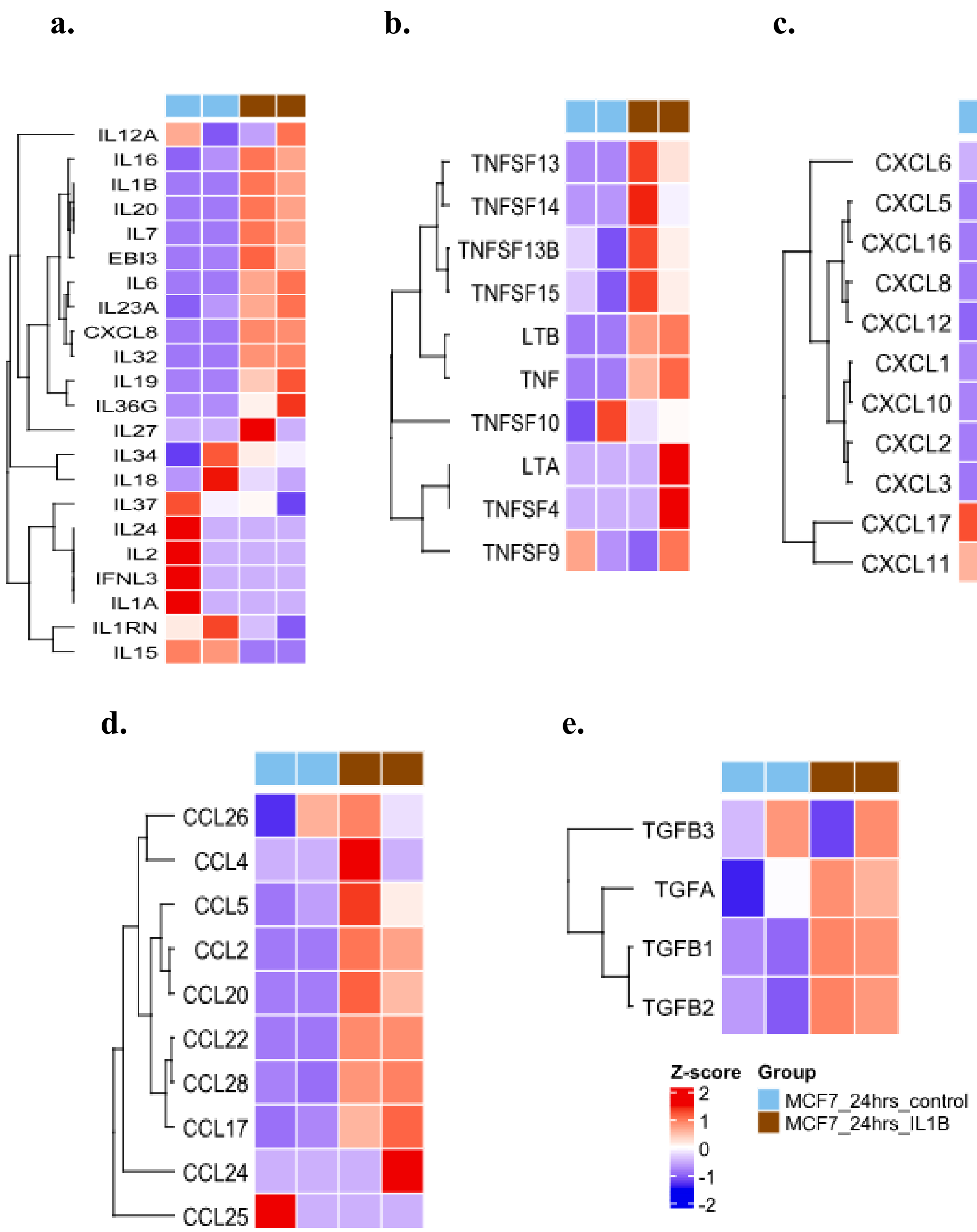

Supplementary Fig. S15 Profiling of differential expression patterns of cytokine and chemokine family/superfamily members in MCF7 cells post 24 hours IL1β treatment. **a** Interleukin family, **b** Tumor Necrosis Factor (TNF) superfamily, **c** CXC chemokines, **d** CC chemokines, **e** Transforming Growth Factor (TGF) family. IL-1β treatment drives an elevation of predominantly pro-inflammatory and pro-tumorigenic cytokines and chemokines with increased expression of several ILs, TNFs, and CXCL/CCL chemokines, alongside TGF family members that support EMT, immune evasion, immune suppression, and metastatic progression. Several of the upregulated cytokines including *IL-6, CXCL8 (IL-8)*, *TNF-α, CXCL1 (GRO- α), CXCL2 (MIP-2α), CCL2* and *CCL5* are upregulated. While *IL-15* is downregulated as often reported in case of senescence. Additionally, *IL1RN* which encodes IL-1Ra the receptor antagonist cytokine to IL-1β that plays a key role in maintaining a regulated and balanced inflammatory response is downregulated.

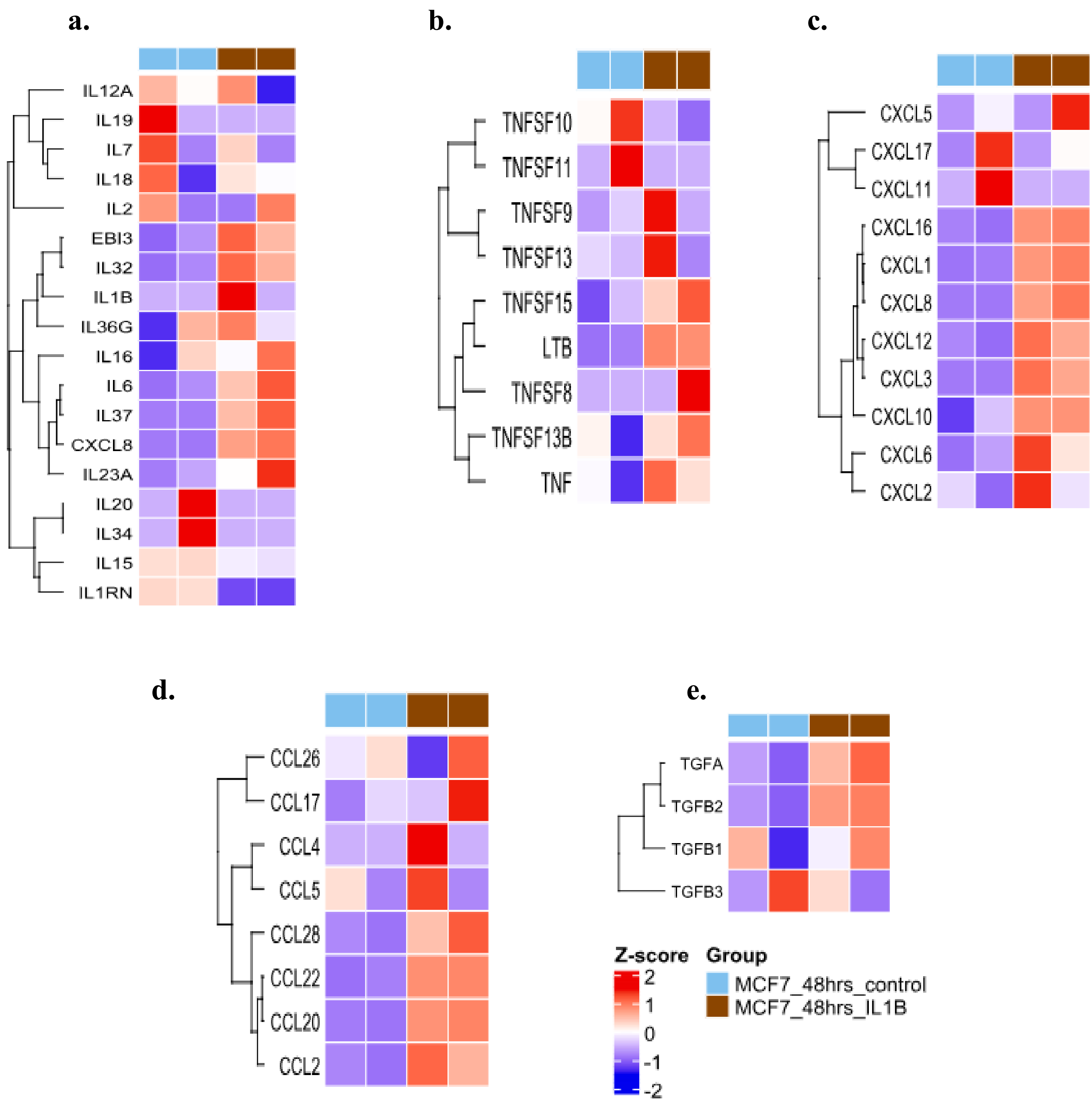

Supplementary Fig. S16 Profiling of differential expression patterns of cytokine and chemokine family/superfamily members in MCF7 cells post 48 hours IL-1β treatment. **a** Interleukin family, **b** Tumor Necrosis Factor (TNF) superfamily, **c** CXC chemokines, **d** CC chemokines, **e** Transforming Growth Factor (TGF) family. IL-1β treatment drives an elevation of predominantly pro-inflammatory and pro-tumorigenic cytokines and chemokines with increased expression of ILs, TNFs, and CXCL/CCL chemokines, alongside TGF family members that support EMT, immune evasion, immune suppression, and metastatic progression. Several of the senescence associated secretory profile cytokines upregulated at 24 hours, show continued over expression at 48-hours including *IL-6, CXCL8 (IL-8) , TNF-α, CXCL1 (GRO-α) and CCL2.* While *IL-15* is downregulated. *IL1RN* downregulation observed at 24 hours persists at the 48-hour timepoint with reduced IL1RN expression suggesting a highly pro-inflammatory state .

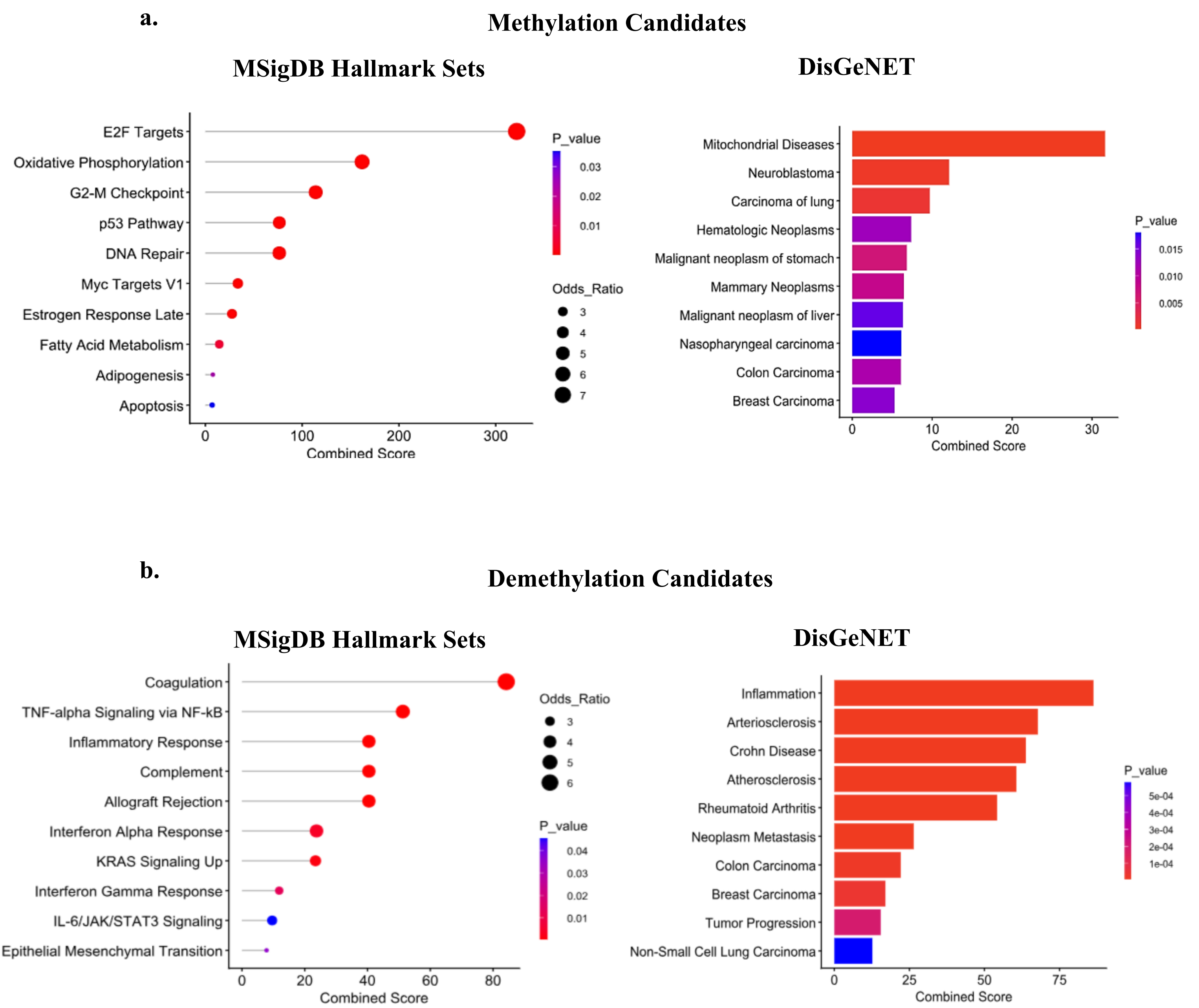

Supplementary Fig. S17 Functional enrichment analysis of putative methylation and demethylation candidate genes identified by integrating IL-1β-induced transcriptomic alterations with the NCI-60 DNA methylation dataset in MCF-7 cells**.** Gene sets associated with **a.** methylation candidate genes and **b.** demethylation candidate genes were analysed using MSigDB Hallmark gene sets and DisGeNET disease enrichment. These analyses complement the GO Biological Process and KEGG pathway enrichment presented in the main manuscript (Fig. 4d)

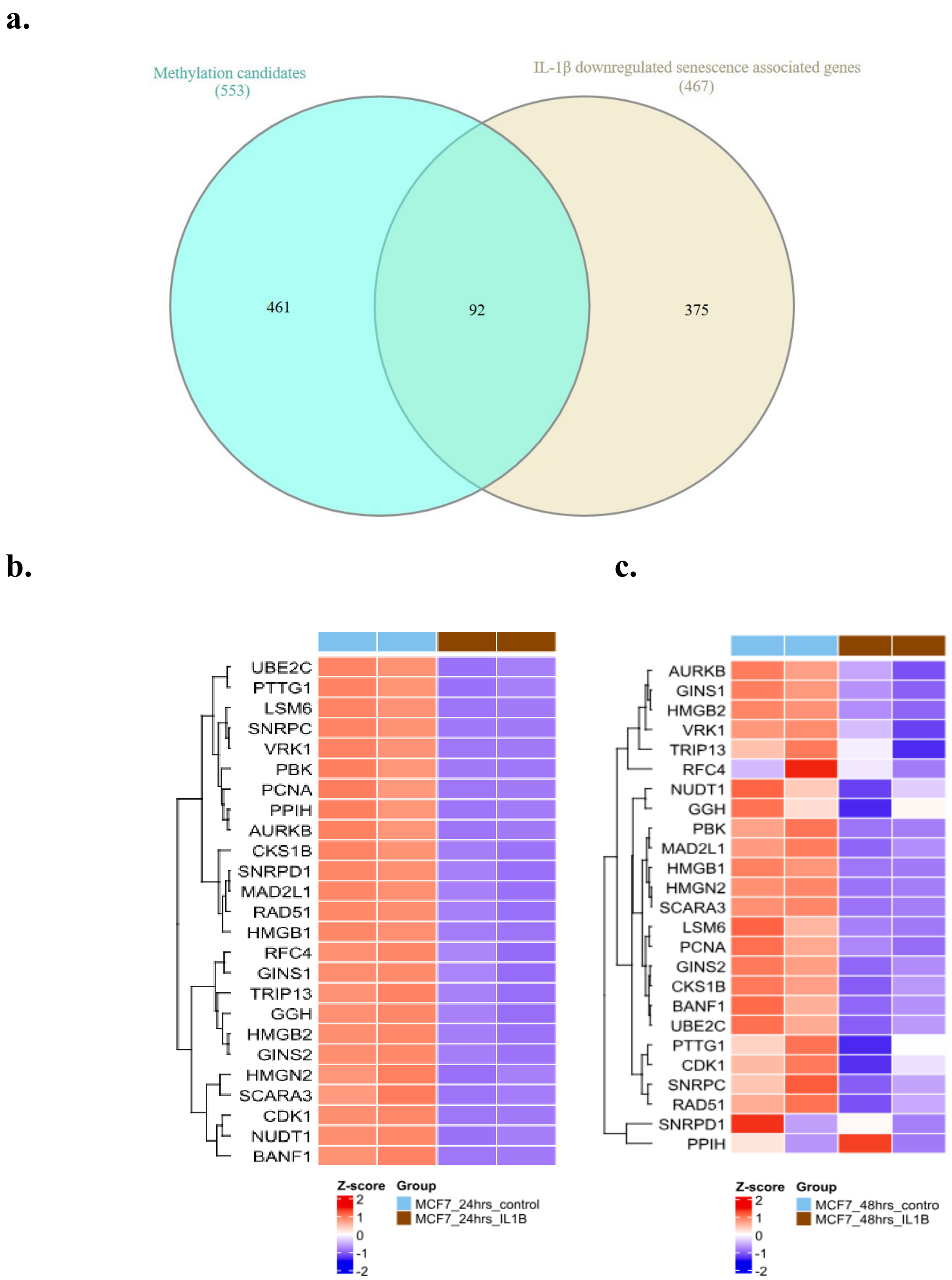

Supplementary Fig. S18 **a** Venn diagram showing the overlap between potential IL-1β induced methylation candidates and genes downregulated post IL-1β induction and associated with senescence as per CellAge database, highlighting a core set of 92 genes. **b** Heatmap of the top 25 most downregulated genes ranked by log_2 fold-change at 24 hours, with z-scores plotted across replicates. These genes show consistent repression following IL-1β treatment and are mainly involved in replication, DNA repair , chromatin architecture regulation and ROS regulation **c** Heatmap of the same top 25 genes at 48 hours induction time-point.

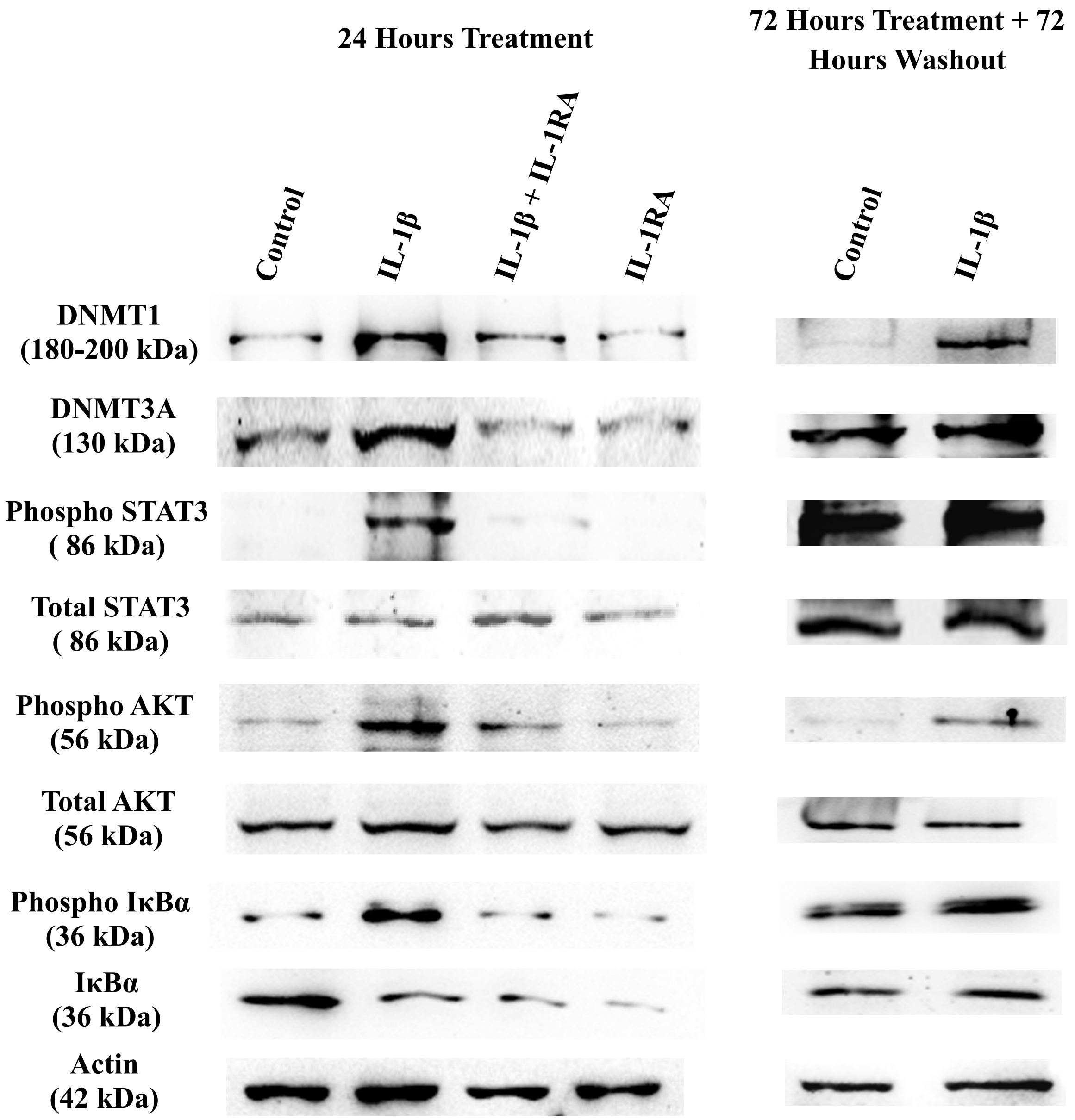

Supplementary Fig. S19 Western blot analysis for DNMT1, DNMT3A, pSTAT3 (Tyr705), total STAT3, pAKT (Ser473), total AKT, pIκBα (Ser36) and total IκBα in MCF-7 cells treated with IL-1β (10ng/ml) for 24 hours and 72 hours treatment followed by 72 hour withdrawal period in presence or absence of IL-1Ra, the receptor antagonist of IL-1β (20ng/ml).

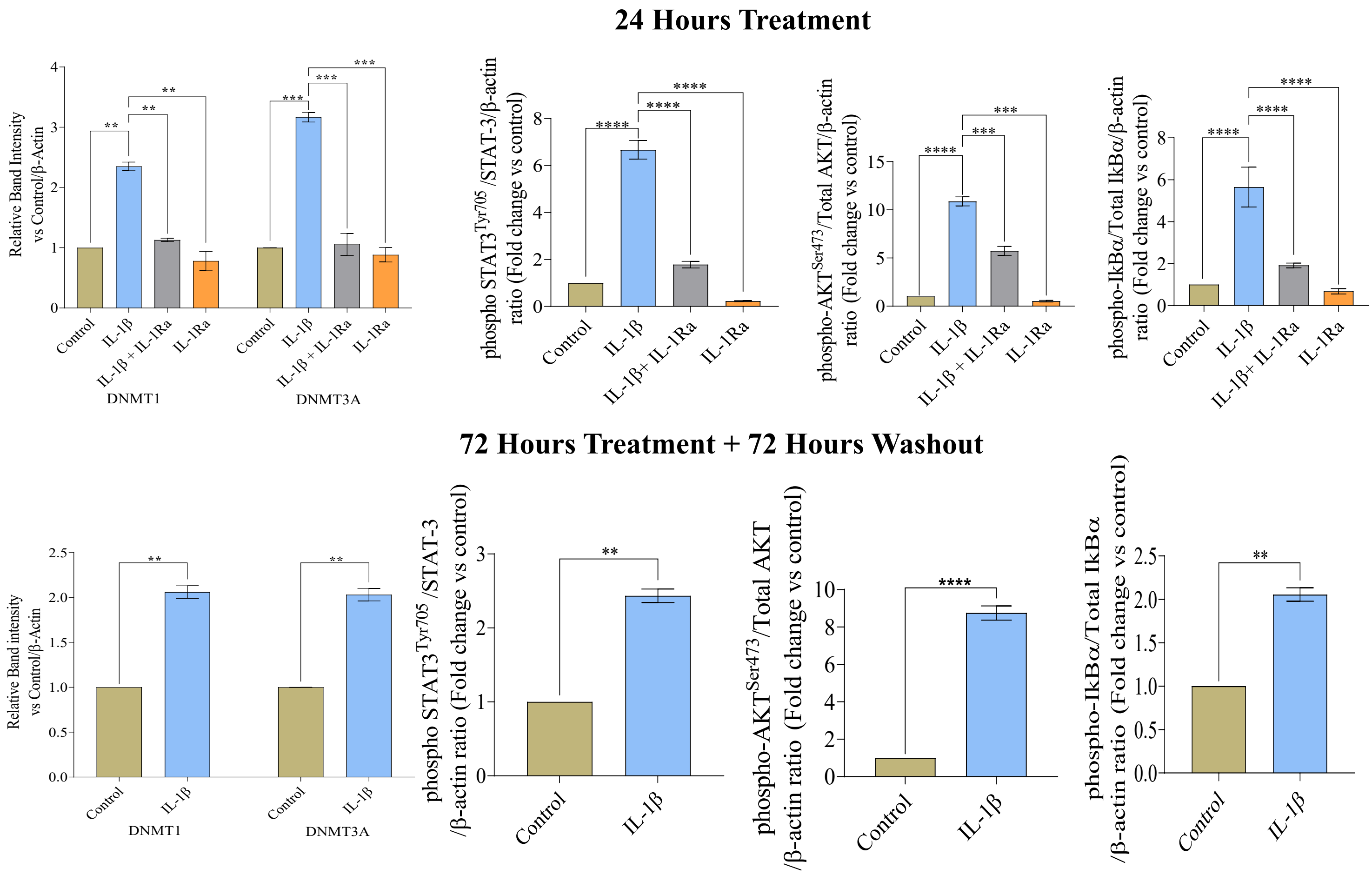

Supplementary Fig. S20 Densitometric analysis for 24-hour treatment and 72-hour treatment followed by 72-hour withdrawal. Two-tailed unpaired t Test with Welch's correction was applied to determine statistical significance (****p ≤ 0.0001' ***p ≤ 0.001, **p ≤ 0.01, p *≤ 0.05).

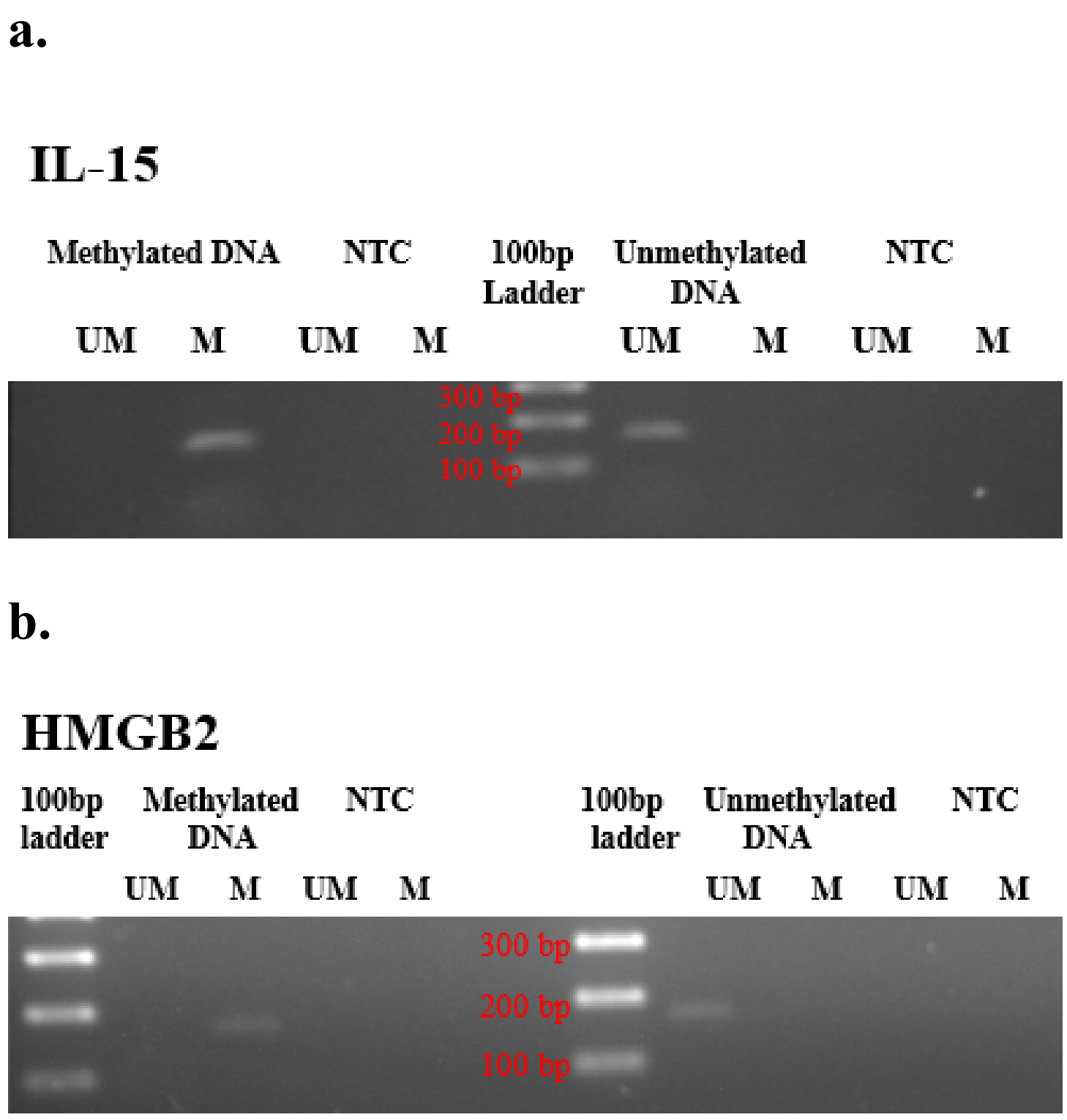

Supplementary Fig. S21 Control amplification using **a** IL-15 and **b** HMGB2 MSP primers on fully methylated and unmethylated human genomic DNA standards to validate primer specificity.

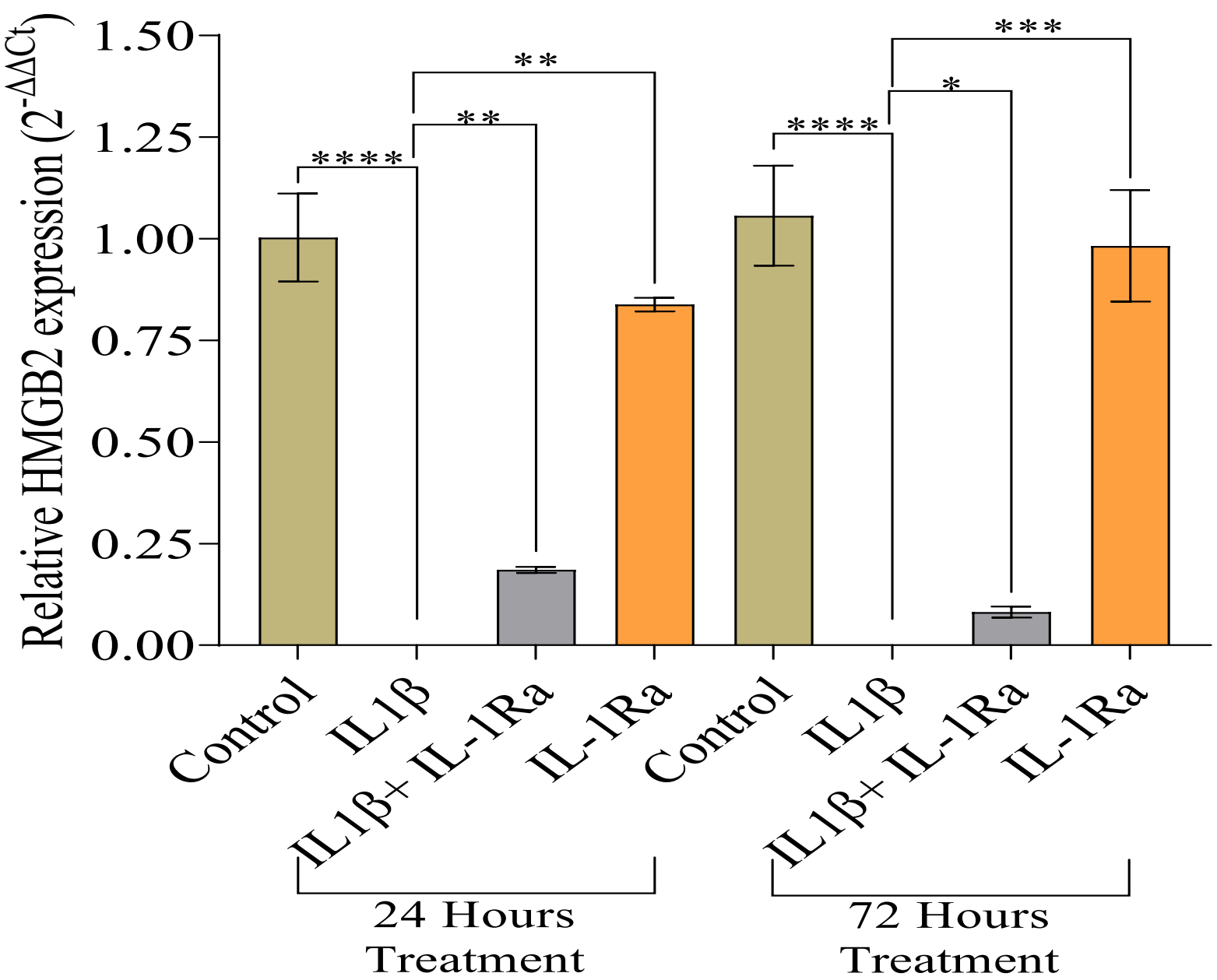

Supplementary Fig. S22 qPCR quantification of HMGB2 mRNA expression after treatment with IL-1β in the presence/absence of IL-1Ra for 24 hours and 72 hours. One-way ANOVA with Dunnett's multiple comparisons test was used to assess statistical significance (**** $p < 0.0001$, ***$p<0.001$).

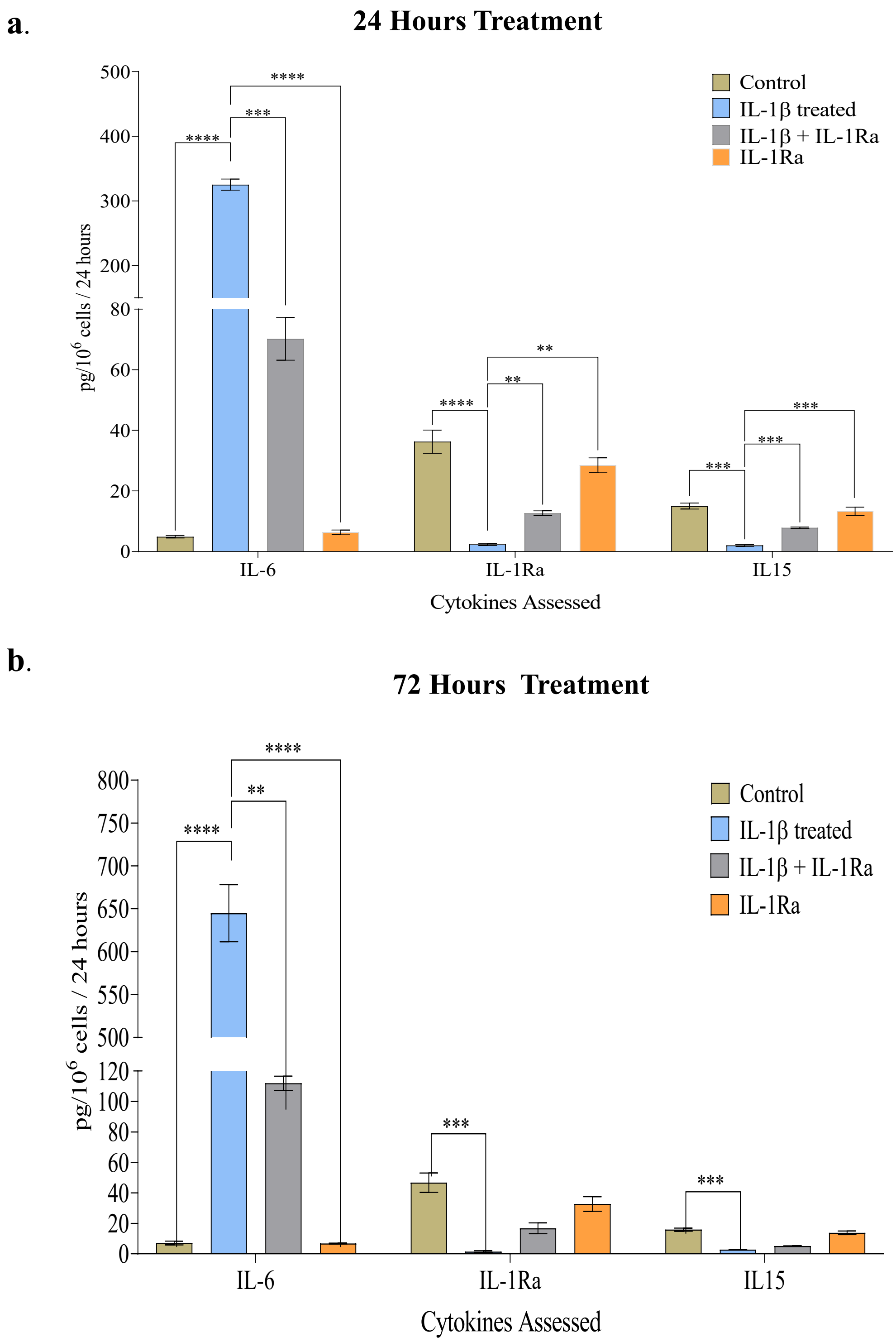

Supplementary Fig. S23 Multiplex Luminex ELISA quantification of the effect of IL-1β treatment (10 ng/ml) in the presence/absence of IL-1Ra (20ng/ml) for 24 hours and 72 hours on IL-15, IL-6 and IL-1Ra levels. One-way ANOVA with Dunnett's multiple comparisons test was used to assess statistical significance (**** $p < 0.0001$, ***$p<0.001$).

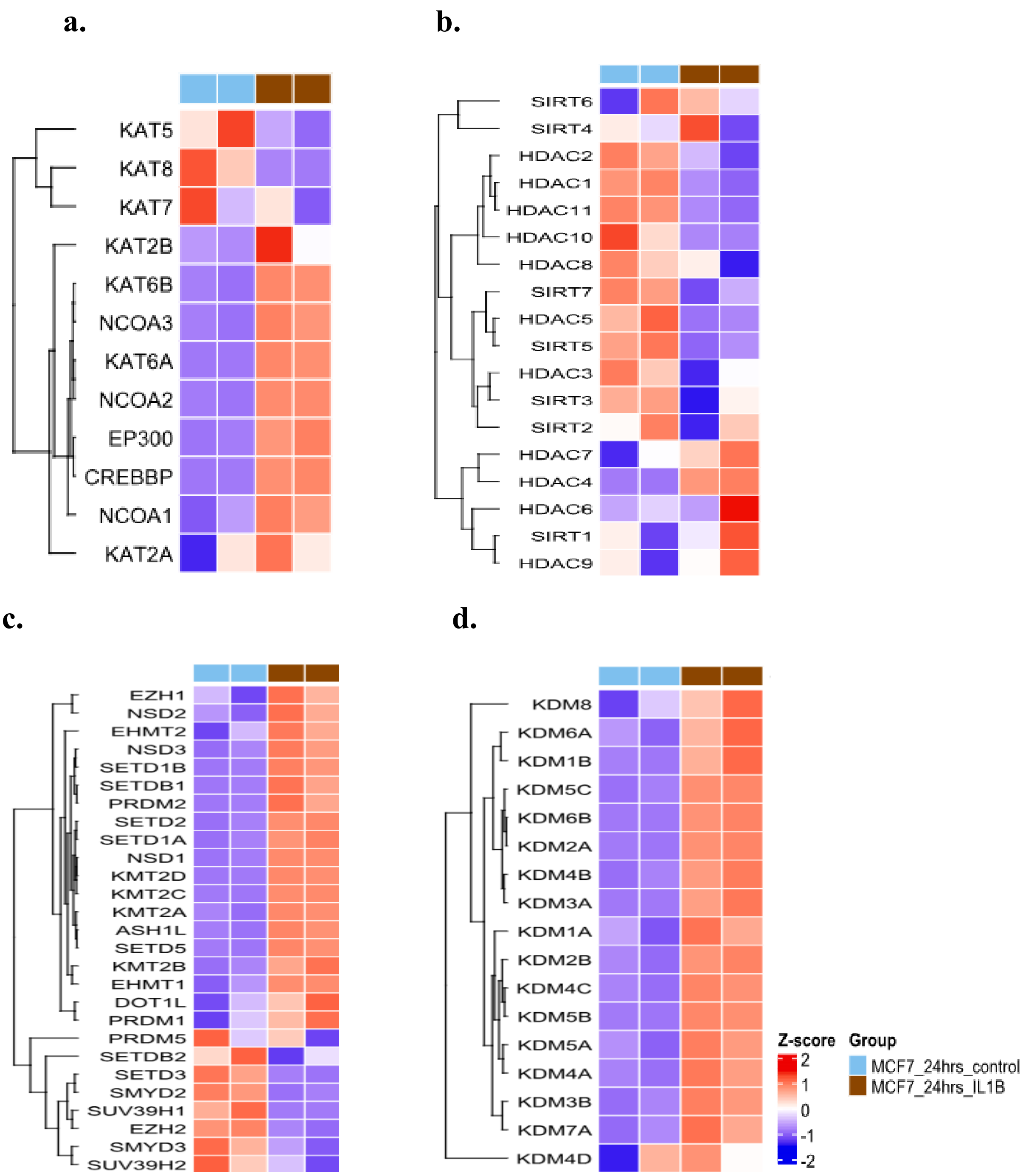

Supplementary Fig. S24 Expression Profiling of **a** Histone Acetyltransferases (HATs), **b** Histone Deacetylases (HDACs) **c** Histone methyltransferase (HMTs) and **d** Histone Demethylases (HDMTs) in MCF-7 Transcriptomic dataset post induction with IL-1β for 24 hours . Expression levels are represented as Z-score–normalized expression values. Increased expression levels of several histone acetyltransferases including *KAT6B, NCOA3, KAT6A, NCOA2, EP300, CREBBP, and NCOA1* were noted. IL-1β treatment downregulates Class I HDACs, *HDAC5, HDAC11, SIRT7, EZH2, SMYD2/3, SUV39H1/2*, and *PRDM1*, while upregulating *HDAC4/7/10, EZH1, KMT2, NSD, EHMT/SETDB, DOT1L, PRDM2*, and a broad spectrum of histone demethylases.

a. **b.**

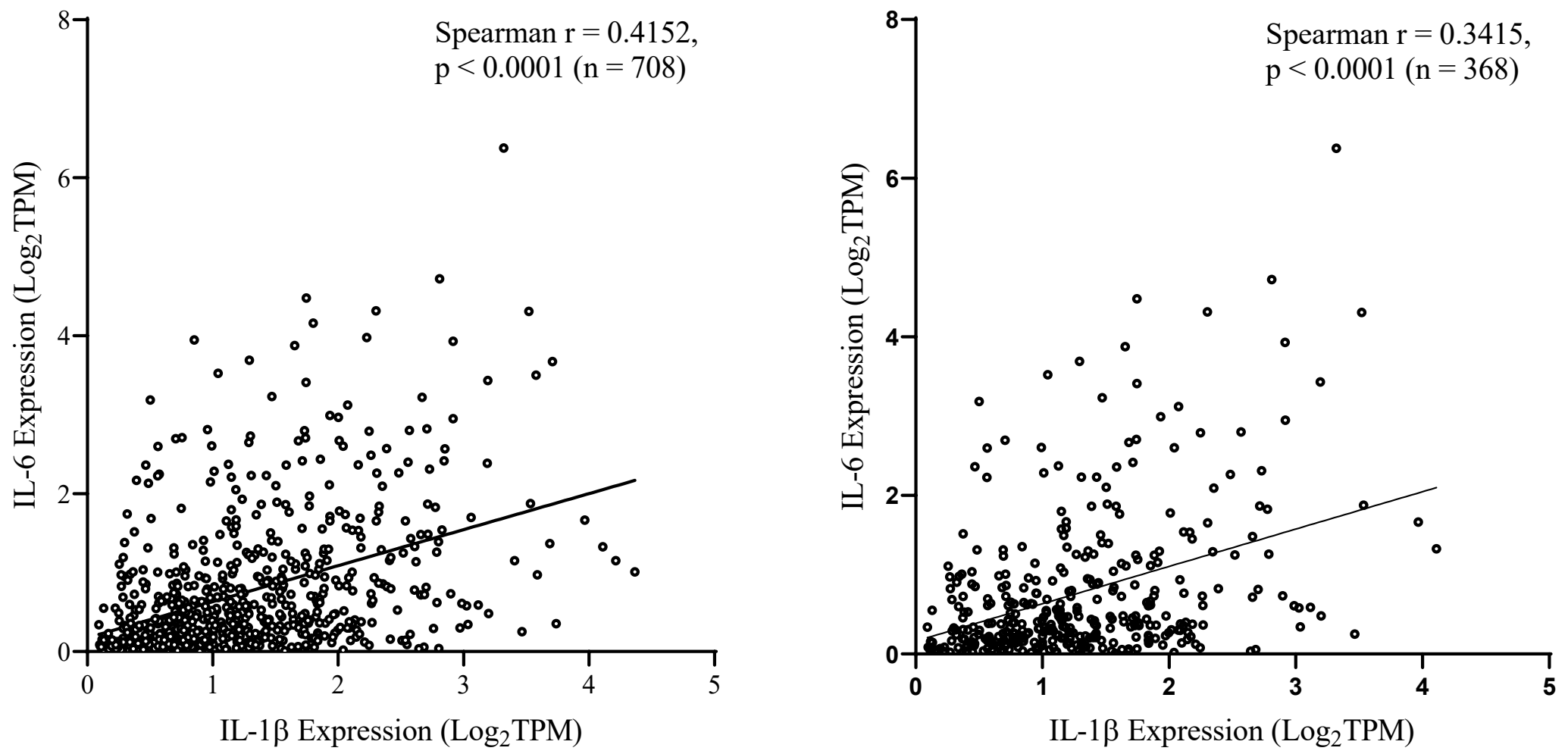

Supplementary Fig. S25 Spearman correlation between *IL1B* and *IL6* mRNA expression in TCGA-BRCA. **a** Scatter plot showing *IL1B-IL6* correlation across all primary breast tumours (n = 708), demonstrating a significant positive association (Spearman r = 0.4152, $p < 0.0001$). **b** Spearman correlation between *IL1B* and *IL6* within Luminal A tumours only (n = 368), showing a retained but slightly reduced correlation strength (r = 0.3415, $p < 0.0001$). Expression values represent FPKM. These results indicate that the IL-1β/IL-6 inflammatory axis is co-activated across breast cancers as well as within luminal A tumours specifically, supporting the biological relevance of the IL-1β/IL-6 inflammatory signalling loop observed *in vitro*.

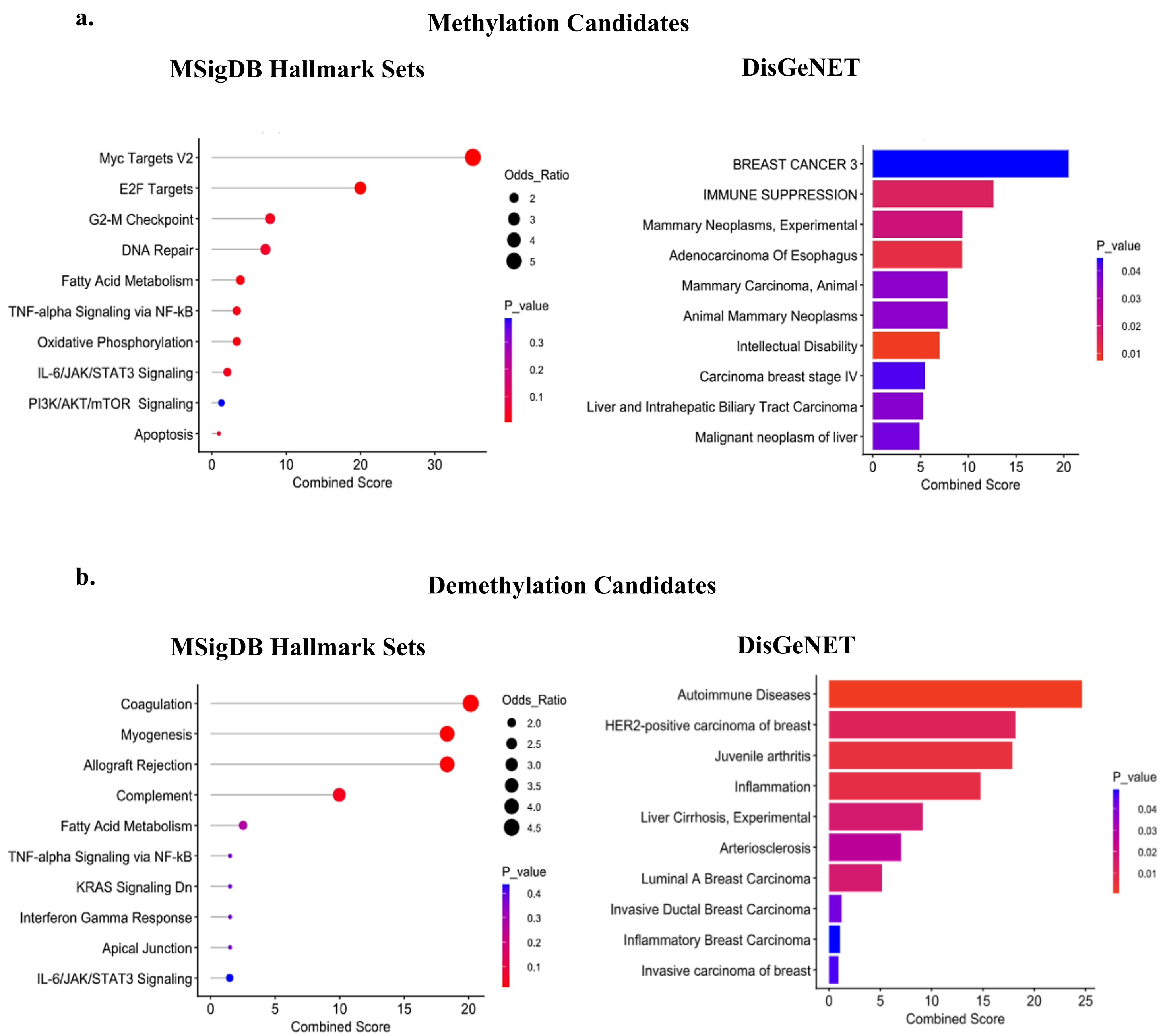

Supplementary Fig. S26 Functional enrichment analysis of putative methylation and demethylation candidate genes identified by integrating IL-6 induced transcriptomic alterations with the NCI-60 DNA methylation dataset in MCF-7 cells**.** Gene sets associated with **a.** methylation candidate genes and **b.** demethylation candidate genes were analysed using MSigDB Hallmark gene sets and DisGeNET disease enrichment. These analyses complement the GO Biological Process and KEGG pathway enrichment presented in the main manuscript (Fig. 6f).

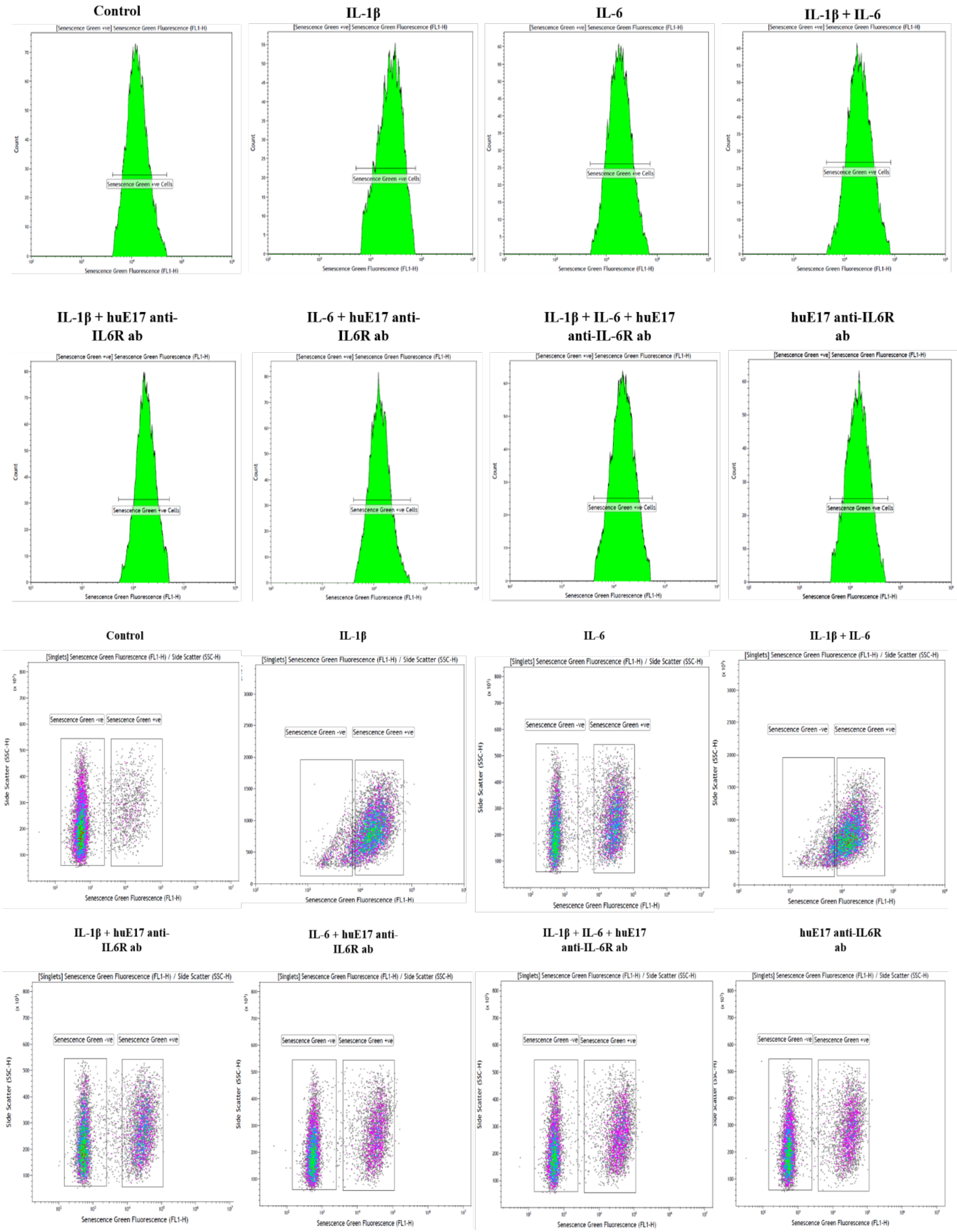

Supplementary Fig. S27 Representative histograms and scatter plots showing Senescence Green fluorescence distribution and Median Fluorescence Intensity (X-Med) following flow cytometric detection of cellular senescence via β-galactosidase hydrolysis activity in control, IL-1β, IL-6, IL-1β + IL-6, IL-1β + huE17, IL-6 + huE17, IL-1β + IL-6 + huE17 and huE17 treated MCF-7 cells after 72 hours of treatment.

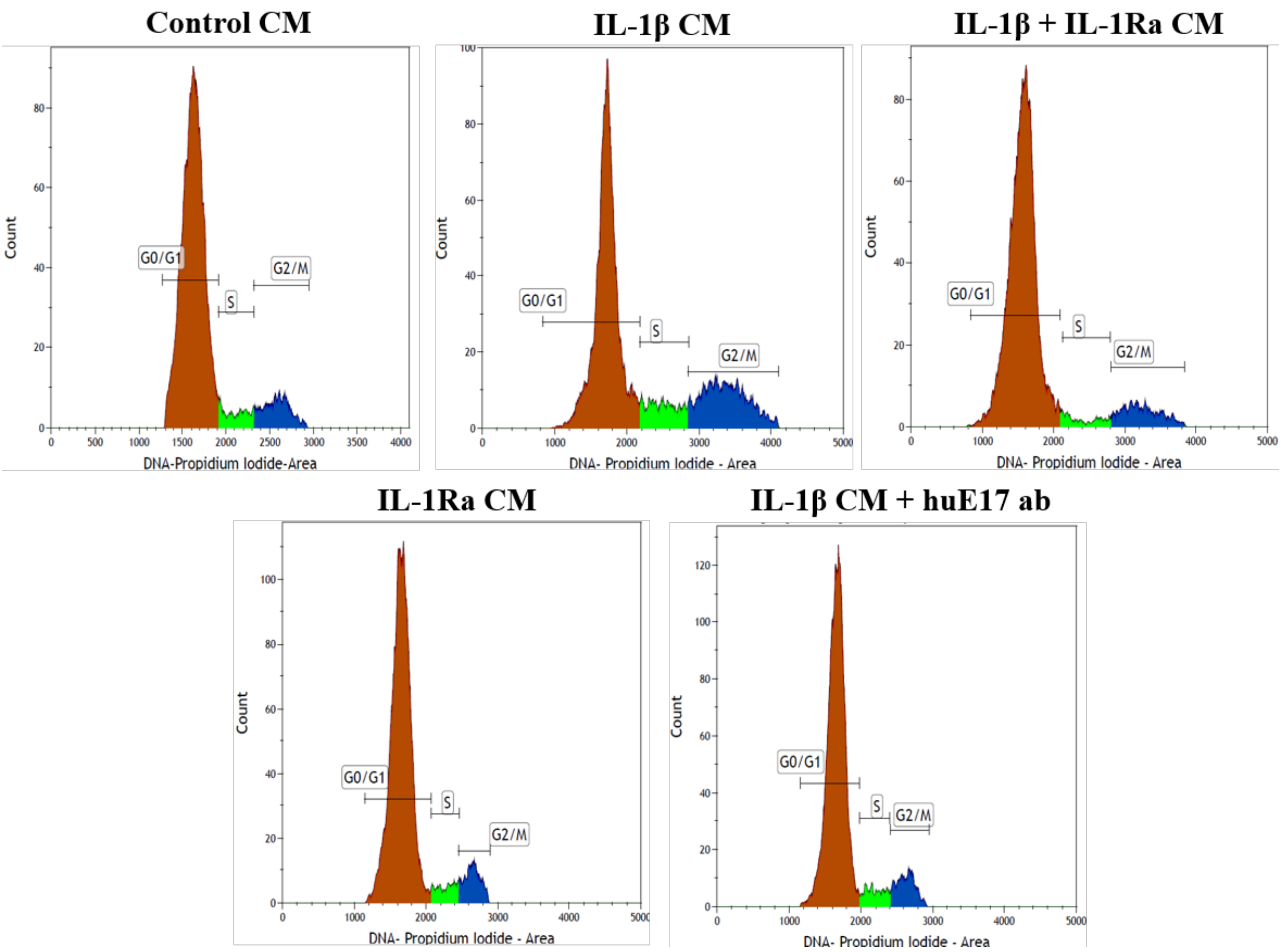

Supplementary Fig. S28 Representative flow cytometric histograms showing DNA content distribution across the G0/G1, S, and G2/M phases of the cell cycle in MCF-7 cells following 24 hours treatment with conditioned media (CM) derived from control, IL-1β, IL-1β + IL-1Ra, and IL-1Ra treated donor cells, or with IL-1β CM in the presence of the anti-IL-6R antibody huE17.

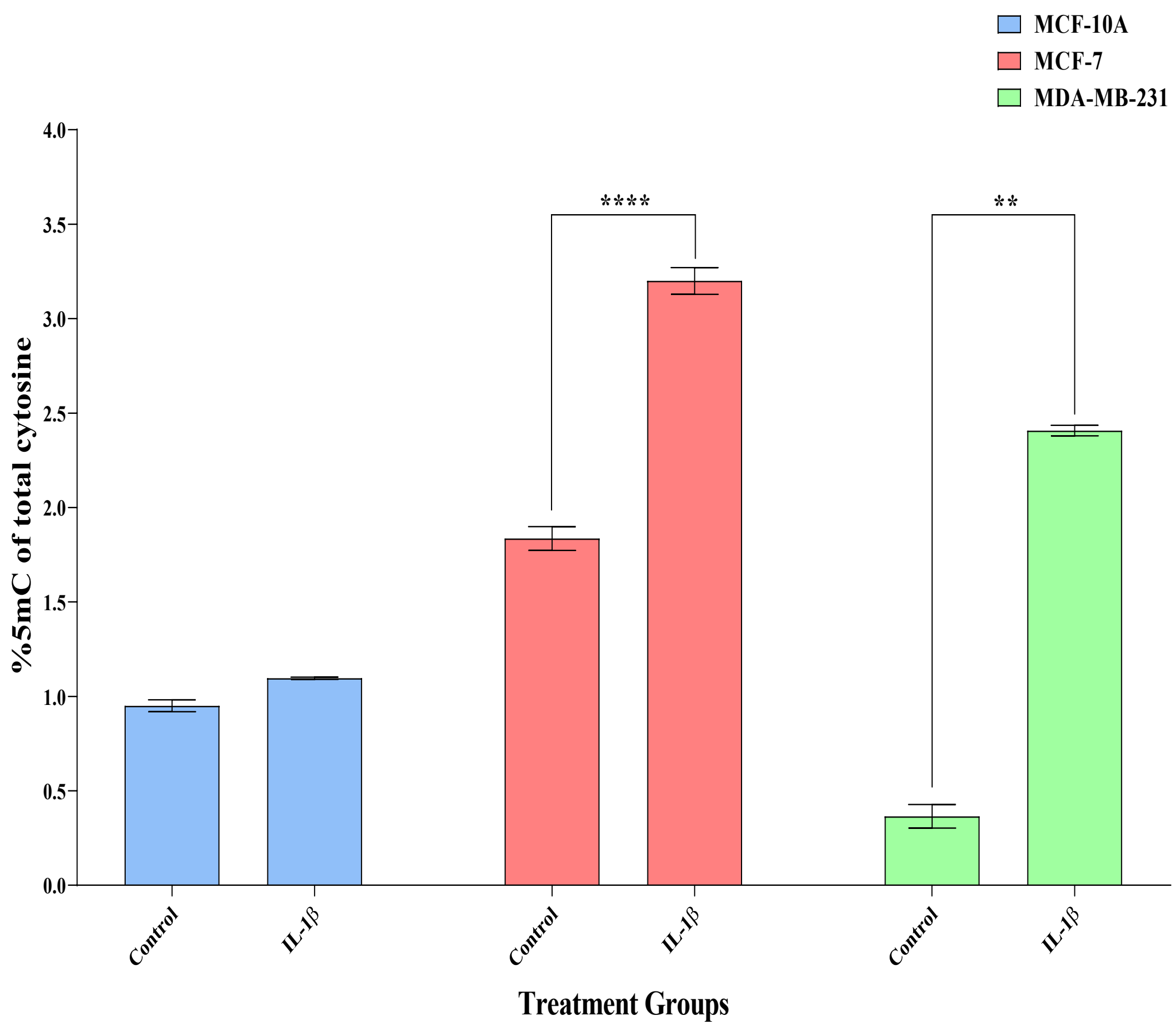

Supplementary Fig. S29 Comparative analysis of global DNA methylation following IL-1β treatment in breast epithelial non tumorigenic and breast cancer cell lines. Global DNA methylation levels were quantified by HPLC, following enzymatic digestion of genomic DNA into nucleosides. MCF-10A, MCF-7 and MDA-MB-231 cells were treated for 24 hours with IL-1β. The percentage of 5-methylcytosine (%5mC) relative to total cytosine was calculated from chromatographic peak areas. Data are presented as mean ± SD of three biological replicates. Statistical significance was determined using two-way ANOVA followed by Dunnett's multiple comparisons test, with each treatment group compared to the untreated control (*$p < 0.05$, **$p < 0.01$, ***$p < 0.001$, ****$p < 0.0001$). The MCF-7 dataset corresponds to that presented in Figure 3 and is included here to facilitate comparison across the three cell models.

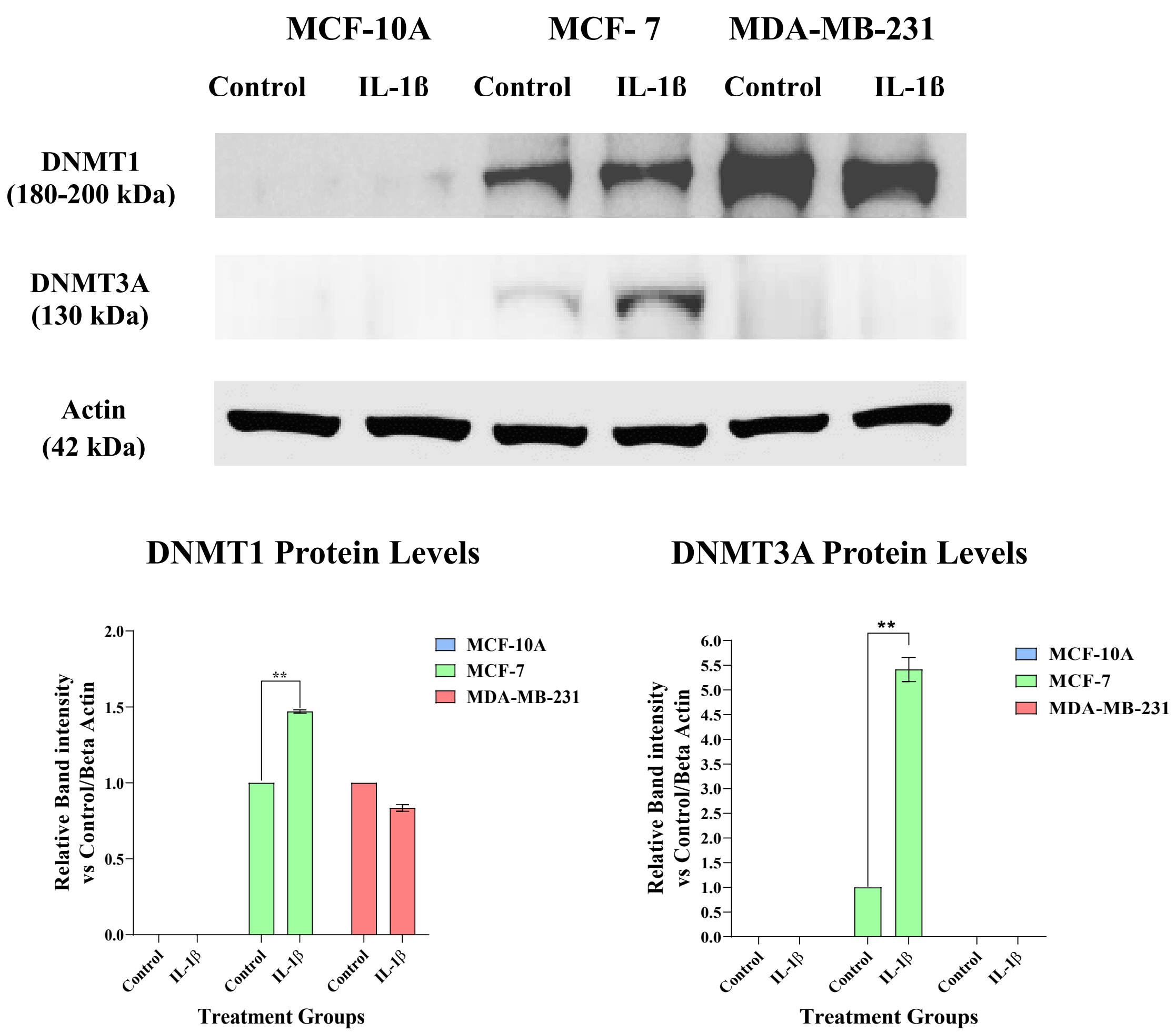

Supplementary Fig. S30 Comparative expression of DNA methyltransferases following 24 hours of IL-1β treatment in breast epithelial non tumorigenic and breast cancer cell lines. Representative western blots and densitometric analyses of DNMT1 and DNMT3A protein expression in MCF10A, MCF7 and MDA-MB-231 cells following treatment with IL-1β (10 ng/mL) for 24 hours. Equal amounts of total protein were resolved by SDS-PAGE, transferred onto PVDF membranes and probed with antibodies against DNMT1, DNMT3A and β-actin, which served as the loading control. Band intensities were quantified by densitometry and normalized to β-actin. Data are presented as mean ± SD from three independent experiments. Statistical significance was determined using two-tailed unpaired *t*-tests with Welch's correction (*P < 0.05, **P < 0.01, ***P < 0.001, ****P < 0.0001). The MCF-7 immunoblots correspond to those presented in Figure 3 and are included here for direct comparison across the three cell models.

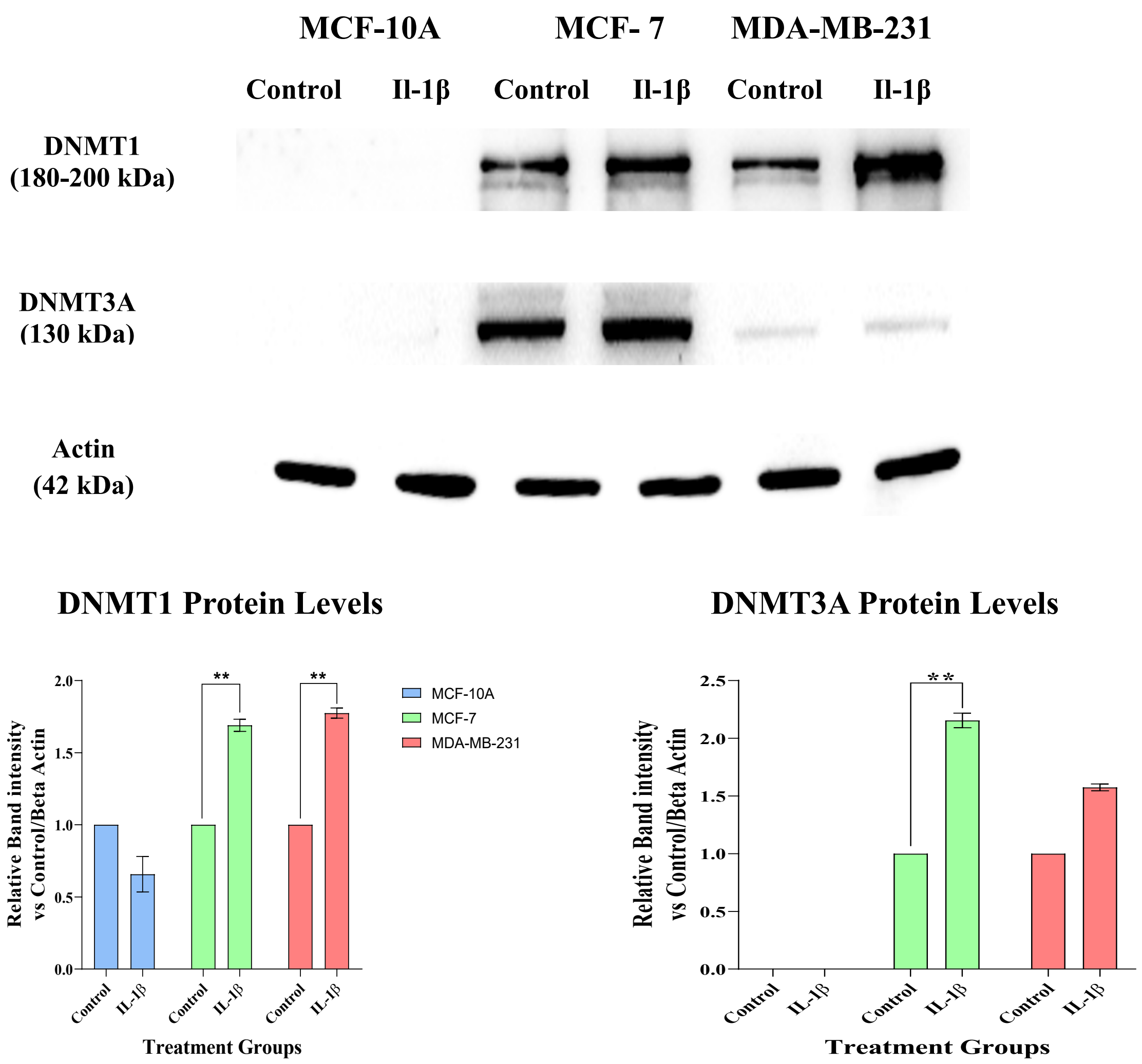

Supplementary Fig. S31 Comparative expression of DNA methyltransferases following 72 hours of IL-1β treatment in breast epithelial non tumorigenic and breast cancer cell lines. Representative western blots and densitometric analyses of DNMT1 and DNMT3A protein expression in MCF10A, MCF7 and MDA-MB-231 cells following treatment with IL-1β (10 ng/mL) for 72 hours. Cells received fresh cytokine-containing medium every 24 hours throughout the treatment period. Equal amounts of total protein were resolved by SDS-PAGE, transferred onto PVDF membranes and probed with antibodies against DNMT1, DNMT3A and β-actin, which served as the loading control. Band intensities were quantified by densitometry and normalized to β-actin. Data are presented as mean ± SD from three independent experiments. Statistical significance was determined using two-tailed unpaired *t*-tests with Welch's correction (*P < 0.05, **P < 0.01, ***P < 0.001, ****P < 0.0001). The MCF-7 immunoblots correspond to those presented in Figure 3 and are included here for direct comparison across the three cell models.

(A)

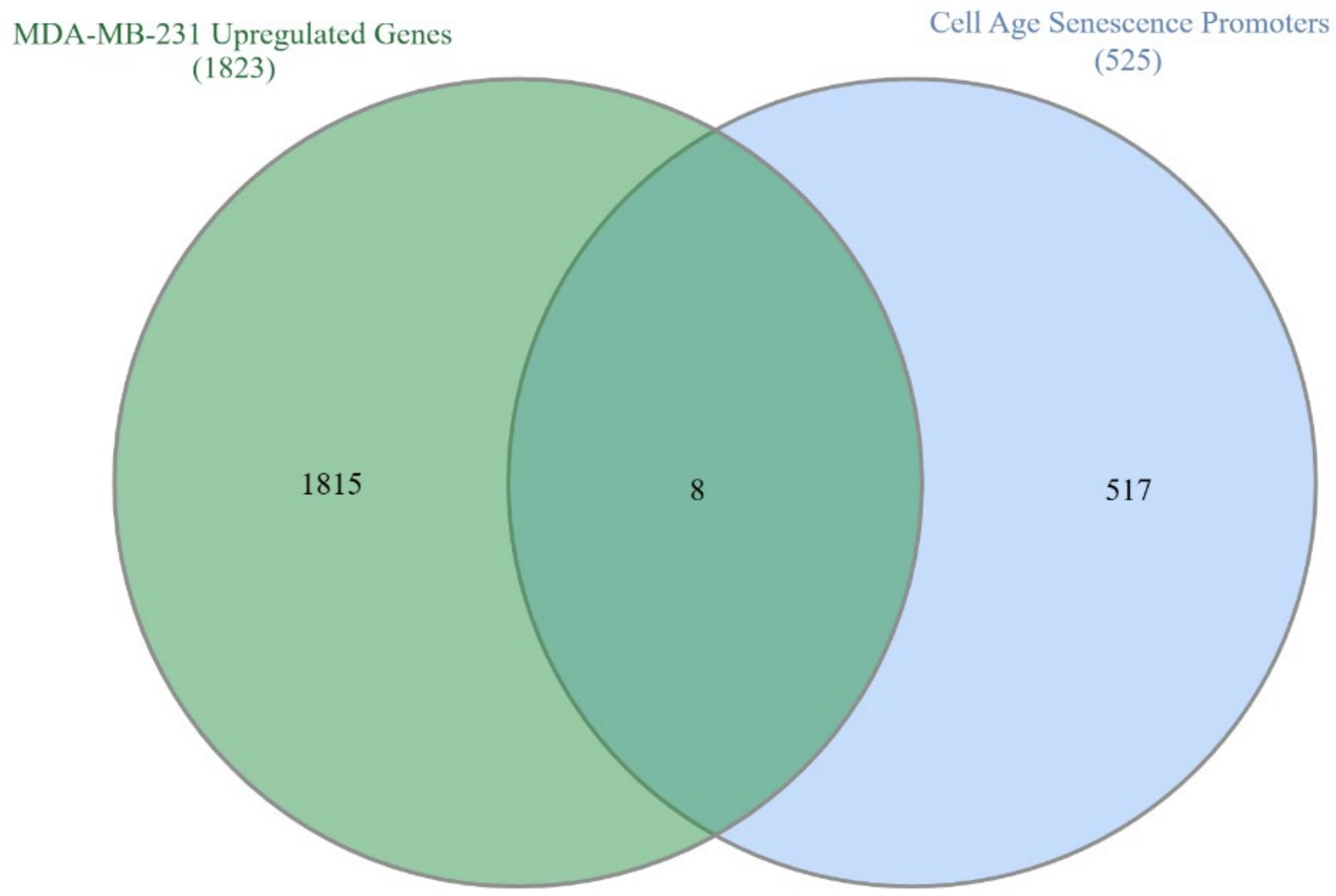

(B)

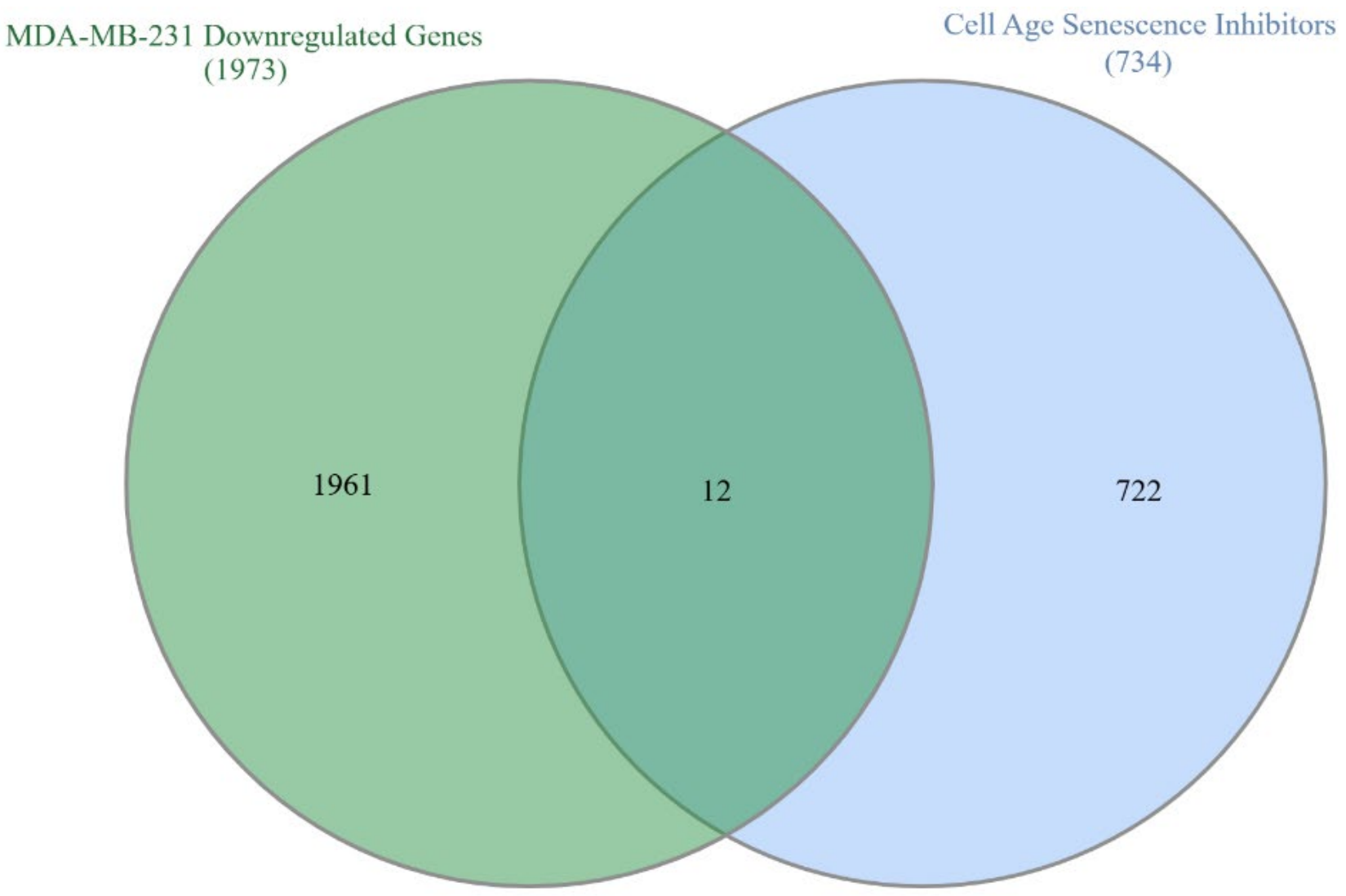

Supplementary Fig. S32 Comparative CellAge analysis of IL-1β regulated genes in MDA-MB-231 cells following 72 hours of treatment. (A) Venn diagram illustrating the overlap between genes upregulated following IL-1β treatment and CellAge senescence-promoting genes. (B) Venn diagram illustrating the overlap between genes downregulated following IL-1β treatment and CellAge senescence-inhibiting genes. Differentially expressed genes were obtained from RNA sequencing analysis of MDA-MB-231 cells treated with IL-1β (10 ng/mL) for 72 hours using an adjusted *P* value < 0.05 and |log2 fold change| ≥ 1. CellAge senescence-associated gene lists were downloaded from the CellAge database. The limited overlap observed indicates that, despite IL-1β-induced epigenetic alterations, MDA-MB-231 cells exhibit only a modest senescence-associated transcriptional response compared with MCF7 cells.
